# Supplementary material for: Currently prescribed drugs in the UK that could upregulate or downregulate ACE2 in COVID-19 disease: a systematic review
Source: BMJ Open. 2020 Sep 14;10(9):e040644. doi: 10.1136/bmjopen-2020-040644 (PMC7490921; doi:10.1136/bmjopen-2020-040644)
Supplement: Supplementary data [file bmjopen-2020-040644supp001.pdf]

Supplementary 1: Summary of search strategy

Terms	Database	Number of articles
(ace2[Title/Abstract]) OR ace 2[Title/Abstract] Filters: English	Medline (PubMed)	1980
(ace2 or ace 2).ab. and English.lg.	Embase	2880
ace2 in Title Abstract Keyword OR ace-2 in Title Abstract Keyword - (Word variations have been searched)	Cochrane	35 trials and 1 review
(TS=(ace2 OR ace-2)) AND LANGUAGE: (English) AND DOCUMENT TYPES: (Article)	Web of science	1,931
