# Supplementary material for: Currently prescribed drugs in the UK that could upregulate or downregulate ACE2 in COVID-19 disease: a systematic review
Source: BMJ Open. 2020 Sep 14;10(9):e040644. doi: 10.1136/bmjopen-2020-040644 (PMC7490921; doi:10.1136/bmjopen-2020-040644)
Supplement: Supplementary data [file bmjopen-2020-040644supp002.pdf]

Supplementary Material 2: Table of studies at baseline

Study	Country	Exposure drug(s)	Duration of exposure	Subject / model tested	Site of ACE2 receptors measured	Sample size	Condition of subject / model
Abdel-Fattah 2018	Egypt	Telmisartan or Nimodipine	15 days	Rat	Cerebral	117	Healthy
Abdelkader 2020	Egypt	Telmisartan and 17B-estradiol	6 weeks	Rat	Cerebral	75	Alzheimer's mouse model
Abe 2015	Japan	Olmesartan	24 weeks	Human	Renal	31	Diabetic
Abuohashish 2017	Egypt	Captopril	6 weeks	Rat	Bone	Not stated	Oestrogen deficient
Agata 2006	Japan	Olmesartan	4 weeks	Rat	Cardiac	Not stated	Hypertension
Aguilar 2011	Spain	atorvastatin	8 weeks	Rat	Cardiac	Not stated	Diabetic
Ali 2018	Netherlands	Pioglitazone or Vitamin D	14 days	Rat	Renal	Not stated	Renal Disease
Andersen, 2015	Denmark	Vitamin d	3 weeks	Rat	Not stated	33	Not stated
Araujo 2018	Brazil	Olmesartan	10 days	Hamster	Oral	10	Not stated
Arumugan 2010	Japan	Candesartan	4 weeks	Rat	Cardiac	Not stated	Healthy
Awwad 2019	Egypt	Pregabalin	Not stated	Rat	Cerebral	Not stated	Healthy
Awwad 2019	Egypt	Telmisartan or Captopril	21 days	Rat	Cardiac	8	Heart failure
Azis 2019	Malaysia	Captopril/ Losartan	4 weeks	Rat	Endothelial cells	7	Hypertension
Badae 2019	Egypt	Enalapril	4 weeks	Rat	Cardiac	Not stated	Heart disease
Bernardi 2015	Not stated	Eplerenone	4 weeks	Rat	kidney	75	Renal disease
Bukowska 2017	Germany	Oestrogen	24 hours	Human	Cardiac	36	Heart disease
Burchill 2008	Australia	Ramipril	10 days	Rat	Cardiac	38	Nephrectomy
Chen 2015	China	Perindopril or Losartan	12 weeks	Human	Kidney	110	Diabetic + chronic kidney disease

Chodavarapu 2013	USA	Rosiglitazone	8 weeks	Mouse	Not stated	8	Diabetic
Dong 2019	China	Spironolactone	8 weeks	Rat	kidney	85	Diabetic
Fagyas 2011	Hungary	Enalapril, Perindopril or Ramipril	Not stated	Human	unclear	375	Not stated
Feng 2020	China	Candesartan & Perindopril	4 weeks	Rat	Cerebral	Not stated	Not stated
Flores Monroy, 2016,	USA	Captopril	48 hours	Rat	heart	Not stated	Heart disease
Fuchs 2018	USA	Nicotine	3 weeks	Rat	Cerebral	110	Not stated
Gallagher 2008	USA	Losartan	12 hours	Rat	Cardiac	Not stated	Not stated
Gebska 2013	Poland	Atorvastatin	24 hours	Rat	Cardiac	Not stated	Healthy
Graus-Nunes 2019	Brazil	Losartan or Telmisartan	10 weeks	Mouse	Hepatic	20	Not stated
Guo, 2016	China	Telmisartan	3 weeks	Rat	Pulmonary	Not stated	Not stated
Gupta 2012	USA	Ivabradine	3 months	Dog	Cardiac	24	Heart failure model
Hao 2013,	China	Imidapril	30 days	Chicken	Cardiac	24	Not stated
Hermenegildo 2015	Spain	Estradiol	1 day	Human	Not stated	Not stated	Not stated
Hermenegildo 2018	Argentina	Estradiol	24 hours	Human	Not stated	Not stated	Healthy
Hiroi 2014	Japan	Olmesartan	3 months	Human	Serum	8	HTN
Ibarra-Lara 2016	Mexico	Captopril	21 days	Rat	Cardiac	Not stated	heart disease
Ichikawa 2018	Japan	Olmesartan	2 months	Mouse	Renal	Not stated	Not stated
Igase 2005	USA	Olmesartan, Atenolol, or Hydralazine	14 days	Rat	Cardiac	60	Hypertensive
Igase 2008	Japan	Olmesartan	14 days	Rat	Cardiac	10	Hypertensive
Iizuka 2009	Japan	Nifedipine	Not stated	Human	Cardiac	Not stated	Healthy cells
Ishibashi 2014	Japan	Olmesartan	Not stated	Human	Renal	Not stated	Not stated

Ishiyama 2004	Not known	Losartan Olmesartan	28 days	Rat	Cardiac	62	Post myocardial infarction
Iwanami 2013	Japan	Azilsartan or Olmesartan	4 weeks	Mouse	Cardiac and renal	10	Healthy
Jeong 2018	South Korea	Insulin or pravastatin	8 weeks	Rat	Cardiac	87	Type 1 diabetic
Jessup 2006	USA	Lisinopril or Losartan	12 days	Rat	Cardiac and renal	27	Hypertensive
Jessup 2008	USA	Hydrochlorothiazide	7 days	Rat	Cardiac	48	Hypertensive
Kaiqiang 2009	Japan	Olmesartan	16 weeks	Rat	Cardiac	16	Cardiac hypertrophy
Kidoguchi 2019	Japan	Azilsartan	9 weeks	Rat	Renal	51	Healthy
Kong 2019	China	Spironolactone	Not stated	Rat	Renal	Not stated	Liver Disease
Li 2011	China	Irbesartan	12 weeks	Rat	Cardiac	39	Diabetic
Li 2013	China	Rosuvastatin	29 days	Rat	Cardiac	36	Unclear
Li 2016	China	Valsartan	28 days	Rat	Cardiac	36	Aortic injury
Liang 2015	USA	Telmisartan	35 days	Rat	Cardiac	90	Heart failure
Lijuan 2013	China	Olmesartan	3 days	Mouse	Hepatic	42	Not stated
Lin 2016	China	Calcitriol	Not stated	Rat	Renal	25	Diabetic
Ma 2018	China	Irbesartan, benazepril	12 weeks	Human	Vaginal	36	Not stated
Machado 2014	Brazil	Vitamin d	12 weeks	Rat	Cardiac	Not stated	Hypertension
Malek 2019	India	Telmisartan	4 weeks	Rat	Renal	56	Diabetes
Mao-liang Huang 2009	China	perindopril	4 weeks	Rat	Hepatic	30	Healthy
Marquez 2013	Spain	Insulin	14 days	Rat	Renal	Not stated	Diabetic
Marquez 2014	Spain	Insulin	2 days	Mouse	Renal	Not stated	Healthy
Ocaranza 2006	Chile	Enalapril	8 weeks	Rat	Cardiac	83	Not stated
Ohshima 2014	Japan	Azilsartan	2 weeks	Rat	Cardiac	8	Not stated
Onat 2018	Turkey	Amlodipine or Rosuvastatin	4 weeks	Rat	Renal	Not stated	Hypertension

Qiao 2015	China	Ibuprofen	8 weeks	Rat	Cardiac	18	Diabetes
Riera 2014	Spain	Insulin	40 days	Mouse	Serum, urine and renal	30	Diabetes
Romani-Perez 2015	Spain	Liraglutide	14 days	Rat	Pulmonary	52	Diabetes
Sabry 2018	Egypt	Losartan	6 weeks	Rat	Adipose tissue	48	Diabetes
Salem 2012	USA	Insulin	24 hours	Mouse	Renal	Not stated	Type 1 diabetes
Salem 2013	USA	Insulin	30 days	Mouse	Renal	8	Type 1 diabetes
Salem 2014	USA	Insulin	20 weeks	Mouse	Renal	Not stated	Type 1 diabetes
Sanchez Aguilar 2018	Mexica	Rosiglitazone	7 days	Rat	renal	Not stated	Hypertension
Sanchez-Aguilar 2019	Mexico	Rosiglitazone	7 days	Rat	Cardiac	12	Aortic coarctation model
Scroggin 2012	USA	Rosiglitazone	1 day	Human	Cerebral	Not stated	Not stated
Senador 2010	USA	Losartan	8 weeks	Mouse	Renal	12	Not stated
Shenoy 2009	USA	17b-estradiol (E2)	6 weeks	Rat	Cardiac	18	Bilaterally ovariectomized & hypertensive
Shimada 2011	Japan	Olmesartan	2 weeks	Rat	Cerebral	Not stated	Oestrogen-deficient
Shin 2017	China	insulin	8 weeks	Rat	Cardiac	84	Diabetes
Soler 2009	USA	Telmisartan	2 weeks	Mouse	Renal	11	Not stated
Song 2012	USA	Candesartan	19 days	Rat	Renal	Not stated	Not stated
Speth 2014	USA	Zinc	Real time	Rat	Renal and Pulmonary	Not stated	Not stated
Suh 2019	South Korea	Olmesartan	4 weeks	Mouse	Renal	16	Alport mouse model
Sukumaran 2012	Japan	Telmisartan	4 weeks	Rat	Cardiac	18	Induced cardiomyopathy
Takai 2013	Japan	Valsartan; valsartan & amlodipine	2 weeks	Rat	Cardiac	24	Hypertension

Tanno 2016	Japan	Olmesartan & hydralazine	2 months	Mouse	Cardiac	6	Over-expressing renin
Thanekar 2019	USA	Canagliflozin	15 weeks	Mouse	Urine	Not stated	Diabetes
Varagic 2012	USA	Nebivolol	Not stated	Rat	Cardiac	44	Hypertension
Varagic 2012b	USA	Olmesartan	6 weeks	Rat	Renal	28	Hypertension
Wang 2012	China	Fosinopril	28 days	Rat	Cardiac	32	Heart Disease
Wang 2013	China	Oestrogen	4 weeks	Mouse	Ovarian	17	Hypertension
Wang 2015	China	Sildenafil	30 minutes	Rat	Cardiac	32	Healthy
Wang 2016	China	Enalapril	30 minutes	Rat	Cardiac	32	Healthy
Wang 2016	China	Olmesartan, Candesartan, Telmisartan, Losartan, Valsartan or Irbesartan	Not stated	Mouse	Cardiac	54	Healthy
Wang 2018	China	Telmisartan	12 weeks	Mouse	Cardiac	32	Healthy
Weili 2014	China	Ibuprofen, pioglitazone	8 weeks	Rat	Cardiac	Not stated	Not stated
Wosten-van-Asperen 2011	USA	Losartan	4 hours	Rat	Pulmonary	48	Acute respiratory distress syndrome
Wu 2015	China	Losartan or Enalapril	Not stated	Rat	Cardiac	Not stated	Aortic coarctation model
Xiao 2016	China	Captopril	Not stated	Pig	Not known	Not stated	Not stated
Xu 2017	China	Calcitriol	Not stated	Rat	Pulmonary	30	Lung injury
Yang 2013	China	Enalapril	4 weeks	Rat	Cardiac	25	Hypertension
Yang 2018	China	Telmisartan	24 weeks	Rat		33	Hypertension
Yisireyili 2018	Japan	Irbesartan	2 weeks	Mouse	Intestinal	48	Stress-induced mice
Young 2014		Losartan	72 hours	Mouse	Renal	Not stated	Kidney disease
Zhang 2011	China	Enalapril or Losartan	2 weeks	Mouse	Cardiac	40	Hydronephrotic
Zhang 2013	China	Pioglitazone	24 weeks	Rat	Serum and Hepatic	30	Healthy

Zhang 2014	China	Enalapril or Losartan	4 weeks	Rat	Cardiac	40	Aortic ligation
Zhang 2015	China	Losartan	12 weeks	Rabbit	Cardiac	27	Atherosclerosis
Zhao 2015	China	Linagliptin or liraglutide	28 days	Rat	Cardiac	24	Healthy
Zhao 2019	China	Valsartan	12 weeks	Rat	Cardiac	40	Hypertension
Zhong 2010	China	Telmisartan	10 weeks	Rat	Cardiac	Not stated	Hypertension
Zhong 2011	China	Telmisartan	10 weeks	Rat	Cardiac	40	Hypertension
Zhonghua 2013	China	Telmisartan	4-12 weeks	Human	Not stated	62	Hypertension + Diabetes
