# Supplementary material for: Currently prescribed drugs in the UK that could upregulate or downregulate ACE2 in COVID-19 disease: a systematic review
Source: BMJ Open. 2020 Sep 14;10(9):e040644. doi: 10.1136/bmjopen-2020-040644 (PMC7490921; doi:10.1136/bmjopen-2020-040644)
Supplement: Supplementary data [file bmjopen-2020-040644supp003.pdf]

Supplementary Material 3: Risk of bias assessment

Study	Was the allocation sequence adequately generated and applied?	Were the groups similar at baseline or were they adjusted for confounders in the analysis?	Was the allocation adequately concealed?	Were the animals randomly housed during the experiment?	Were the caregivers and/or investigators blinded from knowledge which intervention each animal received during the experiment?	Were animals selected at random for outcome assessment?	Was the outcome assessor blinded?	Were incomplete outcome data adequately addressed?	Are reports of the study free of selective outcome reporting?	Was the study apparently free of other problems that could result in high risk of bias?
Abdel-Fattah 2018	Red	Red	Yellow	Yellow	Yellow	Red	Yellow	Green	Green	Red
Abdelkader 2020	Yellow	Yellow	Yellow	Yellow	Green	Yellow	Yellow	Red	Yellow	Red
Abe 2015	Yellow	Yellow	Yellow	Yellow	Yellow	Yellow	Red	Red	Red	Red
Abuohashish 2017	Yellow	Red	Yellow	Red	Red	Red	Red	Red	Red	Red
Agata 2006	Yellow	Yellow	Yellow	Red	Red	Red	Red	Red	Red	Red
Anderson, 2015	Yellow	Yellow	Yellow	Red	Red	Red	Red	Red	Red	Red
Araujo 2018	Yellow	Yellow	Yellow	Yellow	Red	Red	Red	Red	Red	Red
Arumigan 2010	Yellow	Yellow	Yellow	Red	Red	Red	Red	Red	Red	Red
Awwad 2019	Yellow	Yellow	Yellow	Red	Red	Yellow	Yellow	Red	Red	Red
Awwad 2019	Red	Green	Green	Green	Yellow	Green	Yellow	Yellow	Red	Red
Badae 2019	Yellow	Yellow	Yellow	Yellow	Red	Red	Red	Red	Red	Red
Bernadi 2015	Red	Red	Yellow	Yellow	Red	Yellow	Red	Red	Red	Red
Bukowska 2017	Yellow	Yellow	Yellow	Yellow	Yellow	Red	Red	Red	Red	Red
Burchill 2008	Yellow	Yellow	Yellow	Yellow	Red	Red	Red	Red	Red	Red
Chen 2015	Yellow	Yellow	Yellow	Yellow	Yellow	Yellow	Yellow	Red	Yellow	Red
Chodavarapu 2013	Yellow	Yellow	Yellow	Yellow	Yellow	Yellow	Yellow	Red	Yellow	Red
Dong 2019	Red	Green	Yellow	Yellow	Yellow	Yellow	Yellow	Yellow	Yellow	Red
Fangyas 2011	Red	Red	Red	Yellow	Red	Red	Red	Red	Red	Red
Feng 2020	Yellow	Yellow	Yellow	Yellow	Yellow	Yellow	Yellow	Red	Yellow	Red
Fuchs 2018	Red	Red	Green	Yellow	Red	Yellow	Yellow	Red	Red	Red
Furuhashi 2014	Red	Red	Red	Red	Red	Red	Red	Red	Red	Red
Furuhashi 2015	Yellow	Yellow	Red	Red	Red	Red	Red	Red	Red	Red
Gallagher 2008	Yellow	Yellow	Yellow	Yellow	Yellow	Yellow	Yellow	Red	Yellow	Red
Gebska 2013	Red	Red	Red	Red	Red	Red	Red	Red	Red	Red
Graus-Nunes 2019	Yellow	Yellow	Yellow	Yellow	Yellow	Yellow	Yellow	Yellow	Red	Red
Guo 2016	Red	Red	Red	Red	Red	Red	Red	Red	Red	Red
Gupta 2012	Green	Red	Green	Green	Green	Green	Green	Red	Red	Red

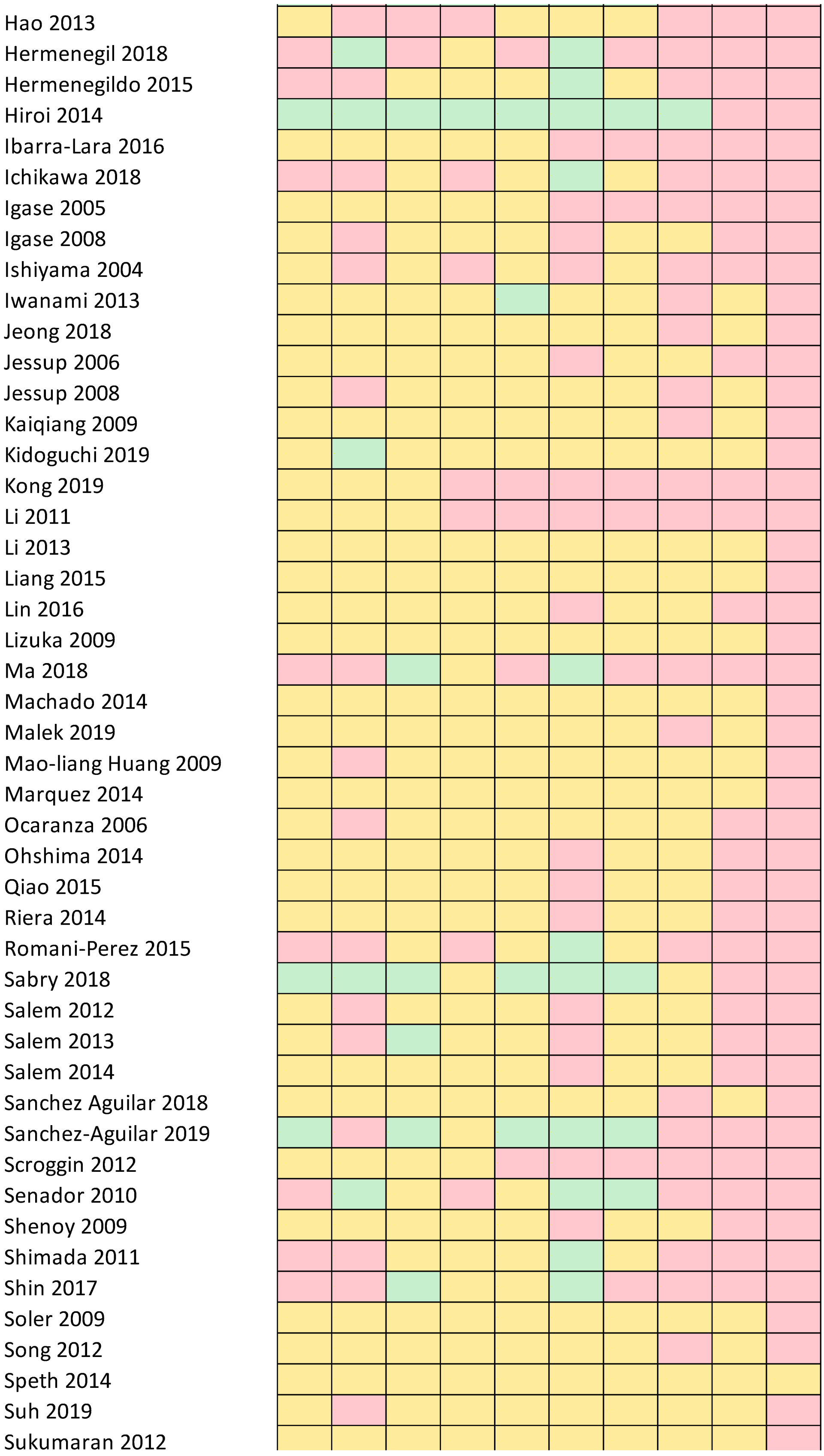
Hao 2013
Hermenegil 2018
Hermenegildo 2015
Hiroi 2014
Ibarra-Lara 2016
Ichikawa 2018
Igase 2005
Igase 2008
Ishiyama 2004
Iwanami 2013
Jeong 2018
Jessup 2006
Jessup 2008
Kaiqiang 2009
Kidoguchi 2019
Kong 2019
Li 2011
Li 2013
Liang 2015
Lin 2016
Lizuka 2009
Ma 2018
Machado 2014
Malek 2019
Mao-liang Huang 2009
Marquez 2014
Ocaranza 2006
Ohshima 2014
Qiao 2015
Riera 2014
Romani-Perez 2015
Sabry 2018
Salem 2012
Salem 2013
Salem 2014
Sanchez Aguilar 2018
Sanchez-Aguilar 2019
Scroggin 2012
Senador 2010
Shenoy 2009
Shimada 2011
Shin 2017
Soler 2009
Song 2012
Speth 2014
Suh 2019
Sukumaran 2012

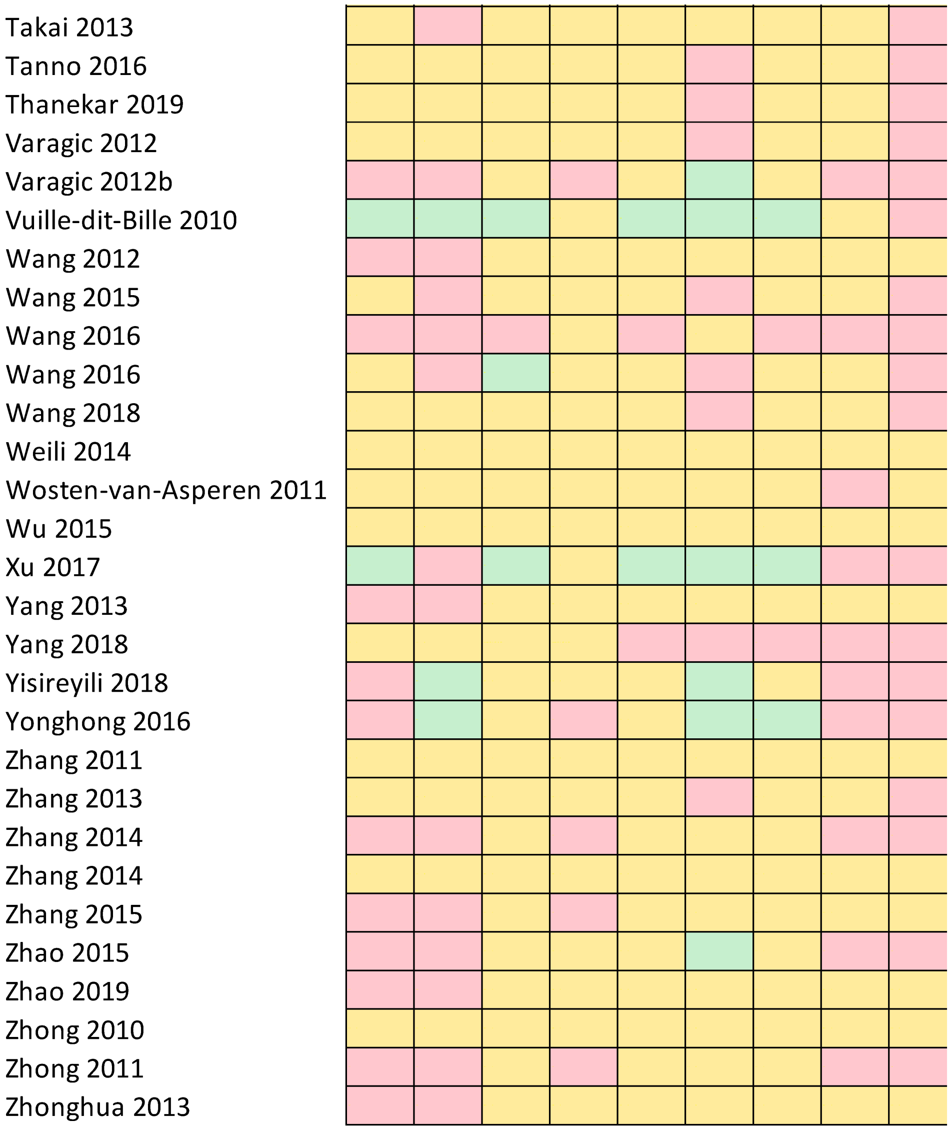
Takai 2013
Tanno 2016
Thanekar 2019
Varagic 2012
Varagic 2012b
Vuille-dit-Bille 2010
Wang 2012
Wang 2015
Wang 2016
Wang 2016
Wang 2018
Weili 2014
Wosten-van-Asperen 2011
Wu 2015
Xu 2017
Yang 2013
Yang 2018
Yisireyili 2018
Yonghong 2016
Zhang 2011
Zhang 2013
Zhang 2014
Zhang 2014
Zhang 2015
Zhao 2015
Zhao 2019
Zhong 2010
Zhong 2011
Zhonghua 2013
