# Supplementary material for: Currently prescribed drugs in the UK that could upregulate or downregulate ACE2 in COVID-19 disease: a systematic review
Source: BMJ Open. 2020 Sep 14;10(9):e040644. doi: 10.1136/bmjopen-2020-040644 (PMC7490921; doi:10.1136/bmjopen-2020-040644)
Supplement: Author's manuscript [file bmjopen-2020-040644.draft_revisions.pdf]

BMJ Open is committed to open peer review. As part of this commitment we make the peer review history of every article we publish publicly available.

When an article is published we post the peer reviewers' comments and the authors' responses online. We also post the versions of the paper that were used during peer review. These are the versions that the peer review comments apply to.

The versions of the paper that follow are the versions that were submitted during the peer review process. They are not the versions of record or the final published versions. They should not be cited or distributed as the published version of this manuscript.

BMJ Open is an open access journal and the full, final, typeset and author-corrected version of record of the manuscript is available on our site with no access controls, subscription charges or pay-per-view fees (http://bmjopen.bmj.com).

If you have any questions on BMJ Open's open peer review process please email info.bmjopen@bmj.com

BMJ Open

Currently prescribed drugs in the UK that could up or downregulate ACE2 in COVID-19 disease: A systematic review

Journal:	*BMJ Open*
Manuscript ID	bmjopen-2020-040644
Article Type:	Original research
Date Submitted by the Author:	19-May-2020

Complete List of Authors:	Dambha-Miller, Hajira; University of Southampton Faculty of Medicine, Department of Primary Care; University of Cambridge, MRC Epidemiology Unit<br>Albasri, Ali; Department of Primary Care Health Sciences, University of Oxford, ; University of Oxford<br>Hodgson, Sam; University of Southampton Faculty of Medicine, Department of Primary Care<br>Wilcox, Christopher; University of Southampton Faculty of Medicine, Department of Primary Care<br>Khan, Shareen; Oxford University Hospitals NHS Trust<br>Islam, Nazrul ; University of Oxford Nuffield Department of Population Health; University of Cambridge, MRC Epidemiology Unit<br>Little, Paul; University of Southampton, Medical School,<br>Griffin, Simon; University of Cambridge School of Clinical Medicine

Keywords:	Public health < INFECTIOUS DISEASES, Respiratory infections < THORACIC MEDICINE, Adverse events < THERAPEUTICS

SCHOLARONE™
Manuscripts

I, the Submitting Author has the right to grant and does grant on behalf of all authors of the Work (as defined in the below author licence), an exclusive licence and/or a non-exclusive licence for contributions from authors who are: i) UK Crown employees; ii) where BMJ has agreed a CC-BY licence shall apply, and/or iii) in accordance with the terms applicable for US Federal Government officers or employees acting as part of their official duties; on a worldwide, perpetual, irrevocable, royalty-free basis to BMJ Publishing Group Ltd ("BMJ") its licensees and where the relevant Journal is co-owned by BMJ to the co-owners of the Journal, to publish the Work in this journal and any other BMJ products and to exploit all rights, as set out in our licence.

The Submitting Author accepts and understands that any supply made under these terms is made by BMJ to the Submitting Author unless you are acting as an employee on behalf of your employer or a postgraduate student of an affiliated institution which is paying any applicable article publishing charge ("APC") for Open Access articles. Where the Submitting Author wishes to make the Work available on an Open Access basis (and intends to pay the relevant APC), the terms of reuse of such Open Access shall be governed by a Creative Commons licence – details of these licences and which Creative Commons licence will apply to this Work are set out in our licence referred to above.

Other than as permitted in any relevant BMJ Author's Self Archiving Policies, I confirm this Work has not been accepted for publication elsewhere, is not being considered for publication elsewhere and does not duplicate material already published. I confirm all authors consent to publication of this Work and authorise the granting of this licence.

For peer review only

Currently prescribed drugs in the UK that could up or downregulate ACE2 in COVID-19 disease: A systematic review

Short title: Drugs using ACE2 in COVID-19

Hajira Dambha-Miller[1] MRCGP, PhD, Ali Albasri[2] DPhil, Sam Hodgson [3] BM BCh, Christopher R Wilcox [4] MBBCh, Shareen Khan MPharm[5] , Nazrul Islam MBBS, PhD [6,7] , Paul Little FMedSci [8] and Simon J Griffin [9] FMedSci.

1. NIHR Academic Clinical Lecturer and GP, Division of Primary Care and Population Health, University of Southampton
2. Clinical Pharmacist and Research Fellow, Nuffield Department of Primary Care Health Sciences, University of Oxford
3. NIHR Academic Clinical Fellow, Division of Primary Care and Population Health, University of Southampton
4. NIHR Academic Clinical Fellow, Division of Primary Care and Population Health, University of Southampton
5. Specialist Pharmacist, Oxford University Hospitals NHS Foundation Trust
6. Clinical Trial Service Unit and Epidemiological Studies Unit, Nuffield Department of Population Health, University of Oxford, Oxford, United Kingdom
7. Medical Research Council Epidemiology Unit, University of Cambridge, Cambridge, United Kingdom
8. Professor of Primary Care, Division of Primary Care and Population Health, University of Southampton
9. Professor of General Practice, Department of Public Health and Primary Care, School of Clinical Medicine, University of Cambridge

Correspondence: Dr Hajira Dambha-Miller, Primary Care and Population Health, University of Southampton, Southampton, SO16 5ST, Email: hajiradambha@doctors.org.uk

Word count: 2646

For peer review

Abstract

Objective: To review evidence on routinely prescribed drugs in the UK that could up or downregulate Angiotensin Converting Enzyme 2 (ACE2) and potentially affect COVID-19 disease

Design: Systematic review

Data source: MEDLINE, EMBASE, CINAHL, the Cochrane Library and Web of Science

Study selection: Any design with animal or human models examining a currently prescribed UK drug compared to a control, placebo or sham group, and reporting an effect on ACE2 level, activity or gene expression.

Data extraction and synthesis: MEDLINE, EMBASE, CINAHL, the Cochrane Library, Web of Science and OpenGrey from inception to 1st April 2020. Methodological quality was assessed using the SYRCLE's risk of bias tool for animal studies and Cochrane risk of bias tool for human studies.

Results: We screened 3,360 titles and included 112 studies with 21 different drug classes identified as influencing ACE2 activity. Ten studies were in humans and 102 were in animal models None examined ACE2 in human lungs. The most frequently examined drugs were Angiotensin Receptor Blockers (ARBs) (n= 55) and Angiotensin-Converting Enzyme- Inhibitors (ACE-I) (n= 22). More studies reported upregulation than downregulation with ACE-I (n=22), ARBs (n=55), insulin (n=8), thiazolidinedione (n=7) aldosterone agonists (n=3), statins (n=5), oestrogens (n=5) calcium channel-blockers (n=3) GLP-1 agonists (n=2) and NSAIDs (n=2).

Conclusions: There is an abundance of academic literature and media reports on the potential of drugs that could attenuate or exacerbate COVID-19 disease. This is leading to trials of repurposed drugs and uncertainty amongst patients and clinicians concerning continuation or cessation of prescribed medications. Our review indicates that the impact of currently prescribed drugs on ACE2 has been poorly studied in-vivo, particularly in human lungs where the SARS-CoV-2 virus appears to enact its pathogenic effects. We found no convincing evidence to justify starting or stopping currently prescribed drugs to influence outcomes of COVID-19 disease.

For peer review only

Strengths and limitations

- This review addresses a high priority patient and clinician concern
- Given the limited evidence on the subject, we included human and animal models both in vivo and in vitro for a comprehensive review
- This is the first systematic review specifically focussed on UK prescribed drugs that could alter ACE2 in COVID-19 disease
- The heterogeneity across study designs and models meant meta-analysis was not suitable
- Given the rapidly changing evidence as the pandemic progresses, it is possible that new studies have since been published.

For peer review only

INTRODUCTION

The coronavirus SARS-CoV-2 which causes the COVID-19 disease is a global public health emergency. It has been reported in 190 countries with 4,310,786 confirmed cases and 290,455 deaths as of 12th May 2020. Walker and colleagues from the World Health Organization Collaborating Centre for Infectious Disease Modelling predicted that in the absence of mitigation strategies, the virus would infect seven billion people and account for 40 million deaths this year alone.[1] Efforts to shield the elderly (60% reduction in social contacts) and interrupt transmission (40% reduction in social contacts for the wider population) have reduced this number but further deaths are still expected.[1] There is an urgent need for solutions. In the absence of a vaccination or effective treatment, there is growing interest in repurposing existing drugs for mitigation.

In particular, drugs affecting the Renin-Angiotensin System (RAS) have been highlighted as potential candidates for further investigation.[2,3] This is because the SARS-CoV-2 virus utilises Angiotensin-Converting Enzyme 2 (ACE2) receptors within the RAS for entry into lung alveolar epithelial cells.[4] ACE2 has previously been shown to correlate with susceptibility to the SARS-CoV-1 virus, and the spike (S) glycoprotein of this new virus binds to ACE2 with even higher affinity.[5,6] Theoretically, altered ACE2 activity could therefore lead to a greater susceptibility to SARS-CoV-2. It could also cause greater severity of the infection.[7] Previous studies suggest that dysregulation of ACE2 activity in the lungs could promote early neutrophil infiltration and subsequent uncontrolled activation of the RAS system.[8] In mice models, acute lung injury was observed in response to SARS-CoV-1 spike protein, so it is plausible that similar responses will be observed with SARS-CoV-2.[9]This is particularly problematic in organs containing high ACE2 such as the lungs as it may contribute to cytokine release syndrome (cytokine storm) and the subsequent respiratory failure that has been observed in those who have died from the disease.[7] Many prescribed drugs in common use are known to mediate effects through the RAS pathway. Over 45 million of these prescriptions were issued in the UK last year alone, and of these, 15 million were for Angiotensin Converting Enzyme Inhibitors (ACE-I) and Angiotensin Receptor Blockers (ARBs). Acting through the RAS pathway, these drugs may impact ACE2 regulation but their role in the COVID-19 pandemic is not clear. Given the number of people that are potentially on these drugs, it has caused substantial public concern and clinical uncertainty about continuation or cessation of prescribed medications during the pandemic. Accordingly, we reviewed all existing evidence on routinely prescribed UK drugs that might alter ACE2 regulation. Understanding the drug effects on ACE2 given its role in COVID-19 disease could help reassure clinicians and the public in these uncertain times, or direct research on drugs that might attenuate or exacerbate transmission.

METHODS

Our review was conducted in accordance with preferred reporting for systematic reviews and meta-analyses (PRISMA) guidelines and our protocol was submitted for open-access publication prior to commencing our study (in press). We have also made it available in pre-print through the medRxiv manuscript processing system.

Search Strategy

A systematic search in MEDLINE, EMBASE, CINAHL, the Cochrane Library, Web of Science was conducted from inception to the 1st April 2020. The search strategy is shown in Table 1 below. The reference lists of recent reviews and included studies were screened. We also spoke to topic experts and screened OpenGrey for additional texts. No language limits or study design filters were applied.

For peer review only

Table 1: Summary of search terms

Terms	Database	Number of articles
(ace2[Title/Abstract]) OR ace 2[Title/Abstract] Filters: English	Medline (PubMed)	1980
(ace2 or ace 2).ab. and English.lg.	Embase	2880
ace2 in Title Abstract Keyword OR ace-2 in Title Abstract Keyword - (Word variations have been searched)	Cochrane	35 trials and 1 review
(TS=(ace2 OR ace-2)) AND LANGUAGE: (English) AND DOCUMENT TYPES: (Article)	Web of science	1,931

Study selection, inclusion and exclusion criteria

The COVID-19 disease is still relative new and there is limited research on drug therapies specific to the virus. In the interest of being comprehensive about potential drugs acting through ACE2, we were as inclusive as possible within our study selection. We included both animal and human models (in vivo and in-vitro). Studies had to meet the following eligibility criteria: i) measures ACE2 levels, activity or gene expression, ii) includes a drug that is currently available on a UK prescription according to the British National Formulary, and iii) measures the effect of that drug against a placebo, control or sham group in an experimental design. Review articles were excluded but their reference lists were screened. Conference abstracts were included if sufficient detail could be elicited. We did not include studies in children under 18 years, or those examining drug effects in utero.

Data extraction

Four members of the team reviewed titles and abstracts for eligibility (AA, HDM, CW, SH). Full-text review, data extraction and quality assessment were carried out in duplicate using a piloted sheet. Any disagreement between authors was resolved by discussion. Data on the following study characteristics were extracted: i) drug class, ii) drug name, iii) duration of treatment, iii) effect on ACE2 level (upregulation, downregulation, no effect), iv) model (human/rat etc), v) site of ACE2 reception (lung, renal, cardiac etc;), vi) study design, vii) study population, viii) sample size and ix) country. Given the urgency of our research question during the current pandemic, we extracted information from only what was available to us in the published text.

Quality assessment

Our review includes both animal and human models therefore quality assessment was carried out separately for these studies. Human studies were evaluated using the Cochrane risk of bias tool which includes the following domains: random sequence generation, allocation concealment, blinding of participants and personnel, blinding of outcome assessment, incomplete outcome data, selective reporting, and other sources of bias.[10] Each domain was scored as low risk, unclear risk, or high risk of bias. We classified the overall risk of bias as low if all domains were at low risk of bias, as high if at least one domain was at high risk of bias, or as unclear if at least one domain was at unclear and no domain was at high risk of bias. Although this tool is specific to trials, all

included studies were experimental designs and we therefore felt it appropriate to use. The methodological quality of animal studies was assessed using the SYRCLE's risk of bias tool which is based on the Cochrane risk of bias tool.[11] SYRCLE's tool includes selection bias, performance bias, detection bias, attrition bias, reporting bias and other biases.

Data analysis

Owing to the mix of study designs and models, a meta-analysis was not appropriate. Narrative synthesis methods were used. We reviewed the meta-data by tabulating the studies according to our inclusion/exclusion criteria, human/animal model, drug classes and effects on ACE2. The consistency in the number of studies and direction of any effects were considered. Where inconsistencies were identified in the effect of a drug between studies, we looked at additional data such as methods, quality and outcome measurement for potential explanatory factors.

Patient and public involvement

It was not possible to involve patients or the public in the design or conduct of our work due to the rapid timelines, but we have invited PPI representatives to help us with drafting a lay summary and in the dissemination of our findings.

Results

We retrieved 6821 studies and screened 3,360 after removing duplicates. Following title and abstract screening, 233 studies were screened by full text. We included 112 studies in the final review. The flow of studies is shown in a PRISMA diagram in figure 1 including the reasons for study exclusion at each stage.

Study characteristics

Table 2 shows the characteristics of the included studies. These originated from 17 different countries with the most common being China (n= 36), the USA (n= 22) and Japan (n=18). There were 10 studies in humans (7 in-vitro and 3 in-vivo) (Table 3) and 102 in animal models (13 in-vitro and 89 in-vivo). Animal models included rats (n=94), mice (n=7) and canines (n=1). The sample sizes for in-vivo animal models ranged from 6 to 117. For in-vivo human studies (Table 3), sample sizes ranged from 8 to 375 but were not always reported. Participants were predominantly male and white with hypertension or diabetes, although the condition was not always stated. Most models examined ACE2 receptors in the heart or kidneys, only 5 of the 112 included studies reporting ACE2 levels in the lungs; these were all in animal models as shown in Table 2.

Effects of drugs

There were 21 different drug classes examined in the included studies as shown in Table 2. Table 3 tabulates only those that have been examined in human models. The mean drug exposure period ranged from 30 mins for in-vitro studies, to 15 weeks for in-vivo studies. The most common drug classes were ARBs (n= 55) and ACE-I (n= 22). Of the 55 studies that examined ARBs, 43 reported upregulation of ACE2 levels. Most of these studies were in rat models (n= 34) and examined cardiac ACE2 levels (n= 27). For ACE-I, 17 out of 22 studies reported upregulation of ACE2. These were also mainly in rat models (n= 16) and measured cardiac ACE2 levels (n= 14). Of the five studies that assessed statins, these were all within rat models; 3 reported upregulation of ACE2, 1 reported downregulation and 1 reported no effect. Similarly, oestrogens were examined in 5 studies; 3 reported upregulation, 1 reported downregulation and 1 reported no effects. For calcium

For peer review only

channel blockers; 2 out of the 3 studies reported upregulation of ACE2 levels and these were both in-vivo rodent models. The third study was an in-vitro human model that showed downregulation of ACE2 with a calcium channel blocker. There were 3 studies on aldosterone antagonists; all reporting increases in renal ACE2 levels within rat models.

Several diabetes drugs were evaluated and found to increase ACE2. For insulin, 6 out of 8 studies reported upregulation of ACE2 (in mice and rat models). For thiazolidinediones, 5 out of 7 studies reported upregulation (6 mice/rate models and 1 of cerebral human cells in-vitro). For GLP-1 agonists both included studies reported increases in ACE2. Similarly, for the one study examining DPP4 inhibitors, it also reported an increase in ACE2. The only study measuring the effect of SGLT2 inhibitors reported a decrease in ACE2.

Quality assessment

Figure 2 shows the risk of bias across the studies using the SYRCLE's risk of bias tool. In general, studies lacked blind allocation and outcome assessor blinding. They also frequently omitted information needed to make a thorough judgement on the risk of bias.

Discussion

To our knowledge, this is the most comprehensive review of drugs prescribed in the UK that could act on ACE2 receptors and thus potentially affect COVID-19 disease. The ACE2 receptor is reported to be an essential contributor to SARS-CoV-2 entry into the nasopharynx and lungs and the subsequent inflammation that leads to severe acute respiratory distress syndrome.[12,13] Our review examined drugs across human and animal models, and we found a number of studies reporting upregulation of ACE2 levels in response to ACE-I (n=22), ARBs (n=55), insulin (n=8), thiazolidinedione (n=7) aldosterone agonists (n=3), statins (n=5), oestrogens (n=5) calcium channel-blockers (n=3) GLP-1 agonists (n=2) and NSAIDs (n=2). However, these drugs were poorly studied in vivo within the lungs or nasopharynx of humans, where they are likely to matter most in influencing severity of outcomes of COVID-19 disease.

We observed that the most frequent drugs to upregulate ACE2 are also those prescribed in people with diabetes or cardiovascular disease. Mortality rates from COVID-19 have been high in this group.[12,14–16] Notably, these are also conditions with a high prevalence amongst Black, Asian and Minority Ethnic (BAME) groups who have had disproportionally high mortality rates from COVID-19 disease.[17] To date, much of this evidence has been limited to clinical commentaries or case reports. Larger cohorts are emerging but have not yet adequately considered a range of potential confounders including co-morbidities, age, sex, deprivation or household numbers which might be more important than prescribed medication in the spread, susceptibility and severity of the disease. For example, in a cohort of 191 people who were infected with the virus in Wuhan, 87% of those who died had coronary heart disease and 47% had diabetes.[18] These conditions are associated with an increased risk of death but were not considered as covariates in the analysis. Irrespective of ACE2, people with diabetes are more susceptible to worse infection as the low-grade chronic inflammation and hyperglycaemia associated with the condition results in impaired immune responses with lower IL-1, IL-6, TNF-a, and delayed mobilisation of immune cells in response to pathogens.[19] This comorbidity like many other confounders, are highly relevant when examining the risk of death with COVID-19 disease.

This lack of adequate adjustment for existing conditions is highlighted by Sommerstein and colleagues in their editorial on ACE-I and ARBs in COVID-19.[15] They also propose that existing

For peer review only

co-morbidities such as heart failure may be independently linked to SARS-CoV-2 transmission and severity, and the subsequent poor pulmonary outcomes that are observed in these patients. Indeed, in mice models, arterial hypertension, atrial fibrillation and type 2 diabetes have been shown to upregulate ACE2 levels irrespective of medications.[20,21] Moreover, ACE2 levels have been shown to be higher in men and with increasing age.[22] Most of the published data on deaths in COVID-19 disease report that males of increasing age are particularly susceptible to poor outcomes.[12,23]

Our review has also highlighted the variable ACE2 levels in different parts of the body with most of the existing literature focussing on renal and cardiac levels. Responses to drugs may vary depending on cell type and location. Although the lung ACE2 is important to COVID-19, it is unclear if overall COVID-19 mortality might be attenuated by cardiovascular ACE-2 activity levels. We also observed variations in ACE2 levels with drug exposure duration which was relatively short amongst included studies in our review. It is uncertain how dysregulation might continue after starting or stopping these medications. It is also unclear how the observed effects amongst included studies would translate in-vivo in humans and what the net effect on receptor access to the COVID-19 virus is; access to the receptor by the virus may be competitively inhibited by the presence of drugs which also attach to the receptor, so whether upregulation is the key factor in practice is unclear. This is particularly challenging to understand as we found a paucity of data demonstrating the effect of prescribed drugs on ACE2 in the lungs or nasopharynx, where the SARS-CoV-2 virus appears to enact its pathogenic effects. Our results therefore, do not provide convincing evidence on the role of any currently prescribed UK drugs acting through ACE2 regulation that could affect COVID-19 disease. Finally, we found a disproportionate number of studies reporting upregulation or 'positive effects' of drugs on ACE2, compared to studies reporting no effect or downregulation. This may reflect a publication bias that is well-established in the literature, especially amongst animal models. [24,25]

Strengths and limitations

We carried out a comprehensive and systematic search of the literature. To our knowledge, this is the first review on the subject. We did not include language restrictions but non-English language studies in the international literature might not have been indexed in the databases we searched. Given the rate of new publications on COVID-19, it is also possible that our search and results may not be up to date. Owing to the limited research on this novel virus, it was necessary to be as inclusive as possible and we therefore considered both animal and human models to look for any drugs acting through ACE2 with potential to affect COVID-19 outcomes. While this inclusive approach may offer insights, the heterogeneity across models makes it hard to interpret findings or translate them directly to patients. Although we were robust in our methodological approach to this review, we were also aware of the urgency to report our findings in the current pandemic. We therefore did not contact authors for more information about their studies beyond what was published. We observed frequent omission of information that would have allowed us to carry out a more detailed quality assessment. Had we pursued this information; the quality assessment of included papers may well have been higher.

Conclusion:

We reviewed the evidence on routinely prescribed drugs in the UK that could up or down-regulate ACE2 and thus potentially affect COVID-19 disease. Our review indicates that currently prescribed drugs have been poorly studied in-vivo within the lungs of humans. Until there is better evidence, we cannot recommend starting or stopping prescribed medications during the COVID-19 pandemic.

For peer review only

Acknowledgements: We would like to thank Prof Julia Hippisley-Cox for her early contributions to this project.

Competing Interests: ewsadxzAll authors have completed the ICMJE uniform disclosure form and confirm no financial relationships with any organisations that might have an interest in the submitted work in the previous three years, no other relationships or activities that could appear to have influenced the submitted work.

Funding: The Southampton, Cambridge and Oxford Primary Care Departments are members of the NIHR School for Primary Care Research and supported by NIHR Research funds. The University of Cambridge has received salary support in respect of SJG from the NHS in the East of England through the Clinical Academic Reserve. SJG is supported by an MRC Epidemiology Unit programme: MC_UU_12015/4. HDM is an NIHR Clinical Lecturer and supported by an NIHR SPCR grant for this work: SPCR2014-10043. The views expressed are those of the author(s) and not necessarily those of the NHS, the NIHR or the Department of Health and Social Care

Ethical Approval: Ethical approval is not required for a systematic review

Data sharing: No other data available

Author Contribution: HDM contributed to the design of the study, wrote the analysis plan, conducted the analysis, drafted and revised the paper. AA contributed to the design of the study, led the analysis, drafted and revised the paper. CW and SH contributed to the screening of studies, data extraction and revised the paper. NI revised the paper. SG and PL contributed to the design of the study and revised the paper. HDM is guarantor.

Declaration: The lead author affirms that the manuscript is an honest, accurate, and transparent account of the study being reported; that no important aspects of the study have been omitted. The opinions, results, and conclusions reported in this article are those of the authors and are independent from the funding sources.

Exclusive license: The Corresponding Author has the right to grant on behalf of all authors and does grant on behalf of all authors, a worldwide licence the Publishers and its licensees in perpetuity, in all forms, formats and media (whether known now or created in the future), to i) publish, reproduce, distribute, display and store the Contribution, ii) translate the Contribution into other languages, create adaptations, reprints, include within collections and create summaries, extracts and/or, abstracts of the Contribution and convert or allow conversion into any format including without limitation audio, iii) create any other derivative work(s) based in whole or part on the on the Contribution, iv) to exploit all subsidiary rights to exploit all subsidiary rights that currently exist or as may exist in the future in the Contribution, v) the inclusion of electronic links from the Contribution to third party material where-ever it may be located; and, vi) licence any third party to do any or all of the above.

References

1. Gt Walker P, Whittaker C, Watson O, et al. *The Global Impact of COVID-19 and Strategies for Mitigation and Suppression*.
2. Patel AB, Verma A. COVID-19 and Angiotensin-Converting Enzyme Inhibitors and Angiotensin Receptor Blockers. *JAMA*. March 2020. doi:10.1001/jama.2020.4812
3. Fang L, Karakiulakis G, Roth M. Are patients with hypertension and diabetes mellitus at increased risk for COVID-19 infection? 2020. doi:10.1016/S2213-2600(20)30116-8
4. Jia H. Pulmonary Angiotensin-Converting Enzyme 2 (ACE2) and Inflammatory Lung Disease. *SHOCK*. 2016;46(3):239-248. doi:10.1097/SHK.0000000000000633
5. Hofmann H, Geier M, Marzi A, et al. Susceptibility to SARS coronavirus S protein-driven infection correlates with expression of angiotensin converting enzyme 2 and infection can be blocked by soluble receptor. *Biochemical and Biophysical Research Communications*.

For peer review only - http://bmjopen.bmj.com/site/about/guidelines.xhtml

2004;319(4):1216-1221. doi:10.1016/j.bbrc.2004.05.114
6. Wrapp D, Wang N, Corbett KS, et al. Cryo-EM structure of the 2019-nCoV spike in the prefusion conformation. *Science*. 2020;367(6483):1260-1263. doi:10.1126/science.aax0902
7. Shi Y, Wang Y, Shao C, et al. COVID-19 infection: the perspectives on immune responses. *Cell death and differentiation*. March 2020:1-4. doi:10.1038/s41418-020-0530-3
8. Sodhi CP, Wohlford-Lenane C, Yamaguchi Y, et al. Attenuation of pulmonary ACE2 activity impairs inactivation of des-arg9 bradykinin/BKB1R axis and facilitates LPS-induced neutrophil infiltration. *American Journal of Physiology - Lung Cellular and Molecular Physiology*. 2018;314(1):L17-L31. doi:10.1152/ajplung.00498.2016
9. Kuba K, Imai Y, Rao S, et al. A crucial role of angiotensin converting enzyme 2 (ACE2) in SARS coronavirus-induced lung injury. *Nature Medicine*. 2005;11(8):875-879. doi:10.1038/nm1267
10. Higgins JPT, Altman DG, Gøtzsche PC, et al. The Cochrane Collaboration's tool for assessing risk of bias in randomised trials. *BMJ (Online)*. 2011;343(7829). doi:10.1136/bmj.d5928
11. Hooijmans CR, Rovers MM, De Vries RBM, Leenaars M, Ritskes-Hoitinga M, Langendam MW. SYRCLE's risk of bias tool for animal studies. *BMC Medical Research Methodology*. 2014;14(1):43. doi:10.1186/1471-2288-14-43
12. Wu C, Chen X, Cai Y, et al. Risk Factors Associated With Acute Respiratory Distress Syndrome and Death in Patients With Coronavirus Disease 2019 Pneumonia in Wuhan, China. *JAMA Internal Medicine*. March 2020. doi:10.1001/jamainternmed.2020.0994
13. Gorbalenya AE, Baker SC, Baric RS, et al. Severe acute respiratory syndrome-related coronavirus: The species and its viruses-a statement of the Coronavirus Study Group. doi:10.1101/2020.02.07.937862
14. OpenSAFELY Collaborative T, Williamson E, Walker AJ, et al. OpenSAFELY: factors associated with COVID-19-related hospital death in the linked electronic health records of 17 million adult NHS patients. doi:10.1101/2020.05.06.20092999
15. Sommerstein R, Kochen MM, Messerli FH, Gräni C. Coronavirus Disease 2019 (COVID-19): Do Angiotensin-Converting Enzyme Inhibitors/Angiotensin Receptor Blockers Have a Biphasic Effect? *Journal of the American Heart Association*. 2020;9(7). doi:10.1161/jaha.120.016509
16. Chen Y, Gong X, Wang L, Guo J. Effects of hypertension, diabetes and coronary heart disease on COVID-19 diseases severity: a systematic review and meta-analysis. *medRxiv*. March 2020:2020.03.25.20043133. doi:10.1101/2020.03.25.20043133
17. BAME COVID-19 DEATHS - What do we know? Rapid Data & Evidence Review - CEBM. https://www.cebm.net/covid-19/bame-covid-19-deaths-what-do-we-know-rapid-data-evidence-review/. Accessed May 6, 2020.
18. Zhou F, Yu T, Du R, et al. Clinical course and risk factors for mortality of adult inpatients with COVID-19 in Wuhan, China: a retrospective cohort study. *The Lancet*. 2020;395(10229):1054-1062. doi:10.1016/S0140-6736(20)30566-3
19. Iacobellis G. COVID-19 and diabetes: Can DPP4 inhibition play a role? 2020. doi:10.1016/j.diabres.2020.108125
20. Úri K, Fagyas M, Siket IM, et al. New perspectives in the renin-angiotensin-aldosterone system (RAAS) IV: Circulating ACE2 as a biomarker of systolic dysfunction in human hypertension and heart failure. *PLoS ONE*. 2014;9(4). doi:10.1371/journal.pone.0087845
21. Angiotensin-converting enzyme-2 overexpression improves atrial remodeling and function in a canine model of atrial fibrillation. *J Am Heart Assoc*. 4:1-9.
22. Fernández-Atucha A, Izagirre A, Fraile-Bermúdez AB, et al. Sex differences in the aging pattern of renin-angiotensin system serum peptidases. *Biology of Sex Differences*. 2017;8(1):5. doi:10.1186/s13293-017-0128-8
23. Deng S-Q, Peng H-J. Characteristics of and Public Health Responses to the Coronavirus Disease 2019 Outbreak in China. *Journal of Clinical Medicine*. 2020;9(2):575. doi:10.3390/jcm9020575
24. Murad MH, Chu H, Lin L, Wang Z. The effect of publication bias magnitude and direction on the certainty in evidence. *BMJ evidence-based medicine*. 2018;23(3):84-86. doi:10.1136/bmjebm-2018-110891
25. Conradi U, Joffe AR. Publication bias in animal research presented at the 2008 Society of Critical Care Medicine Conference. *BMC Research Notes*. 2017;10(1):262. doi:10.1186/s13104-017-2574-0
26. Zhen Y, Xin Y, Wenping J. GW24-e1144 Effects of enalapril on the expression of cardiac angiotensin-converting enzyme and angiotensin-converting enzyme 2 in spontaneously hypertensive rats. *Heart*. 2013;99(Suppl 3):A190.1-A190. doi:10.1136/heartjnl-2013-

For peer review only

304613.529
27. Azis NA, Agarwal R, Ismail NM, et al. Blood pressure lowering effect of Ficus deltoidea var kunstleri in spontaneously hypertensive rats: possible involvement of renin-angiotensin-aldosterone system, endothelial function and anti-oxidant system. *Molecular biology reports*. 2019;46(3):2841-2849. doi:10.1007/s11033-019-04730-w
28. Awwad ZM, El-Ganainy SO, ElMallah AI, et al. Telmisartan and captopril ameliorate pregabalin-induced heart failure in rats. *Toxicology*. 2019;428:152310. doi:10.1016/j.tox.2019.152310
29. Graus-Nunes F, Santos F de O, Marinho T de S, Miranda CS, Barbosa-da-Silva S, Souza-Mello V. Beneficial effects of losartan or telmisartan on the local hepatic renin-angiotensin system to counter obesity in an experimental model. *World journal of hepatology*. 2019;11(4):359-369. doi:10.4254/wjh.v11.i4.359
30. Hao X-Q, Zhang S-Y, Cheng X-C, et al. Imidapril inhibits right ventricular remodeling induced by low ambient temperature in broiler chickens. *Poultry Science*. 2013;92(6):1492-1497. doi:http://dx.doi.org/10.3382/ps.2012-02671
31. Jessup JA, Gallagher PE, Averill DB, et al. Effect of angiotensin II blockade on a new congenic model of hypertension derived from transgenic Ren-2 rats. *American journal of physiology Heart and circulatory physiology*. 2006;291(5):H2166-72. doi:10.1152/ajpheart.00061.2006
32. Huang M, Li X, Meng Y, et al. Upregulation of angiotensin-converting enzyme (ACE) 2 in hepatic fibrosis by ACE inhibitors. *Clinical and Experimental Pharmacology and Physiology*. 2010;37(1):e1-e6. doi:http://dx.doi.org/10.1111/j.1440-1681.2009.05302.x
33. Wang G, Zhang Q, Yuan W, Wu J, Li C. Enalapril protects against myocardial ischemia/reperfusion injury in a swine model of cardiac arrest and resuscitation. *International journal of molecular medicine*. 2016;38(5):1463-1473. doi:10.3892/ijmm.2016.2737
34. Wang Y, Li C, Ouyang Y, et al. Cardioprotective Effects of Qishenyiqi Mediated by Angiotensin II Type 1 Receptor Blockade and Enhancing Angiotensin-Converting Enzyme 2. *Evidence-based Complementary and Alternative Medicine*. 2012;2012:978127. doi:http://dx.doi.org/10.1155/2012/978127
35. Burchill L, Velkoska E, Dean RG, et al. Acute kidney injury in the rat causes cardiac remodelling and increases angiotensin-converting enzyme 2 expression. *Experimental physiology*. 2008;93(5):622-630. doi:10.1113/expphysiol.2007.040386
36. Zhang Y, Wang B, Wu J, et al. Role of ACE inhibitor and AT1 blockade on cardiac ACE and ACE2 expression in mice with unilateral ureteral ligation. *International Journal of Cardiology*. 2011;152(SUPPL. 1):S97. doi:10.1016/j.ijcard.2011.08.788
37. Zhang Y, Li B, Ma L, Wu J. GW25-e0791 Role of AT1 blockade on cardiac ACE2 and mas expression in hypertensive rats. *Journal of the American College of Cardiology*. 2014;64(16):C179. doi:10.1016/j.jacc.2014.06.825
38. Ocaranza MP, Godoy I, Jalil JE, et al. Enalapril attenuates downregulation of angiotensin-converting enzyme 2 in the late phase of ventricular dysfunction in myocardial infarcted rat. *Hypertension*. 2006;48(4):572-578. doi:10.1161/01.HYP.0000237862.94083.45
39. Chen L, Yu M ZC et al., M. Alterations of urinary angiotensin converting enzyme and ACE2 before and after perindopril medication in patients with diabetic kidney disease. *Diabetes/Metabolism Research and Reviews*. 2015;31(Supplement 1):51-83. doi:10.1002/dmrr.2728
40. Fagyas M, Uri K, Manyine Siket I EI and PZ. Tuesday, 30 August 2011. *European Heart Journal*. 2011;32(Supplement 1):633-933. doi:10.1093/eurheartj/ehr324
41. Xiao H-L, Li C-S, Zhao L-X, et al. Captopril improves postresuscitation hemodynamics protective against pulmonary embolism by activating the ACE2/Ang-(1-7)/Mas axis. *Naunyn-Schmiedeberg's archives of pharmacology*. 2016;389(11):1159-1169. doi:10.1007/s00210-016-1278-7
42. Wu J, Chen T, Zhang Y. GW26-e0735 Cardiac ace2/mas expression and cardiac remodeling in hypertensive rats. *Journal of the American College of Cardiology*. 2015;66(16):C76. doi:10.1016/j.jacc.2015.06.304
43. P. F, Z. W, H. L, et al. Electroacupuncture Improved Chronic Cerebral Hypoperfusion-Induced Anxiety-Like Behavior and Memory Impairments in Spontaneously Hypertensive Rats by Downregulating the ACE/Ang II/AT1R Axis and Upregulating the ACE2/Ang-(1-7)/MasR Axis. *Neural plasticity*. 2020;2020:9076042. doi:http://dx.doi.org/10.1155/2020/9076042
44. Abuohashish HM, Ahmed MM, Sabry D, Khattab MM, Al-Rejaie SS. ACE-2/Ang1-7/Mas cascade mediates ACE inhibitor, captopril, protective effects in estrogen-deficient osteoporotic rats. *Biomedicine & pharmacotherapy = Biomedecine & pharmacotherapie*. 2017;92:58-68.

doi:10.1016/j.biopha.2017.05.062
45. Flores-Monroy J, Martinez-Aguilar L, Ferrario C, Valencia-Hernandez I. PM097 Effects of a Novel Angiotensin-Converting Enzyme Inhibitor After Myocardial Infarction. *Global Heart*. 2016;11(2):e87. doi:10.1016/j.gheart.2016.03.299
46. Ibarra-Lara L, Del Valle-Mondragon L, Soria-Castro E, et al. Peroxisome proliferator-activated receptor-alpha stimulation by clofibrate favors an antioxidant and vasodilator environment in a stressed left ventricle. *Pharmacological reports : PR*. 2016;68(4):692-702. doi:10.1016/j.pharep.2016.03.002
47. Badae NM, El Naggar AS, El Sayed SM, et al. Is the cardioprotective effect of the ACE2 activator diminazene aceturate more potent than the ACE inhibitor enalapril on acute myocardial infarction in rats? *Canadian journal of physiology and pharmacology*. 2019;97(7):1-9. doi:http://dx.doi.org/10.1139/cjpp-2019-0078
48. Bernardi S, Toffoli B, Zennaro C, et al. Aldosterone effects on glomerular structure and function. *Journal of the renin-angiotensin-aldosterone system : JRAAS*. 2015;16(4):730-738. doi:10.1177/1470320315595568
49. Dong D, Fan T-T, Ji Y-S, et al. Spironolactone alleviates diabetic nephropathy through promoting autophagy in podocytes. *International urology and nephrology*. 2019;51(4):755-764. doi:http://dx.doi.org/10.1007/s11255-019-02074-9
50. Kong E liang, Zhang J min, An N, Tao Y, Yu W feng, Wu F xiang. Spironolactone rescues renal dysfunction in obstructive jaundice rats by upregulating ACE2 expression. *Journal of Cell Communication and Signaling*. 2019;13(1):17-26. doi:10.1007/s12079-018-0466-2
51. Abe M, Oikawa O, Okada K, et al. Urinary angiotensin-converting enzyme 2 increases in diabetic nephropathy by angiotensin II type 1 receptor blocker olmesartan. *Journal of the renin-angiotensin-aldosterone system : JRAAS*. 2015;16(1):159-164. doi:http://dx.doi.org/10.1177/1470320314551443
52. de Araujo AA, Araujo L de S, Carvalho CA, et al. Protective effect of angiotensin II receptor blocker against oxidative stress and inflammation in an oral mucositis experimental model. *Journal of Oral Pathology and Medicine*. 2018;47(10):972-984. doi:10.1111/jop.12775
53. Tanno T, Tomita H, Narita I, et al. Olmesartan Inhibits Cardiac Hypertrophy in Mice Overexpressing Renin Independently of Blood Pressure: Its Beneficial Effects on ACE2/Ang(1-7)/Mas Axis and NADPH Oxidase Expression. *Journal of cardiovascular pharmacology*. 2016;67(6):503-509. doi:10.1097/FJC.0000000000000374
54. Ishibashi Y, Matsui T, Yamagishi S. Olmesartan blocks advanced glycation end products-induced vcam-1 gene expression in mesangial cells by restoring angiotensin-converting enzyme 2 level. *Hormone and Metabolic Research*. 2014;46(6):379-383. doi:10.1055/s-0033-1361114
55. Wang W, Song A, Zeng Y, et al. Telmisartan protects chronic intermittent hypoxic mice via modulating cardiac renin-angiotensin system activity. *BMC Cardiovascular Disorders*. 2018;18(1). doi:10.1186/s12872-018-0875-4
56. Malek V, Sharma N, Sankrityayan H, Gaikwad AB. Concurrent neprilysin inhibition and renin-angiotensin system modulations prevented diabetic nephropathy. *Life Sciences*. 2019;221:159-167. doi:10.1016/j.lfs.2019.02.027
57. Hiroi N, Yoshihara A SM et al. Effects of olmesartan to the ACE2/Ang-(1-7)/Mas receptor endocrine society axis on essential hypertension patients. *Endocrine Reviews*. 2014;35(SUPPL. 3).
58. Li Y qin, Wu J juan, Gao D feng, Fan Y mei, Zhang Y, Qin X jin. The impact of telmisartan on angiotensin converting enzyme 2 mRNA expression in monocyte-derived macrophages of diabetic hypertensive patients]. *Zhonghua nei ke za zhi*. 2013;52(1):26-29.
59. Wang X, Ye Y, Gong H, et al. The effects of different angiotensin II type 1 receptor blockers on the regulation of the ACE-AngII-AT1 and ACE2-Ang(1-7)-Mas axes in pressure overload-induced cardiac remodeling in male mice. *Journal of molecular and cellular cardiology*. 2016;97:180-190. doi:10.1016/j.yjmcc.2016.05.012
60. Soler MJ, Ye M, Wysocki J, et al. Localization of ACE2 in the renal vasculature: Amplification by angiotensin II type 1 receptor blockade using telmisartan. *American journal of physiology Renal physiology*. 2009;296(2):F398-F405. doi:10.1152/ajprenal.90488.2008
61. Senador D, Key M, Brosnihan KB, et al. Cardiovascular interactions between losartan and fructose in mice. *Journal of cardiovascular pharmacology and therapeutics*. 2010;15(1):68-77. doi:http://dx.doi.org/10.1177/1074248409351409
62. Abdelkader NF, Abd El-Latif AM, Khattab MM. Telmisartan/17beta-estradiol mitigated cognitive deficit in an ovariectomized rat model of Alzheimer's disease: Modulation of ACE1/ACE2 and

For peer review only

AT1/AT2 ratio. *Life sciences*. 2020;245:117388. doi:10.1016/j.lfs.2020.117388
63. Li Y, Cai S, Wang Q, et al. Valsartan attenuates intimal hyperplasia in balloon-injured rat aortic arteries through modulating the angiotensin-converting enzyme 2-angiotensin-(1-7)-Mas receptor axis. *Archives of biochemistry and biophysics*. 2016;598:11-17. doi:http://dx.doi.org/10.1016/j.abb.2016.03.028
64. Zhang Y, Li B, Wang B, Zhang J, Wu J, Morgan T. Alteration of cardiac ACE2/Mas expression and cardiac remodelling in rats with aortic constriction. *The Chinese journal of physiology*. 2014;57(6):335-342. doi:10.4077/CJP.2014.BAD268
65. Ji K, Minakawa M, Fukui K, Suzuki Y, Fukuda I. Olmesartan improves left ventricular function in pressure-overload hypertrophied rat heart by blocking angiotensin II receptor with synergic effects of upregulation of angiotensin converting enzyme 2. *Therapeutic advances in cardiovascular disease*. 2009;3(2):103-111. doi:10.1177/1753944708098691
66. Sabry MM, Mahmoud MM, Shoukry HS, Rashed L, Kamar SS, Ahmed MM. Interactive effects of apelin, renin-angiotensin system and nitric oxide in treatment of obesity-induced type 2 diabetes mellitus in male albino rats. *Archives of physiology and biochemistry*. 2019;125(3):244-254. doi:10.1080/13813455.2018.1453521
67. Li C-M, Zhang X-H, Zhang Y-Y, Yu H-Z. The effect of Irbesartan on myocardial interstitial fibrosis in diabetic rats. *AFRICAN JOURNAL OF PHARMACY AND PHARMACOLOGY*. 2011;5(11):1360-1364. doi:10.5897/AJPP11.177
68. Abdel-Fattah MM, Messiha BAS, Mansour AM. Modulation of brain ACE and ACE2 may be a promising protective strategy against cerebral ischemia/reperfusion injury: an experimental trial in rats. *Naunyn-Schmiedeberg's Archives of Pharmacology*. 2018;391(9):1003-1020. doi:10.1007/s00210-018-1523-3
69. Arumugam S, Thandavarayan RA, Palaniyandi SS, et al. Candesartan cilexetil protects from cardiac myosin induced cardiotoxicity via reduction of endoplasmic reticulum stress and apoptosis in rats: involvement of ACE2-Ang (1-7)-mas axis. *Toxicology*. 2012;291(1-3):139-145. doi:10.1016/j.tox.2011.11.008
70. Kidoguchi S, Sugano N, Takane K, et al. Azilsartan causes natriuresis due to its sympatholytic action in kidney disease. *Hypertension research : official journal of the Japanese Society of Hypertension*. 2019;42(10):1507-1517. doi:10.1038/s41440-019-0271-1
71. Liang B, Li Y, Han Z, et al. ACE2-Ang (1-7) axis is induced in pressure overloaded rat model. *International journal of clinical and experimental pathology*. 2015;8(2):1443-1450.
72. Sukumaran V, Veeraveedu PT, Gurusamy N, et al. Telmisartan acts through the modulation of ACE-2/ANG 1-7/mas receptor in rats with dilated cardiomyopathy induced by experimental autoimmune myocarditis. *Life sciences*. 2012;90(7-8):289-300. doi:10.1016/j.lfs.2011.11.018
73. Yang D, Lin Z, Ni Y, Hou L, Yang D. A1059 Telmisartan upregulates ACE2-Ang-(1–7)-Mas axis and affects retinal vessel endothelial cell apoptosis. *Journal of Hypertension*. 2018;36(Supplement 3):e11. doi:10.1097/01.hjh.0000548029.92325.e5
74. Takai S, Jin D, Aritomi S, Niinuma K, Miyazaki M. Powerful vascular protection by combining cilnidipine with valsartan in stroke-prone, spontaneously hypertensive rats. *Hypertension research : official journal of the Japanese Society of Hypertension*. 2013;36(4):342-348. doi:10.1038/hr.2012.187
75. Zhong J-C, Ye J-Y, Jin H-Y, et al. Telmisartan attenuates aortic hypertrophy in hypertensive rats by the modulation of ACE2 and profilin-1 expression. *Regulatory peptides*. 2011;166(1-3):90-97. doi:10.1016/j.regpep.2010.09.005
76. Zhao Y, Ma R, Yu X, Li N, Zhao X, Yu J. AHU377+Valsartan (LCZ696) Modulates Renin-Angiotensin System (RAS) in the Cardiac of Female Spontaneously Hypertensive Rats Compared With Valsartan. *Journal of cardiovascular pharmacology and therapeutics*. 2019;24(5):450-459. doi:10.1177/1074248419838503
77. Agata J, Ura N, Yoshida H, et al. Olmesartan is an angiotensin II receptor blocker with an inhibitory effect on angiotensin-converting enzyme. *Hypertension research : official journal of the Japanese Society of Hypertension*. 2006;29(11):865-874. doi:10.1291/hypres.29.865
78. Igase M, Kohara K, Nagai T, Miki T, Ferrario CM. Increased expression of angiotensin converting enzyme 2 in conjunction with reduction of neointima by angiotensin II type 1 receptor blockade. *Hypertension research : official journal of the Japanese Society of Hypertension*. 2008;31(3):553-559. doi:10.1291/hypres.31.553
79. Igase M, Strawn WB, Gallagher PE, Geary RL, Ferrario CM. Angiotensin II AT1 receptors regulate ACE2 and angiotensin-(1-7) expression in the aorta of spontaneously hypertensive rats. *American journal of physiology Heart and circulatory physiology*. 2005;289(3):H1013-9. doi:10.1152/ajpheart.00068.2005

For peer review only

80. Wosten-van Asperen RM, Lutter R, Specht PA, et al. Acute respiratory distress syndrome leads to reduced ratio of ACE/ACE2 activities and is prevented by angiotensin-(1-7) or an angiotensin II receptor antagonist. *The Journal of pathology*. 2011;225(4):618-627. doi:10.1002/path.2987
81. Ohshima K, Mogi M, Nakaoka H, et al. Possible role of angiotensin-converting enzyme 2 and activation of angiotensin II type 2 receptor by angiotensin-(1-7) in improvement of vascular remodeling by angiotensin II type 1 receptor blockade. *Hypertension (Dallas, Tex : 1979)*. 2014;63(3):e53-9. doi:10.1161/HYPERTENSIONAHA.113.02426
82. Gallagher PE, Ferrario CM, Tallant EA. Regulation of ACE2 in cardiac myocytes and fibroblasts. *American journal of physiology Heart and circulatory physiology*. 2008;295(6):H2373-9. doi:10.1152/ajpheart.00426.2008
83. Feng P, Wu Z, Liu H, et al. Electroacupuncture Improved Chronic Cerebral Hypoperfusion-Induced Anxiety-Like Behavior and Memory Impairments in Spontaneously Hypertensive Rats by Downregulating the ACE/Ang II/AT1R Axis and Upregulating the ACE2/Ang-(1-7)/MasR Axis. *Neural plasticity*. 2020;2020:9076042. doi:10.1155/2020/9076042
84. Song R, Preston G, Yosypiv I V. Ontogeny of angiotensin-converting enzyme 2. *Pediatric research*. 2012;71(1):13-19. doi:10.1038/pr.2011.7
85. Lezama-Martinez D, Flores-Monroy J, Fonseca-Coronado S, Hernandez-Campos ME, Valencia-Hernandez I, Martinez-Aguilar L. Combined antihypertensive therapies that increase expression of cardioprotective biomarkers associated with the renin-angiotensin and kallikrein-kinin system. *Journal of Cardiovascular Pharmacology*. 2018;72(6):291-295. doi:10.1097/FJC.0000000000000629
86. Shimada K, Kitazato K KT et al. International Stroke Conference and Nursing Symposium Poster Presentations. *Stroke*. 2011;42(3):e149. doi:10.1161/str.0b013e3182074d9b
87. Ishiyama Y, Gallagher PE, Averill DB, Tallant EA, Brosnihan KB, Ferrario CM. Upregulation of angiotensin-converting enzyme 2 after myocardial infarction by blockade of angiotensin II receptors. *Hypertension (Dallas, Tex : 1979)*. 2004;43(5):970-976. doi:10.1161/01.HYP.0000124667.34652.1a
88. Guo J, Lu W, Wang X, et al. Protective effects of telmisartan in a rat model of pulmonary arterial hypertension. *Experimental and Clinical Cardiology*. 2014;20(1):2429-2437.
89. Yisireyili M, Uchida Y, Yamamoto K, et al. Angiotensin receptor blocker irbesartan reduces stress-induced intestinal inflammation via AT1a signaling and ACE2-dependent mechanism in mice. *Brain, behavior, and immunity*. 2018;69:167-179. doi:10.1016/j.bbi.2017.11.010
90. Suh SH, Choi HS, Kim CS, et al. Olmesartan Attenuates Kidney Fibrosis in a Murine Model of Alport Syndrome by Suppressing Tubular Expression of TGFbeta. *International journal of molecular sciences*. 2019;20(15). doi:10.3390/ijms20153843
91. Iwanami j, Mogi M TK et al. Role of angiotensin converting enzyme 2/angiotensin (1-7)/Mas axis in the hypotensive effect of azilsartan. *Hypertension*. 2013;62(3 MeetingAbstracts).
92. Puneet A, Manikant S, Singh SJ. Sexual dysfunction in indian patients undergoing maintenance haemodialysis; a cross sectional study. *Nephrology*. 2014;19(SUPPL. 2):182-183. doi:10.1111/nep.12237
93. Ichikawa H, Narita I, Narita M, et al. Blood pressure-independent effect of olmesartan on albuminuria in mice overexpressing renin: Its beneficial role in the ACE2/ang (1-7)/mas axis and NADPH oxidase expression. *International Heart Journal*. 2018;59(6):1445-1453. doi:10.1536/ihj.17-582
94. Nakaoka H, Mogi M, Kan-no H, et al. Effect of Olmesartan on Glucose Metabolism Involving Angiotensin Converting Enzyme 2. *Immunology, Endocrine & Metabolic Agents in Medicinal Chemistry*. 2017;17(2):105-114. doi:10.2174/1871522217666171019150511
95. Varagic J, Ahmad S, Voncannon JL, et al. Predominance of AT1 blockade over mas-mediated angiotensin-(1-7) mechanisms in the regulation of blood pressure and renin-angiotensin system in mRen2.Lewis rats. *American Journal of Hypertension*. 2013;26(5):583-590. doi:10.1093/ajh/hps090
96. Zhang YH, Hao QQ, Wang XY, et al. ACE2 activity was increased in atherosclerotic plaque by losartan: Possible relation to anti-atherosclerosis. *Journal of the renin-angiotensin-aldosterone system : JRAAS*. 2015;16(2):292-300. doi:10.1177/1470320314542829
97. Varagic J, Ahmad S, Voncannon JL, et al. Nebivolol reduces cardiac angiotensin II, associated oxidative stress and fibrosis but not arterial pressure in salt-loaded spontaneously hypertensive rats. *Journal of hypertension*. 2012;30(9):1766-1774. doi:10.1097/HJH.0b013e328356766f
98. Iizuka K, Kusunoki A, Machida T, Hirafuji M. Angiotensin II reduces membranous angiotensin-

For peer review only

converting enzyme 2 in pressurized human aortic endothelial cells. *Journal of the renin-angiotensin-aldosterone system : JRAAS*. 2009;10(4):210-215. doi:10.1177/1470320309343710
99. Onat E. Effects of rosuvastatin and amlodipine on reninangiotensin system of kidney in NOS inhibition and salt diet induced hypertension. *Journal of Cellular Neuroscience and Oxidative Stress*. 2018;10(2):693-694.
100. Zhang L-H, Pang X-F, Bai F, et al. Preservation of Glucagon-Like Peptide-1 Level Attenuates Angiotensin II-Induced Tissue Fibrosis by Altering AT1/AT 2 Receptor Expression and Angiotensin-Converting Enzyme 2 Activity in Rat Heart. *Cardiovascular drugs and therapy*. 2015;29(3):243-255. doi:10.1007/s10557-015-6592-7
101. Awwad ZM, El-Ganainy SO, ElMallah AI, Khedr SM, Khattab MM, El-Khatib AS. Assessment of Pregabalin-Induced Cardiotoxicity in Rats: Mechanistic Role of Angiotensin 1-7. *Cardiovascular toxicology*. November 2019. doi:10.1007/s12012-019-09553-6
102. Romani-Perez M, Outeirino-Iglesias V, Moya CM, et al. Activation of the GLP-1 Receptor by Liraglutide Increases ACE2 Expression, Reversing Right Ventricle Hypertrophy, and Improving the Production of SP-A and SP-B in the Lungs of Type 1 Diabetes Rats. *Endocrinology*. 2015;156(10):3559-3569. doi:10.1210/en.2014-1685
103. Riera M, Marquez E, Clotet S, et al. Effect of insulin on ACE2 activity and kidney function in the non-obese diabetic mouse. *PloS one*. 2014;9(1):e84683. doi:10.1371/journal.pone.0084683
104. Marquez E, Riera M, Pascual J, Soler MJ. Albumin inhibits the insulin-mediated ACE2 increase in cultured podocytes. *American journal of physiology Renal physiology*. 2014;306(11):F1327-34. doi:10.1152/ajprenal.00594.2013
105. Shin YH, Min JJ, Lee J-H, et al. The effect of fluvastatin on cardiac fibrosis and angiotensin-converting enzyme-2 expression in glucose-controlled diabetic rat hearts. *Heart and vessels*. 2017;32(5):618-627. doi:10.1007/s00380-016-0936-5
106. Salem ES, Chodavarapu H, Morris M, Elased KM. Insulin normalizes angiotensin converting enzyme 2 (ACE2) and attenuates albuminuria in type 1 diabetic Akita mice. *Hypertension*. 2012;60(3 Meeting Abstracts).
107. Salem E and Somineni HK. Acute and Chronic Complications. *Diabetes*. 2013;62(Supplement_1):A588-A621. doi:10.2337/db13-2297-2440
108. Salem ESB, Grobe N, Elased KM. Insulin treatment attenuates renal ADAM17 and ACE2 shedding in diabetic Akita mice. *American journal of physiology Renal physiology*. 2014;306(6):F629-39. doi:10.1152/ajprenal.00516.2013
109. Blanco-Gozalo V, Blazquez-Medela A, Garcia-Sanchez O, et al. Diabetes - experimental models. *Nephrology Dialysis Transplantation*. 2013;28(suppl 1):i374-i384. doi:10.1093/ndt/gft137
110. Min JJ, Shin B-S, Lee J-H, et al. Effects of pravastatin on type 1 diabetic rat heart with or without blood glycemic control. *Journal of Diabetes Research*. 2018;2018:1067853. doi:http://dx.doi.org/10.1155/2018/1067853
111. Gupta RC, Want G RS et al. Long-term therapy with ivabradine increases ace-2 activity in left ventricular myocardium of dogs with chronic heart failure. *Circulation Research*. 2012;111(4 MeetingAbstract).
112. Weili Q, Cheng W, Fan Z, et al. GW25-e4430 Ibuprofen Attenuates Cardiac Fibrosis via Restoring the Imbalance of ACE and ACE2 in Diabetic Rat. *Journal of the American College of Cardiology*. 2014;64(16):C62. doi:10.1016/j.jacc.2014.06.297
113. Qiao W, Wang C, Chen B, et al. Ibuprofen attenuates cardiac fibrosis in streptozotocin-induced diabetic rats. *Cardiology*. 2015;131(2):97-106. doi:10.1159/000375362
114. Bukowska A, Spiller L, Wolke C, et al. Protective regulation of the ACE2/ACE gene expression by estrogen in human atrial tissue from elderly men. *Experimental Biology and Medicine*. 2017;242(14):1412-1423. doi:10.1177/1535370217718808
115. Hermenegildo C, Mompeon A, Perez-Cremades D, Vidal-Gomez X, Gironacci MM, Novella S. Estradiol increaseses endothelial angiotensin-(1-7) production through esterogen reception alpha. *Journal of Hypertension*. 2016;34(Supplement 2):e75. doi:10.1097/01.hjh.0000491530.65088.bb
116. Mompeon A, Lazaro-Franco M, Bueno-Beti C, et al. Estradiol, acting through ERalpha, induces endothelial non-classic renin-angiotensin system increasing angiotensin 1-7 production. *Molecular and Cellular Endocrinology*. 2016;422:1-8. doi:http://dx.doi.org/10.1016/j.mce.2015.11.004
117. Shenoy V, Grobe JL, Qi Y, et al. 17beta-Estradiol modulates local cardiac renin-angiotensin

For peer review only

system to prevent cardiac remodeling in the DOCA-salt model of hypertension in rats. *Peptides*. 2009;30(12):2309-2315. doi:10.1016/j.peptides.2009.09.005
118. Wang H, Zhao Z JJ et al. Estrogen replacement downregulates cardiac angiotensin ii in an ace-independent manner in oophorectomized mRen2.Lewis rats. *Circulation Research*. 2013;113(4 MeetingAbstracts).
119. Wang G, Zhang Q, Yuan W, et al. Sildenafil protects against myocardial ischemia-reperfusion injury following cardiac arrest in a porcine model: Possible role of the renin-angiotensin system. *International Journal of Molecular Sciences*. 2015;16(11):27015-27031. doi:http://dx.doi.org/10.3390/ijms161126010
120. Thanekar U, Gill RK, Dhakal S, Hosawi A, Elased KM. Abstract P2044: Renoprotective Effects of Canagliflozin in Db/db Diabetic Mice. *Hypertension*. 2019;74(Suppl_1). doi:10.1161/hyp.74.suppl_1.p2044
121. Li Y-H, Wang Q-X, Zhou J-W, et al. Effects of rosuvastatin on expression of angiotensin-converting enzyme 2 after vascular balloon injury in rats. *Journal of geriatric cardiology : JGC*. 2013;10(2):151-158. doi:10.3969/j.issn.1671-5411.2013.02.009
122. Suski M, Gębska A, Olszanecki R, et al. Influence of atorvastatin on angiotensin i metabolism in resting and TNF-α -activated rat vascular smooth muscle cells. *JRAAS - Journal of the Renin-Angiotensin-Aldosterone System*. 2014;15(4):378-383. doi:10.1177/1470320313475907
123. Aguilar C, Ventura F, Rodríguez-Delfín L. Atorvastatin induced increase in homologous angiotensin I converting enzyme (ACE2) mRNA Is associated to decreased fibrosis and decreased left ventricular hypertrophy in a rat model of diabetic cardiomyopathy. *Revista Peruana de Medicina Experimental y Salud Publica*. 2011;28(2):264-272. doi:10.1590/S1726-46342011000200013
124. Jessup JA, Brosnihan KB, Gallagher PE, Chappell MC, Ferrario CM. Differential effect of low dose thiazides on the Renin Angiotensin system in genetically hypertensive and normotensive rats. *Journal of the American Society of Hypertension : JASH*. 2008;2(2):106-115. doi:10.1016/j.jash.2007.10.005
125. Sanchez-Aguilar M, Ibarra-Lara L, Del Valle-Mondragon L, et al. Rosiglitazone, a Ligand to PPAR gamma, Improves Blood Pressure and Vascular Function through Renin-Angiotensin System Regulation. *PPAR Research*. 2019;2019:1371758. doi:http://dx.doi.org/10.1155/2019/1371758
126. Scroggin M PK. The PPAR-{gamma} agonist Rosiglitazone increases Angiotensin-Converting Enzyme 2 (ACE2) promoter activity in neurons. *FASEB Journal*. 2012;26(Meeting Abstracts).
127. Zhang W, Li C, Liu B, et al. Pioglitazone upregulates hepatic angiotensin converting enzyme 2 expression in rats with steatohepatitis. *Annals of hepatology*. 2013;12(6):892-900.
128. Aguilar MS, Aguilar-Navarro A, Ibarra-Lara L, et al. PPAR Ganna stimulation by rosiglitazone decreases blood pressure and renal apoptosis in a rat hypertension model secondary to aortic coartaction. *Journal of Hypertension*. 2018;36(Supplement 1):e43. doi:10.1097/01.hjh.0000539075.61733.23
129. Chodavarapu H, Grobe N, Somineni HK, et al. Rosiglitazone Treatment of Type 2 Diabetic db/db Mice Attenuates Urinary Albumin and Angiotensin Converting Enzyme 2 Excretion. *PLoS ONE*. 2013;8(4):e62833. doi:10.1371/journal.pone.0062833
130. Ali RM, Al-Shorbagy MY, Helmy MW, et al. Role of Wnt4/beta-catenin, Ang II/TGFbeta, ACE2, NF-kappaB, and IL-18 in attenuating renal ischemia/reperfusion-induced injury in rats treated with Vit D and pioglitazone. *European Journal of Pharmacology*. 2018;831:68-76. doi:http://dx.doi.org/10.1016/j.ejphar.2018.04.032
131. Andersen LB, Przybyl L, Haase N, et al. Vitamin D depletion aggravates hypertension and target-organ damage. *Journal of the American Heart Association*. 2015;4(2):e001417. doi:http://dx.doi.org/10.1161/JAHA.114.001417
132. da Silva Machado C, Alexandre FH C de ML et al. Vitamin D3 regulates genes involved with the renin-angiotensin-aldosterone system in hypertensive and normotensive rats. *Toxicology Letters*. 2014;229(SUPPL. 1):S231. doi:10.1016/j.toxlet.2014.06.773
133. Lin M, Gao P, Zhao T, et al. Calcitriol regulates angiotensin-converting enzyme and angiotensin converting-enzyme 2 in diabetic kidney disease. *Molecular biology reports*. 2016;43(5):397-406. doi:10.1007/s11033-016-3971-5
134. Xu J, Yang J, Chen J, Luo Q, Zhang Q, Zhang H. Vitamin D alleviates lipopolysaccharideinduced acute lung injury via regulation of the reninangiotensin system. *Molecular medicine reports*. 2017;16(5):7432-7438. doi:10.3892/mmr.2017.7546
135. Speth R, Carrera E J-BM et al. Concentration-dependent effects of zinc on angiotensin-converting enzyme-2 activity. *FASEB Journal*. 2014;28(1 SUPPL. 1).

Table 2: Characteristics and key findings of included studies

*Studies reporting on multiple sites or in multiple models have been listed separately and appear more than once in the table

Drug class	Sample size, median (range)	Exposure to treatment, mean (SD)	Effect on ACE2 expression, levels or activity (number of studies) *	Model tested (number of studies)*	Site of ACE2 receptor measurement (number of studies)*	Condition of subjects (number of studies)*
ACE inhibitors [26,27,36–45,28,46,47,29–35]	32 (7 to 375)	4 weeks (3)	Increase (n= 17)<br>Decrease (n= 1)<br>No effect (n= 4)	Rats, in vivo (n= 16)<br>Humans, in vivo (n= 2)<br>Other (n =4)	Cardiac (n= 14)<br>Hepatic (n= 2)<br>Renal (n= 2)<br>Not stated (n= 2)<br>Other (n= 3)	Heart disease/heart failure (n= 7)<br>Hypertension (n= 3)<br>Diabetes (n= 3)<br>Healthy (n=2)<br>Other (n= 1)<br>Not stated (n= 6)
Aldosterone antagonists [48–50]	63 (28 to 75)	4 weeks (4)	Increase (n= 3)<br>Decrease (n= 0)<br>No effect (n= 0)	Rats, in vivo (n = 3)	Renal (n= 3)	Diabetes (n= 1)<br>Renal disease (n= 1)<br>Hepatic dysfunction (n= 1)
ARBs [27,28,53–62,29,63–72,31,73–82,36,83–92,37,93–96,39,42,51,52]	36 (6 to 180)	6 weeks (6)	Increase (n= 43)<br>Decrease (n= 7)<br>No effect (n= 3)<br>Unclear/mixed findings (n =2)	Rats, in vivo (n= 34)<br>Mice, in vivo (n = 11)<br>Humans - in vivo (n= 4)<br>Other (n= 6)	Cardiac (n= 27)<br>Hepatic (n= 2)<br>Renal (n= 12)<br>Lung (n = 2)<br>Not stated (n= 1)<br>Other (n= 7)	Hypertension (n= 13)<br>Heart disease/heart failure (n= 10)<br>Diabetes (n= 5)<br>Healthy (n=6)<br>Not stated (n= 13)<br>Other (n= 5)

Beta Blockers [79,97]	52 (44 to 62)	4 weeks (2)	Increase (n= 0)<br>Decrease (n= 0)<br>No effect (n= 2)	Rats, in vivo (n = 1)<br>Rats, in vitro (n = 1)	Cardiac (n= 2)	Hypertension (n= 2)
Calcium channel blockers [68,98,99]	117 (N/A)	3 weeks (1)	Increase (n= 2)<br>Decrease (n= 1)<br>No effect (n= 0)	Rats, in vivo (n = 2)<br>Human, in vitro (n = 1	Hepatic (n= 1)<br>Cardiac (n= 1)<br>Cerebral (n =1)	Healthy (n= 2)<br>Hypertension (n= 1)
Centrally-acting vasodilators [53]	6 (N/A)	8 weeks (N/A)	Increase (n= 0)<br>Decrease (n= 0)<br>No effect (n= 1)	Mice, in vitro (n= 1)	Cardiac (n= 1)	Other (n= 1)
DPP4 inhibitor [100]	24 (N/A)	4 weeks (N/A)	Increase (n= 1)<br>Decrease (n= 0)<br>No effect (n= 0)	Rats, in vivo (n = 1)	Cardiac (n= 1)	Healthy (n=1)

For peer review only

GABA analogues [101]	8 (N/A)	3 weeks (N/A)	Increase (n= 0)<br>Decrease (n= 1)<br>No effect (n= 0)	Rats, in vivo (n= 1)	Cerebral (n= 1)	Heathy (n= 1)
GLP-1 agonists [100,102]	38 (24 to 54)	3 weeks (1)	Increase (n= 2)<br>Decrease (n= 0)<br>No effect (n= 0)	Rats, in vivo (n = 2)	Cardiac (n= 1)<br>Lung (n= 1)	Diabetic (n=1)<br>Healthy (n=1)
Insulin [103–110]	57 (8 to 84)	6 weeks (6)	Increase (n= 6)<br>Decrease (n= 1)<br>No effect (n= 1)	Mice, in vivo (n = 4)<br>Mice, in vitro (n = 1)<br>Rats, in vivo (n = 2)<br>Rats, in vitro (n = 1)	Renal (n= 5)<br>Cardiac (n= 2)	Diabetes (n= 7)<br>Healthy (n= 1)
Ivabridine [111]	24 (N/A)	12 weeks (N/A)	Increase (n= 1)<br>Decrease (n= 0)<br>No effect (n= 0)	Canine, in vivo (n= 1)	Cardiac (n= 1)	Heart failure (n= 1)

For peer review only

NSAIDs [112,113]	18 (N/A)	8 weeks (0)	Increase (n= 2)<br>Decrease (n= 0)<br>No effect (n= 0)	Rats, in vivo (n = 2)	Cardiac (n= 2)	Diabetic (n=2)
Oestrogens [62,114–118]	27 (17 to 75)	3 weeks (3)	Increase (n= 3)<br>Decrease (n= 1)<br>No effect (n= 1)	Human, in vitro (n = 2)<br>Human, in vivo (n = 1)<br>Rats, in vivo (n = 2)<br>Mice, in vivo (n = 1)	Cardiac (n= 2)<br>Ovarian (n= 1)<br>Cerebral (n= 1)<br>Not stated (n= 2)	Heart disease (n= 1)<br>Hypertension (n= 1)<br>Hypertension + ovariectomy (n= 1)<br>Healthy (n= 1)<br>Not stated (n= 1)<br>Alzheimers (n=1)
PDE-5 inhibitors [119]	32	30 minutes (N/A)	Increase (n= 0)<br>Decrease (n= 0)<br>No effect (n= 1)	Rats, in vitro (n = 1)	Cardiac (n= 1)	Healthy (n=1)
SGLT2 inhibitors [120]	Not stated	15 weeks (N/A)	Increase (n= 0)<br>Decrease (n= 0)<br>No effect (n= 1)	Mice, in vitro (n= 1)	Renal (n= 1)	Diabetic (n= 1)

For peer review only

Statins [99,110,121–123]	62 (36 to 87)	5 weeks (3)	Increase (n= 3)<br>Decrease (n= 1)<br>No effect (n= 1)	Rats, in vivo (n = 4)<br>Rats, in vitro (n = 1)	Cardiac (n= 4)<br>Renal (n= 1)	Diabetes (n= 2)<br>Hypertension (n= 1)<br>Unclear (n= 1)<br>Unclear (n= 1)
Thiazide and thiazide-like diuretics [124]	48 (N/A)	1 week (N/A)	Increase (n= 1)<br>Decrease (n= 1)<br>No effect (n= 0)	Rats, in vivo (n = 1)	Cardiac (n= 1)	Hypertension (n=1)<br>Healthy (n=1)
Thiazolidinedione [112,125–130]	21 (8 to 60)	6 weeks (8)	Increase (n= 5)<br>Decrease (n= 1)<br>No effect (n= 1)	Rats, in vivo (n = 4)<br>Rats, in vitro (n = 1)<br>Human, in vitro (n = 1)<br>Mice, in vivo (n = 1)	Renal (n= 3)<br>Cardiac (n= 2)<br>Hepatic (n= 1)<br>Cerebral (n =1)	Hypertension (n= 1)<br>Heart disease/heart failure (n= 1)<br>Diabetes (n= 1)<br>Healthy (n=1)<br>Renal disease (n= 1)<br>Not stated (n= 2)
Vitamin D [130–132]	47 (33 to 60)	6 weeks (6)	Increase (n= 2)<br>Decrease (n= 0)<br>No effect (n= 1)	Rats, in vivo (n= 3)	Cardiac (n= 1)<br>Renal (n= 1)<br>Not stated (n= 0)	Hypertension (n= 1)<br>Renal disease (n= 1)<br>Not stated (n= 1)
Vitamin D analogues [133,134]	28 (25 to 30)	8 weeks (11)	Increase (n= 1)<br>Decrease (n= 0)<br>No effect (n= 1)	Rats, in vivo (n= 1)<br>Rats, in vitro (n= 1)	Renal (n= 1)<br>Lung (n = 1)	Diabetes (n= 5)<br>Lung injury (n=1)
Zinc [135]	Not staed	Not stated	Increase (n= 0)<br>Decrease (n= 1)<br>No effect (n= 0)	Rats, in vitro (n= 1)	Renal (n= 1)<br>Lung (n= 1)	Not stated (n= 1)

For peer review only

Table 3: Summary of study characteristics with human models

Drug Class	Sample size, median (range)	Exposure to treatment, mean (SD)	Effect on ACE2 expression, levels or activity (number of studies)	Model tested	Site of ACE2 receptors	Condition of subject
Angiotensin-Receptor Blockers	46.5 (8 to 80)	15 weeks (6)	Increase (n= 2)<br>Decrease (n= 1)<br>No effect (n= 1)	In vivo (n= 3)<br>In vitro (n= 1)	Urinary (n= 1)<br>Serum (n= 1)<br>Renal (n= 1)<br>Not stated (n= 1)	Diabetes (n= 1)<br>Hypertension (n= 1)<br>Hypertension + Diabetes (n= 1)<br>Diabetic + Chronic Kidney Disease (n= 1)
Angiotensin Converting Enzyme- Inhibitors	228 (80 to 375)	12 weeks (N/A)	Increase (n= 1)<br>Decrease (n= 1)	In vitro (n= 2)	Renal (n= 1)<br>Unclear (n= 1)	Diabetic + Chronic Kidney Disease (n= 1)<br>Unclear (n= 1)
Calcium Channel Blockers	N/A	Unclear	Increase ACE2 in the membrane surface (decreased in the cytosol) (n= 1)	In vitro (n= 1)	Cardiac	Healthy cells
Oestrogen	36 (N/A)	1 day (N/A)	Increase (n= 2)<br>No effect (n= 1)	In vitro (n= 3)	Cardiac (n= 1)<br>Umbilical (n= 1)<br>Not stated (n= 1)	Heart problems (n= 1)<br>Healthy (n= 1)<br>Not stated (n= 1)
Thiazolidinedione	Not stated	1 day	Increase (n= 1)	In vitro (n= 1)	Cerebral (n= 1)	Not stated

For peer review only

Figure 1: PRISMA flow chart explaining the study inclusion process

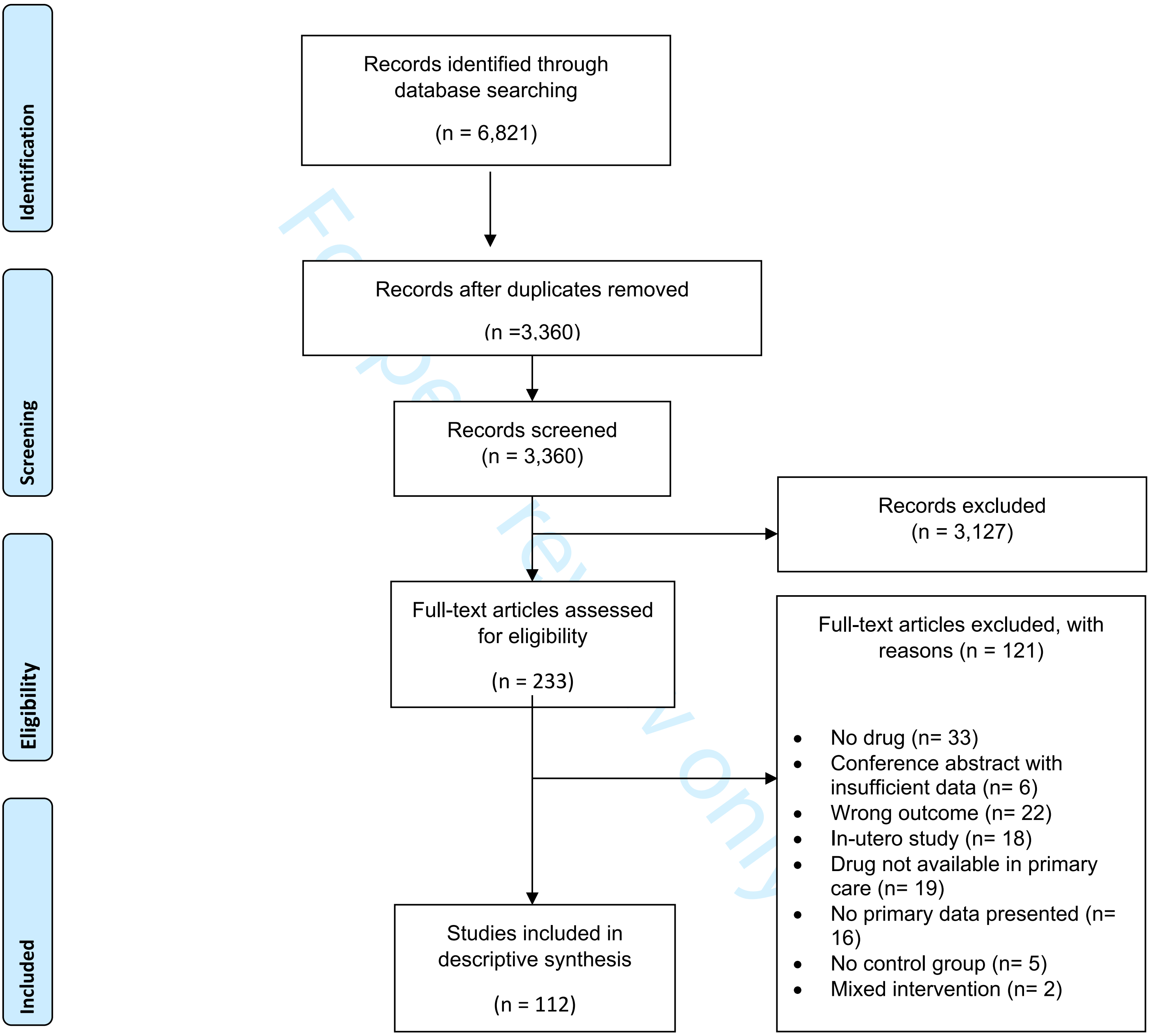

Figure 2: Risk of bias assessment

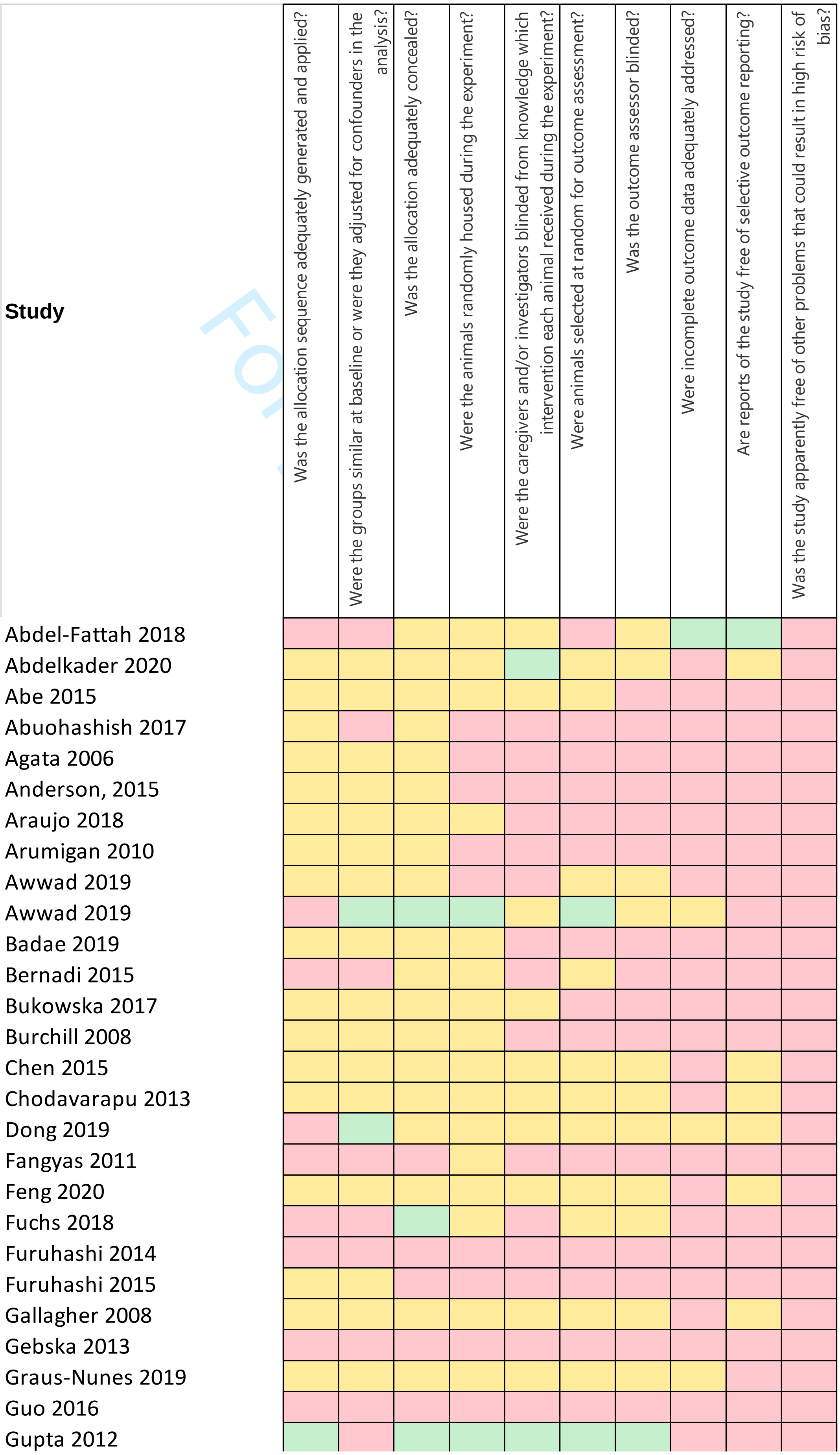

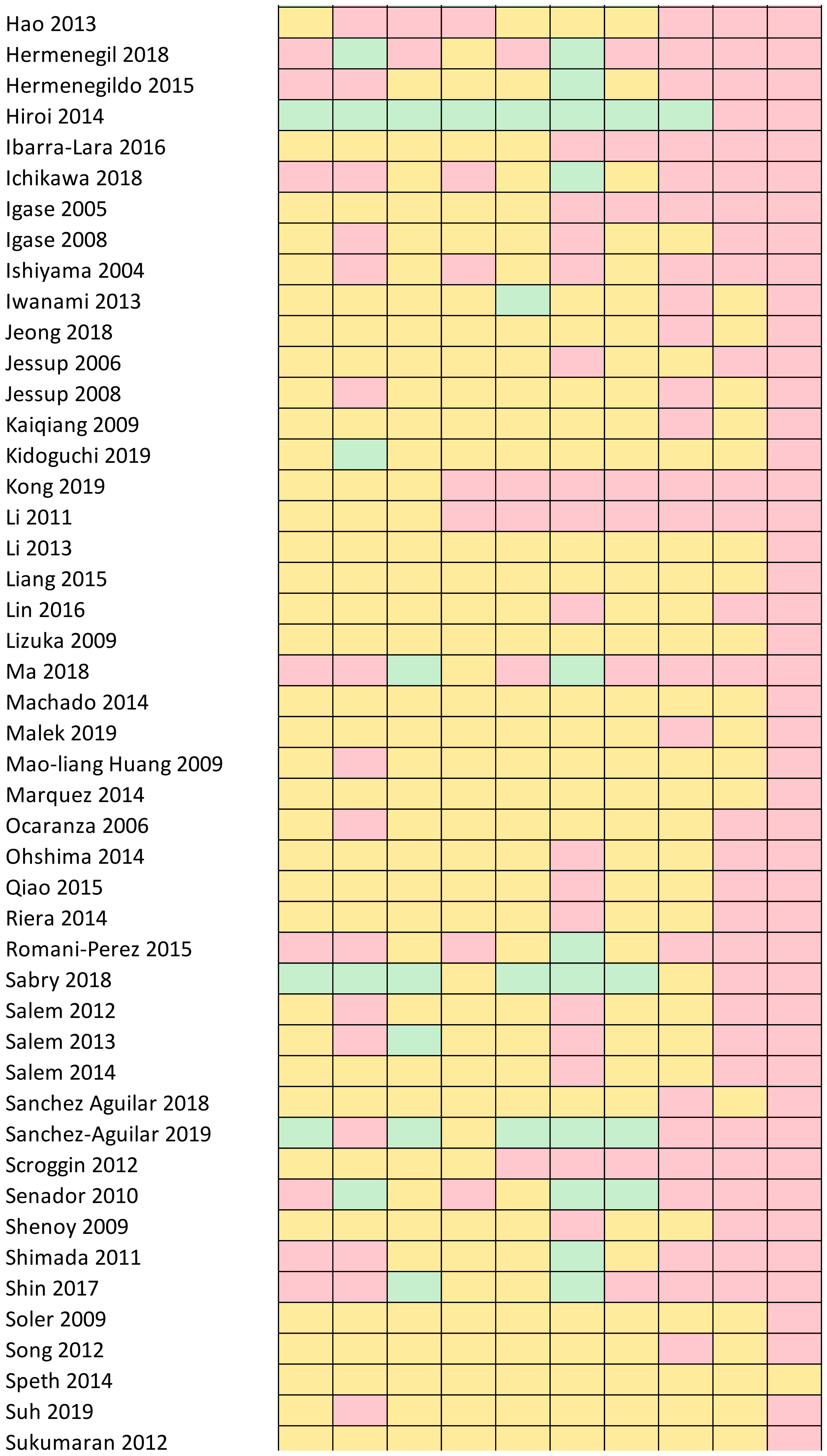
Hao 2013
Hermenegil 2018
Hermenegildo 2015
Hiroi 2014
Ibarra-Lara 2016
Ichikawa 2018
Igase 2005
Igase 2008
Ishiyama 2004
Iwanami 2013
Jeong 2018
Jessup 2006
Jessup 2008
Kaiqiang 2009
Kidoguchi 2019
Kong 2019
Li 2011
Li 2013
Liang 2015
Lin 2016
Lizuka 2009
Ma 2018
Machado 2014
Malek 2019
Mao-liang Huang 2009
Marquez 2014
Ocaranza 2006
Ohshima 2014
Qiao 2015
Riera 2014
Romani-Perez 2015
Sabry 2018
Salem 2012
Salem 2013
Salem 2014
Sanchez Aguilar 2018
Sanchez-Aguilar 2019
Scroggin 2012
Senador 2010
Shenoy 2009
Shimada 2011
Shin 2017
Soler 2009
Song 2012
Speth 2014
Suh 2019
Sukumaran 2012

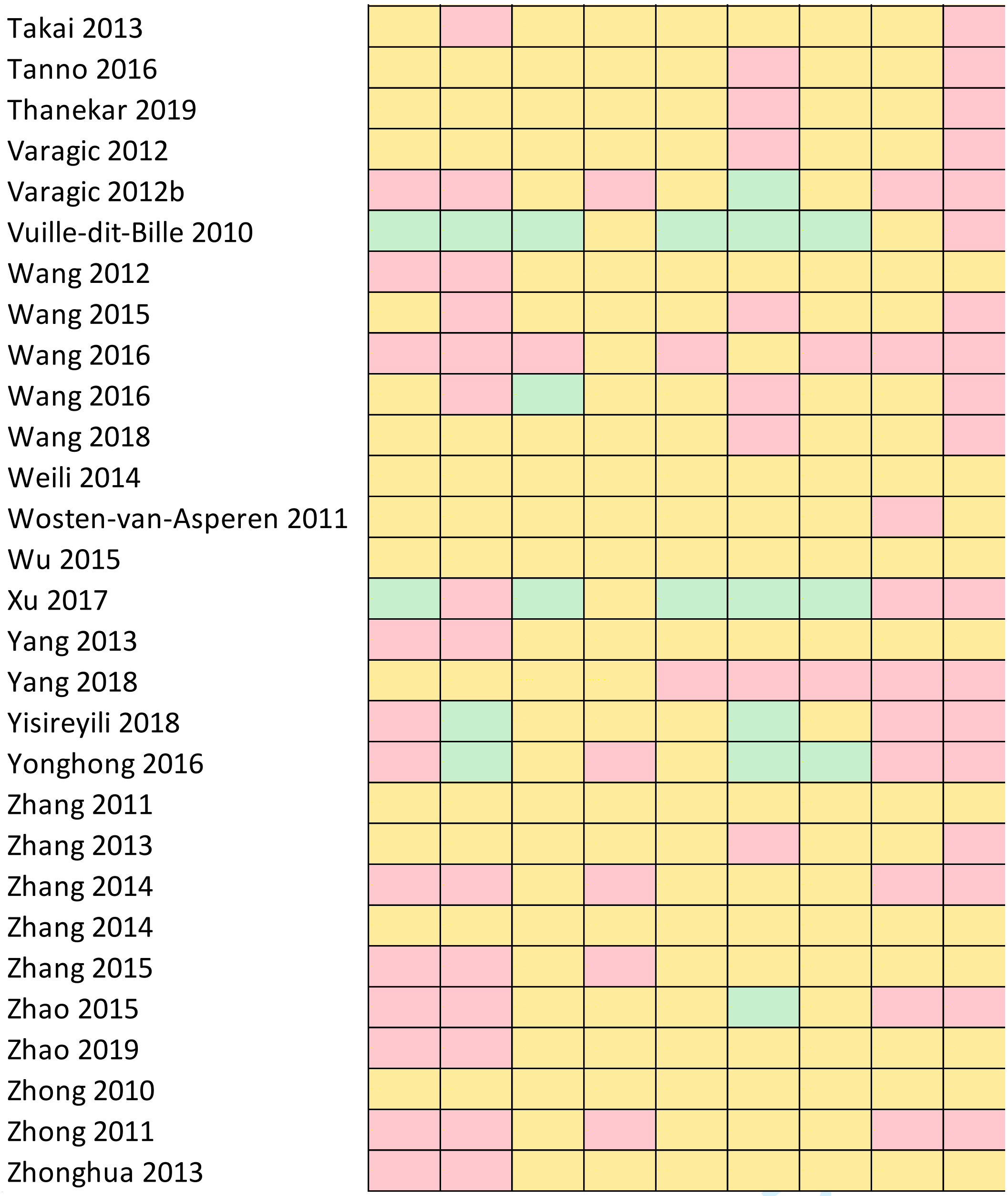
Takai 2013
Tanno 2016
Thanekar 2019
Varagic 2012
Varagic 2012b
Vuille-dit-Bille 2010
Wang 2012
Wang 2015
Wang 2016
Wang 2016
Wang 2018
Weili 2014
Wosten-van-Asperen 2011
Wu 2015
Xu 2017
Yang 2013
Yang 2018
Yisireyili 2018
Yonghong 2016
Zhang 2011
Zhang 2013
Zhang 2014
Zhang 2014
Zhang 2015
Zhao 2015
Zhao 2019
Zhong 2010
Zhong 2011
Zhonghua 2013

PRISMA 2009 Checklist

Section/topic	#	Checklist item	Reported on page #
**TITLE**			
Title	1	Identify the report as a systematic review, meta-analysis, or both.	1
**ABSTRACT**			
Structured summary	2	Provide a structured summary including, as applicable: background; objectives; data sources; study eligibility criteria, participants, and interventions; study appraisal and synthesis methods; results; limitations; conclusions and implications of key findings; systematic review registration number.	2
**INTRODUCTION**			
Rationale	3	Describe the rationale for the review in the context of what is already known.	4
Objectives	4	Provide an explicit statement of questions being addressed with reference to participants, interventions, comparisons, outcomes, and study design (PICOS).	4
**METHODS**			
Protocol and registration	5	Indicate if a review protocol exists, if and where it can be accessed (e.g., Web address), and, if available, provide registration information including registration number.	5
Eligibility criteria	6	Specify study characteristics (e.g., PICOS, length of follow-up) and report characteristics (e.g., years considered, language, publication status) used as criteria for eligibility, giving rationale.	5
Information sources	7	Describe all information sources (e.g., databases with dates of coverage, contact with study authors to identify additional studies) in the search and date last searched.	5
Search	8	Present full electronic search strategy for at least one database, including any limits used, such that it could be repeated.	5 + table 1
Study selection	9	State the process for selecting studies (i.e., screening, eligibility, included in systematic review, and, if applicable, included in the meta-analysis).	5
Data collection process	10	Describe method of data extraction from reports (e.g., piloted forms, independently, in duplicate) and any processes for obtaining and confirming data from investigators.	5
Data items	11	List and define all variables for which data were sought (e.g., PICOS, funding sources) and any assumptions and simplifications made.	5
Risk of bias in individual studies	12	Describe methods used for assessing risk of bias of individual studies (including specification of whether this was done at the study or outcome level), and how this information is to be used in any data synthesis.	5
Summary measures	13	State the principal summary measures (e.g., risk ratio, difference in means).	5
Synthesis of results	14	Describe the methods of handling data and combining results of studies, if done, including measures of consistency (e.g., I^2) for each meta-analysis.	5

For peer review only

For peer review only - http://bmjopen.bmj.com/site/about/guidelines.xhtml

PRISMA 2009 Checklist

Section/topic	#	Checklist item	Reported on page #
Risk of bias across studies	15	Specify any assessment of risk of bias that may affect the cumulative evidence (e.g., publication bias, selective reporting within studies).	5
Additional analyses	16	Describe methods of additional analyses (e.g., sensitivity or subgroup analyses, meta-regression), if done, indicating which were pre-specified.	none
**RESULTS**			
Study selection	17	Give numbers of studies screened, assessed for eligibility, and included in the review, with reasons for exclusions at each stage, ideally with a flow diagram.	6
Study characteristics	18	For each study, present characteristics for which data were extracted (e.g., study size, PICOS, follow-up period) and provide the citations.	6 +table 2
Risk of bias within studies	19	Present data on risk of bias of each study and, if available, any outcome level assessment (see item 12).	Table 4
Results of individual studies	20	For all outcomes considered (benefits or harms), present, for each study: (a) simple summary data for each intervention group (b) effect estimates and confidence intervals, ideally with a forest plot.	Table 2 and 3 page 6
Synthesis of results	21	Present results of each meta-analysis done, including confidence intervals and measures of consistency.	No meta-analysis
Risk of bias across studies	22	Present results of any assessment of risk of bias across studies (see Item 15).	Table4
Additional analysis	23	Give results of additional analyses, if done (e.g., sensitivity or subgroup analyses, meta-regression [see Item 16]).	None
**DISCUSSION**			
Summary of evidence	24	Summarize the main findings including the strength of evidence for each main outcome; consider their relevance to key groups (e.g., healthcare providers, users, and policy makers).	7
Limitations	25	Discuss limitations at study and outcome level (e.g., risk of bias), and at review-level (e.g., incomplete retrieval of identified research, reporting bias).	8
Conclusions	26	Provide a general interpretation of the results in the context of other evidence, and implications for future research.	8
**FUNDING**			
Funding	27	Describe sources of funding for the systematic review and other support (e.g., supply of data); role of funders for the systematic review.	9

From: Moher D, Liberati A, Tetzlaff J, Altman DG, The PRISMA Group (2009). Preferred Reporting Items for Systematic Reviews and Meta-Analyses: The PRISMA Statement. PLoS Med 6(7): e1000097. doi:10.1371/journal.pmed1000097

For peer review only - http://bmjopen.bmj.com/site/about/guidelines.xhtml

PRISMA 2009 Checklist

For more information, visit: **www.prisma-statement.org**.

Page 2 of 2

For peer review only

Currently prescribed drugs in the UK that could up or downregulate ACE2 in COVID-19 disease: A systematic review

Journal:	*BMJ Open*
Manuscript ID	bmjopen-2020-040644.R1
Article Type:	Original research
Date Submitted by the Author:	02-Jul-2020
Complete List of Authors:	Dambha-Miller, Hajira; University of Southampton Faculty of Medicine, Department of Primary Care; University of Cambridge, MRC Epidemiology Unit<br>Albasri, Ali; Department of Primary Care Health Sciences, University of Oxford, ; University of Oxford<br>Hodgson, Sam; University of Southampton Faculty of Medicine, Department of Primary Care<br>Wilcox, Christopher; University of Southampton Faculty of Medicine, Department of Primary Care<br>Khan, Shareen; Oxford University Hospitals NHS Trust<br>Islam, Nazrul ; University of Oxford Nuffield Department of Population Health; University of Cambridge, MRC Epidemiology Unit<br>Little, Paul; University of Southampton, Medical School,<br>Griffin, Simon; University of Cambridge School of Clinical Medicine
**Primary Subject Heading**:	Infectious diseases
Secondary Subject Heading:	Pharmacology and therapeutics
Keywords:	Public health < INFECTIOUS DISEASES, Respiratory infections < THORACIC MEDICINE, Adverse events < THERAPEUTICS

I, the Submitting Author has the right to grant and does grant on behalf of all authors of the Work (as defined in the below author licence), an exclusive licence and/or a non-exclusive licence for contributions from authors who are: i) UK Crown employees; ii) where BMJ has agreed a CC-BY licence shall apply, and/or iii) in accordance with the terms applicable for US Federal Government officers or employees acting as part of their official duties; on a worldwide, perpetual, irrevocable, royalty-free basis to BMJ Publishing Group Ltd ("BMJ") its licensees and where the relevant Journal is co-owned by BMJ to the co-owners of the Journal, to publish the Work in this journal and any other BMJ products and to exploit all rights, as set out in our licence.

The Submitting Author accepts and understands that any supply made under these terms is made by BMJ to the Submitting Author unless you are acting as an employee on behalf of your employer or a postgraduate student of an affiliated institution which is paying any applicable article publishing charge ("APC") for Open Access articles. Where the Submitting Author wishes to make the Work available on an Open Access basis (and intends to pay the relevant APC), the terms of reuse of such Open Access shall be governed by a Creative Commons licence – details of these licences and which Creative Commons licence will apply to this Work are set out in our licence referred to above.

Other than as permitted in any relevant BMJ Author's Self Archiving Policies, I confirm this Work has not been accepted for publication elsewhere, is not being considered for publication elsewhere and does not duplicate material already published. I confirm all authors consent to publication of this Work and authorise the granting of this licence.

For peer review only

Currently prescribed drugs in the UK that could up or downregulate ACE2 in COVID-19 disease: A systematic review

Short title: Drugs using ACE2 in COVID-19

Hajira Dambha-Miller[1,2] MRCGP, PhD, Ali Albasri[3] DPhil, Sam Hodgson BM BCh [1], Christopher R Wilcox MBBCh[1], Shareen Khan MPharm [4], Nazrul Islam MBBS, PhD [2,5], Paul Little FMedSci [1] and Simon J Griffin FMedSci [2,6].

1. Division of Primary Care and Population Health, University of Southampton
2. Medical Research Council Epidemiology Unit, University of Cambridge, Cambridge, United Kingdom
3. Nuffield Department of Primary Care Health Sciences, University of Oxford
4. Oxford University Hospitals NHS Foundation Trust
5. Clinical Trial Service Unit and Epidemiological Studies Unit, Nuffield Department of Population Health, University of Oxford, Oxford, United Kingdom
6. Department of Public Health and Primary Care, School of Clinical Medicine, University of Cambridge

Correspondence: Dr Hajira Dambha-Miller, Primary Care and Population Health, University of Southampton, Southampton, SO16 5ST, Email: hajiradambha@doctors.org.uk

Word count: 2846

Abstract

Objective: To review evidence on routinely prescribed drugs in the UK that could up or downregulate Angiotensin Converting Enzyme 2 (ACE2) and potentially affect COVID-19 disease

Design: Systematic review

Data source: MEDLINE, EMBASE, CINAHL, the Cochrane Library and Web of Science

Study selection: Any design with animal or human models examining a currently prescribed UK drug compared to a control, placebo or sham group, and reporting an effect on ACE2 level, activity or gene expression.

Data extraction and synthesis: MEDLINE, EMBASE, CINAHL, the Cochrane Library, Web of Science and OpenGrey from inception to 1st April 2020. Methodological quality was assessed using the SYRCLE's risk of bias tool for animal studies and Cochrane risk of bias tool for human studies.

Results: We screened 3,360 titles and included 112 studies with 21 different drug classes identified as influencing ACE2 activity. Ten studies were in humans and 102 were in animal models None examined ACE2 in human lungs. The most frequently examined drugs were Angiotensin Receptor Blockers (ARBs) (n= 55) and Angiotensin-Converting Enzyme- Inhibitors (ACE-I) (n= 22). More studies reported upregulation than downregulation with ACE-I (n=22), ARBs (n=55), insulin (n=8), thiazolidinedione (n=7) aldosterone agonists (n=3), statins (n=5), oestrogens (n=5) calcium channel-blockers (n=3) GLP-1 agonists (n=2) and NSAIDs (n=2).

For peer review only

Conclusions: There is an abundance of academic literature and media reports on the potential of drugs that could attenuate or exacerbate COVID-19 disease. This is leading to trials of repurposed drugs and uncertainty amongst patients and clinicians concerning continuation or cessation of prescribed medications. Our review indicates that the impact of currently prescribed drugs on ACE2 has been poorly studied in-vivo, particularly in human lungs where the SARS-CoV-2 virus appears to enact its pathogenic effects. We found no convincing evidence to justify starting or stopping currently prescribed drugs to influence outcomes of COVID-19 disease.

Strengths and limitations

- Human and animal models both in vivo and in vitro were included for a comprehensive review
- This is the first systematic review on UK prescribed drugs that could alter ACE2 in COVID-19 disease
- Meta-analysis was not possible due to heterogeneity
- Methodological quality of the studies was low overall

For peer review only

INTRODUCTION

The coronavirus SARS-CoV-2 which causes the COVID-19 disease is a global public health emergency. It has been reported in 190 countries with 4,310,786 confirmed cases and 290,455 deaths as of 12th May 2020. Walker and colleagues from the World Health Organization Collaborating Centre for Infectious Disease Modelling predicted that in the absence of mitigation strategies, the virus would infect seven billion people and account for 40 million deaths this year alone.[1] Efforts to shield the elderly (60% reduction in social contacts) and interrupt transmission (40% reduction in social contacts for the wider population) have reduced this number but further deaths are still expected.[1] There is an urgent need for solutions. In the absence of a vaccination or effective treatment, there is growing interest in repurposing existing drugs for mitigation.

In particular, drugs affecting the Renin-Angiotensin System (RAS) have been highlighted as potential candidates for further investigation.[2,3] This is because the SARS-CoV-2 virus utilises Angiotensin-Converting Enzyme 2 (ACE2) receptors within the RAS for entry into lung alveolar epithelial cells.[4] ACE2 has previously been shown to correlate with susceptibility to the SARS-CoV-1 virus, and the spike (S) glycoprotein of this new virus binds to ACE2 with even higher affinity.[5,6] Theoretically, altered ACE2 activity could, therefore, lead to a greater susceptibility to SARS-CoV-2. It could also cause greater severity of the infection.[7] Previous studies suggest that dysregulation of ACE2 activity in the lungs could promote early neutrophil infiltration and subsequent uncontrolled activation of the RAS system.[8] In mice models, acute lung injury was observed in response to SARS-CoV-1 spike protein, so it is plausible that similar responses will be observed with SARS-CoV-2.[9]This is particularly problematic in organs containing high ACE2 such as the lungs as it may contribute to cytokine release syndrome (cytokine storm) and the subsequent respiratory failure that has been observed in those who have died from the disease.[7] Many prescribed drugs in common use are known to mediate effects through the RAS pathway. Over 45 million of these prescriptions were issued in the UK last year alone, and of these, 15 million were for Angiotensin Converting Enzyme Inhibitors (ACE-I) and Angiotensin Receptor Blockers (ARBs). Acting through the RAS pathway, these drugs may impact ACE2 regulation but their role in the COVID-19 pandemic is not clear. Given the number of people that are potentially on these drugs, it has caused substantial public concern and clinical uncertainty about continuation or cessation of prescribed medications during the pandemic. Accordingly, we reviewed all existing evidence on routinely prescribed UK drugs that might alter ACE2 regulation. Understanding the drug effects on ACE2 given its role in COVID-19 disease could help reassure clinicians and the public in these uncertain times, or direct research on drugs that might attenuate or exacerbate transmission.

METHODS

Our review was conducted in accordance with preferred reporting for systematic reviews and meta-analyses (PRISMA) guidelines and our protocol was submitted for open-access publication before commencing our study (in press).

Search Strategy

A systematic search in MEDLINE, EMBASE, CINAHL, the Cochrane Library, Web of Science was conducted from inception to the 1st April 2020. The full search strategy for all databases is shown in the supplementary material 1. The reference lists of recent reviews and included studies were screened. We also spoke to topic experts and screened OpenGrey for additional texts. No language limits or study design filters were applied.

For peer review only - http://bmjopen.bmj.com/site/about/guidelines.xhtml

Study selection, inclusion and exclusion criteria

The COVID-19 disease is still relatively new and there is limited research on drug therapies specific to the virus. In the interest of being comprehensive about potential drugs acting through ACE2, we were as inclusive as possible within our study selection. We included both animal and human models (in vivo and in-vitro). Studies had to meet the following eligibility criteria: i) measures ACE2 levels, activity or gene expression, ii) includes a drug that is currently available on a UK prescription according to the British National Formulary, and iii) measures the effect of that drug against a placebo, control or sham group in an experimental design. Review articles were excluded but their reference lists were screened. Conference abstracts were included if sufficient detail could be elicited. We did not include studies in children under 18 years, or those examining drug effects in utero.

Data extraction

Four members of the team reviewed titles and abstracts for eligibility (AA, HDM, CW, SH). Full-text review, data extraction and quality assessment were carried out in duplicate using a piloted sheet. Any disagreement between authors was resolved by discussion. Data on the following study characteristics were extracted: i) drug class, ii) drug name, iii) duration of treatment, iii) effect on ACE2 level (upregulation, downregulation, no effect), iv) model (human/rat etc), v) site of ACE2 reception (lung, renal, cardiac etc;), vi) study design, vii) study population, viii) sample size and ix) country. Given the urgency of our research question during the current pandemic, we extracted information from only what was available to us in the published text.

Quality assessment

Our review includes both animal and human models, therefore, quality assessment was carried out separately for these studies. Human studies were evaluated using the Cochrane risk of bias tool which includes the following domains: random sequence generation, allocation concealment, blinding of participants and personnel, blinding of outcome assessment, incomplete outcome data, selective reporting, and other sources of bias.[10] Each domain was scored as low risk, unclear risk, or high risk of bias. We classified the overall risk of bias as low if all domains were at low risk of bias, as high if at least one domain was at high risk of bias, or as unclear if at least one domain was at unclear and no domain was at high risk of bias. Although this tool is specific to trials, all included studies were experimental designs and we therefore felt it appropriate to use. The methodological quality of animal studies was assessed using the SYRCLE's risk of bias tool which is based on the Cochrane risk of bias tool.[11] SYRCLE's tool includes selection bias, performance bias, detection bias, attrition bias, reporting bias and other biases.

Data analysis

Owing to the mix of study designs and models, a meta-analysis was not appropriate. Narrative synthesis methods were used. We reviewed the meta-data by tabulating the studies according to our inclusion/exclusion criteria, human/animal model, drug classes and effects on ACE2. The consistency in the number of studies and direction of any effects were considered. Where inconsistencies were identified in the effect of a drug between studies, we looked at additional data such as methods, quality and outcome measurement for potential explanatory factors.

Patient and public involvement

It was not possible to involve patients or the public in the design or conduct of our work due to the rapid timelines, but we have invited PPI representatives to help us with drafting a lay summary and in the dissemination of our findings.

Results

We retrieved 6827 studies and screened 3,360 after removing duplicates. Following title and abstract screening, 233 studies were screened by full text. We included 112 studies in the final review. The flow of studies is shown in a PRISMA diagram in figure 1 including the reasons for study exclusion at each stage. A baseline table of included studies is included in the supplementary material 2

Study characteristics

Table 1 shows the characteristics of the included studies. These originated from 17 different countries with the most common being China (n= 36), the USA (n= 22) and Japan (n=18). There were 10 studies in humans (7 in-vitro and 3 in-vivo) and 102 in animal models (13 in-vitro and 89 in-vivo). Animal models included rats (n=94), mice (n=7) and canines (n=1). The sample sizes for in-vivo animal models ranged from 6 to 117. For in-vivo human studies (Table 2), sample sizes ranged from 8 to 375 but were not always reported. Participants were predominantly male and white with hypertension or diabetes, although the condition was not always stated. Most models examined ACE2 receptors in the heart or kidneys, only 5 of the 112 included studies reporting ACE2 levels in the lungs; these were all in animal models.

Effects of drugs

There were 21 different drug classes examined in the included studies. Table 2 tabulates only those that have been examined in human models. The mean drug exposure period ranged from 30 mins for in-vitro studies, to 15 weeks for in-vivo studies. The most common drug classes were ARBs (n= 55) and ACE-I (n= 22). Of the 55 studies that examined ARBs, 43 reported upregulation of ACE2 levels. Most of these studies were in rat models (n= 34) and examined cardiac ACE2 levels (n= 27). For ACE-I, 17 out of 22 studies reported upregulation of ACE2. These were also mainly in rat models (n= 16) and measured cardiac ACE2 levels (n= 14). Of the five studies that assessed statins, these were all within rat models; 3 reported upregulation of ACE2, 1 reported downregulation and 1 reported no effect. Similarly, oestrogens were examined in 5 studies; 3 reported upregulation, 1 reported downregulation and 1 reported no effects. For calcium channel blockers; 2 out of the 3 studies reported upregulation of ACE2 levels and these were both in-vivo rodent models. The third study was an in-vitro human model that showed downregulation of ACE2 with a calcium channel blocker. There were 3 studies on aldosterone antagonists; all reporting increases in renal ACE2 levels within rat models.

Several diabetes drugs were evaluated and found to increase ACE2. For insulin, 6 out of 8 studies reported upregulation of ACE2 (in mice and rat models). For thiazolidinediones, 5 out of 7 studies reported upregulation (6 mice/rate models and 1 of cerebral human cells in-vitro). For GLP-1 agonists both included studies reported increases in ACE2. Similarly, for the one study examining DPP4 inhibitors, it also reported an increase in ACE2. The only study measuring the effect of SGLT2 inhibitors reported a decrease in ACE2.

For peer review only

Quality assessment

The risk of bias across the studies has been shown in the supplementary material 3 using the SYRCLE's risk of bias tool. In general, studies lacked blind allocation and outcome assessor blinding. They also frequently omitted information needed to make a thorough judgement on the risk of bias.

Discussion

To our knowledge, this is the most comprehensive review of drugs prescribed in the UK that could act on ACE2 receptors and thus potentially affect COVID-19 disease. The ACE2 receptor is reported to be an essential contributor to SARS-CoV-2 entry into the nasopharynx and lungs and the subsequent inflammation that leads to severe acute respiratory distress syndrome.[12,13] Our review examined drugs across human and animal models, and we found a number of studies reporting upregulation of ACE2 levels in response to ACE-I (n=22), ARBs (n=55), insulin (n=8), thiazolidinedione (n=7) aldosterone agonists (n=3), statins (n=5), oestrogens (n=5) calcium channel-blockers (n=3) GLP-1 agonists (n=2) and NSAIDs (n=2). However, these drugs were poorly studied in vivo within the lungs or nasopharynx of humans, where they are likely to matter most in influencing the severity of outcomes of COVID-19 disease.

We observed that the most frequent drugs to upregulate ACE2 are also those prescribed in people with diabetes or cardiovascular disease. Mortality rates from COVID-19 have been high in this group.[12,14–16] Notably, these are also conditions with a high prevalence amongst Black, Asian and Minority Ethnic (BAME) groups who have had disproportionally high mortality rates from COVID-19 disease.[17] To date, much of this evidence has been limited to clinical commentaries or case reports. Larger cohorts are emerging but have not yet adequately considered a range of potential confounders including co-morbidities, age, sex, deprivation or household numbers which might be more important than prescribed medication in the spread, susceptibility and severity of the disease. For example, in a cohort of 191 people who were infected with the virus in Wuhan, 87% of those who died had coronary heart disease and 47% had diabetes.[18] These conditions are associated with an increased risk of death but were not considered as covariates in the analysis. Irrespective of ACE2, people with diabetes are more susceptible to worse infection as the low-grade chronic inflammation and hyperglycaemia associated with the condition results in impaired immune responses with lower IL-1, IL-6, TNF-a, and delayed mobilisation of immune cells in response to pathogens.[19] This comorbidity like many other confounders, are highly relevant when examining the risk of death with COVID-19 disease.

This lack of adequate adjustment for existing conditions is highlighted by Sommerstein and colleagues in their editorial on ACE-I and ARBs in COVID-19.[15] They also propose that existing co-morbidities such as heart failure may be independently linked to SARS-CoV-2 transmission and severity, and the subsequent poor pulmonary outcomes that are observed in these patients. Indeed, in mice models, arterial hypertension, atrial fibrillation and type 2 diabetes have been shown to upregulate ACE2 levels irrespective of medications.[20,21] Moreover, ACE2 levels are higher in men and with increasing age.[22] Most of the published data on deaths in COVID-19 disease report that males of increasing age are particularly susceptible to poor outcomes.[12,23]

Our review has also highlighted the variable ACE2 levels in different parts of the body with most of the existing literature focussing on renal and cardiac levels. Responses to drugs may vary depending on cell type and location. Although the lung ACE2 is important to COVID-19, it is unclear if overall COVID-19 mortality might be attenuated by cardiovascular ACE-2 activity levels. We also

For peer review only

observed variations in ACE2 levels with drug exposure duration which was relatively short amongst included studies in our review. It is uncertain how dysregulation might continue after starting or stopping these medications. It is also unclear how the observed effects amongst included studies would translate in-vivo in humans and what the net effect on receptor access to the COVID-19 virus is; access to the receptor by the virus may be competitively inhibited by the presence of drugs which also attach to the receptor, so whether upregulation is the key factor in practice is unclear. This is particularly challenging to understand as we found a paucity of data demonstrating the effect of prescribed drugs on ACE2 in the lungs or nasopharynx, where the SARS-CoV-2 virus appears to enact its pathogenic effects. Our results therefore do not provide convincing evidence on the role of any currently prescribed UK drugs acting through ACE2 regulation that could affect COVID-19 disease. Finally, we found a disproportionate number of studies reporting upregulation or 'positive effects' of drugs on ACE2, compared to studies reporting no effect or downregulation. This may reflect a publication bias that is well-established in the literature, especially amongst animal models. [24,25]

Strengths and limitations

We carried out a comprehensive and systematic search of the literature. To our knowledge, this is the first review on the subject. We did not include language restrictions but non-English language studies in the international literature might not have been indexed in the databases we searched. Given the rate of new publications on COVID-19, it is also possible that our search and results may not be up to date. Owing to the limited research on this novel virus, it was necessary to be as inclusive as possible and we therefore considered both animal and human models to look for any drugs acting through ACE2 with potential to affect COVID-19 outcomes. While this inclusive approach may offer insights, the heterogeneity across models makes it hard to interpret findings or translate them directly to patients. We did not formally assess heterogeneity but this is likely as we had multiple different models including animal, human, in-vitro, in-vivo as well as different body sites (heart, lung, kidney etc;) Future studies are needed that can quantify effects through meta-analysis, and examine dose responses. Although we were robust in our methodological approach to this review, we were also aware of the urgency to report our findings in the current pandemic. We therefore did not contact authors for more information about their studies beyond what was published. We observed frequent omission of information that would have allowed us to carry out a more detailed quality assessment. Had we pursued this information; the quality assessment of included papers may well have been higher. At present, all studies were high risk of bias which is a limitation of this review.

Conclusion:

We reviewed the evidence on routinely prescribed drugs in the UK that could up or down-regulate ACE2 and thus potentially affect COVID-19 disease. Our review indicates that currently prescribed drugs have been poorly studied in-vivo within the lungs of humans. Until there is better evidence, we cannot recommend starting or stopping prescribed medications during the COVID-19 pandemic.

Acknowledgements: We would like to thank Prof Julia Hippisley-Cox for her early contributions to this project.

Competing Interests: All authors have completed the ICMJE uniform disclosure form and confirm no financial relationships with any organisations that might have an interest in the submitted work in the previous three years, no other relationships or activities that could appear to have influenced the submitted work.

For peer review only

Funding: The Southampton, Cambridge and Oxford Primary Care Departments are members of the NIHR School for Primary Care Research and supported by NIHR Research funds. The University of Cambridge has received salary support in respect of SJG from the NHS in the East of England through the Clinical Academic Reserve. SJG is supported by an MRC Epidemiology Unit programme: MC_UU_12015/4. HDM is an NIHR Clinical Lecturer. The views expressed are those of the author(s) and not necessarily those of the NHS, the NIHR or the Department of Health and Social Care

Ethical Approval: Ethical approval is not required for a systematic review

Data sharing: Data are available on reasonable request

Author Contribution: HDM contributed to the design of the study, wrote the analysis plan, conducted the analysis, drafted and revised the paper. AA contributed to the design of the study, led the analysis, drafted and revised the paper. CW and SH contributed to the screening of studies, data extraction and revised the paper. SK contributed to screening of studies. NI revised the paper. SG and PL contributed to the design of the study and revised the paper. HDM is guarantor.

Declaration: The lead author affirms that the manuscript is an honest, accurate, and transparent account of the study being reported; that no important aspects of the study have been omitted. The opinions, results, and conclusions reported in this article are those of the authors and are independent from the funding sources.

References

1 Gt Walker P, Whittaker C, Watson O, *et al.* The Global Impact of COVID-19 and Strategies for Mitigation and Suppression.

2 Patel AB, Verma A. COVID-19 and Angiotensin-Converting Enzyme Inhibitors and Angiotensin Receptor Blockers. *JAMA* Published Online First: 24 March 2020. doi:10.1001/jama.2020.4812

3 Fang L, Karakiulakis G, Roth M. Are patients with hypertension and diabetes mellitus at increased risk for COVID-19 infection? Published Online First: 2020. doi:10.1016/S2213-2600(20)30116-8

4 Jia H. Pulmonary Angiotensin-Converting Enzyme 2 (ACE2) and Inflammatory Lung Disease. *SHOCK* 2016;**46**:239–48. doi:10.1097/SHK.0000000000000633

5 Hofmann H, Geier M, Marzi A, *et al.* Susceptibility to SARS coronavirus S protein-driven infection correlates with expression of angiotensin converting enzyme 2 and infection can be blocked by soluble receptor. *Biochemical and Biophysical Research Communications* 2004;**319**:1216–21. doi:10.1016/j.bbrc.2004.05.114

6 Wrapp D, Wang N, Corbett KS, *et al.* Cryo-EM structure of the 2019-nCoV spike in the prefusion conformation. *Science* 2020;**367**:1260–3. doi:10.1126/science.aax0902

7 Shi Y, Wang Y, Shao C, *et al.* COVID-19 infection: the perspectives on immune responses. *Cell death and differentiation* 2020;:1–4. doi:10.1038/s41418-020-0530-3

8 Sodhi CP, Wohlford-Lenane C, Yamaguchi Y, *et al.* Attenuation of pulmonary ACE2 activity impairs inactivation of des-arg9 bradykinin/BKB1R axis and facilitates LPS-induced neutrophil infiltration. *American Journal of Physiology - Lung Cellular and Molecular Physiology* 2018;**314**:L17–31. doi:10.1152/ajplung.00498.2016

9 Kuba K, Imai Y, Rao S, *et al.* A crucial role of angiotensin converting enzyme 2 (ACE2) in SARS coronavirus-induced lung injury. *Nature Medicine* 2005;**11**:875–9. doi:10.1038/nm1267

10 Higgins JPT, Altman DG, Gøtzsche PC, *et al.* The Cochrane Collaboration's tool for assessing risk of bias in randomised trials. *BMJ (Online)* 2011;**343**. doi:10.1136/bmj.d5928

11 Hooijmans CR, Rovers MM, De Vries RBM, *et al.* SYRCLE's risk of bias tool for animal studies. *BMC Medical Research Methodology* 2014;**14**:43. doi:10.1186/1471-2288-14-43

12 Wu C, Chen X, Cai Y, *et al.* Risk Factors Associated With Acute Respiratory Distress Syndrome and Death in Patients With Coronavirus Disease 2019 Pneumonia in Wuhan, China. *JAMA Internal Medicine* Published Online First: 13 March 2020. doi:10.1001/jamainternmed.2020.0994

13 Gorbalenya AE, Baker SC, Baric RS, *et al.* Severe acute respiratory syndrome-related coronavirus: The species and its viruses-a statement of the Coronavirus Study Group.

doi:10.1101/2020.02.07.937862
14 OpenSAFELY Collaborative T, Williamson E, Walker AJ, *et al.* OpenSAFELY: factors associated with COVID-19-related hospital death in the linked electronic health records of 17 million adult NHS patients. doi:10.1101/2020.05.06.20092999
15 Sommerstein R, Kochen MM, Messerli FH, *et al.* Coronavirus Disease 2019 (COVID-19): Do Angiotensin-Converting Enzyme Inhibitors/Angiotensin Receptor Blockers Have a Biphasic Effect? *Journal of the American Heart Association* 2020;**9**. doi:10.1161/jaha.120.016509
16 Chen Y, Gong X, Wang L, *et al.* Effects of hypertension, diabetes and coronary heart disease on COVID-19 diseases severity: a systematic review and meta-analysis. *medRxiv* 2020;:2020.03.25.20043133. doi:10.1101/2020.03.25.20043133
17 BAME COVID-19 DEATHS - What do we know? Rapid Data & Evidence Review - CEBM. https://www.cebm.net/covid-19/bame-covid-19-deaths-what-do-we-know-rapid-data-evidence-review/ (accessed 6 May 2020).
18 Zhou F, Yu T, Du R, *et al.* Clinical course and risk factors for mortality of adult inpatients with COVID-19 in Wuhan, China: a retrospective cohort study. *The Lancet* 2020;**395**:1054–62. doi:10.1016/S0140-6736(20)30566-3
19 Iacobellis G. COVID-19 and diabetes: Can DPP4 inhibition play a role? Published Online First: 2020. doi:10.1016/j.diabres.2020.108125
20 Úri K, Fagyas M, Siket IM, *et al.* New perspectives in the renin-angiotensin-aldosterone system (RAAS) IV: Circulating ACE2 as a biomarker of systolic dysfunction in human hypertension and heart failure. *PLoS ONE* 2014;**9**. doi:10.1371/journal.pone.0087845
21 Angiotensin-converting enzyme-2 overexpression improves atrial remodeling and function in a canine model of atrial fibrillation. *J Am Heart Assoc*;**4**:1–9.
22 Fernández-Atucha A, Izagirre A, Fraile-Bermúdez AB, *et al.* Sex differences in the aging pattern of renin-angiotensin system serum peptidases. *Biology of Sex Differences* 2017;**8**:5. doi:10.1186/s13293-017-0128-8
23 Deng S-Q, Peng H-J. Characteristics of and Public Health Responses to the Coronavirus Disease 2019 Outbreak in China. *Journal of Clinical Medicine* 2020;**9**:575. doi:10.3390/jcm9020575
24 Murad MH, Chu H, Lin L, *et al.* The effect of publication bias magnitude and direction on the certainty in evidence. *BMJ evidence-based medicine* 2018;**23**:84–6. doi:10.1136/bmjebm-2018-110891
25 Conradi U, Joffe AR. Publication bias in animal research presented at the 2008 Society of Critical Care Medicine Conference. *BMC Research Notes* 2017;**10**:262. doi:10.1186/s13104-017-2574-0
26 Zhen Y, Xin Y, Wenping J. GW24-e1144 Effects of enalapril on the expression of cardiac angiotensin-converting enzyme and angiotensin-converting enzyme 2 in spontaneously hypertensive rats. *Heart* 2013;**99**:A190.1-A190. doi:10.1136/heartjnl-2013-304613.529
27 Azis NA, Agarwal R, Ismail NM, *et al.* Blood pressure lowering effect of Ficus deltoidea var kunstleri in spontaneously hypertensive rats: possible involvement of renin-angiotensin-aldosterone system, endothelial function and anti-oxidant system. *Molecular biology reports* 2019;**46**:2841–9. doi:10.1007/s11033-019-04730-w
28 Hao X-Q, Zhang S-Y, Cheng X-C, *et al.* Imidapril inhibits right ventricular remodeling induced by low ambient temperature in broiler chickens. *Poultry Science* 2013;**92**:1492–7. doi:http://dx.doi.org/10.3382/ps.2012-02671
29 Jessup JA, Gallagher PE, Averill DB, *et al.* Effect of angiotensin II blockade on a new congenic model of hypertension derived from transgenic Ren-2 rats. *American journal of physiology Heart and circulatory physiology* 2006;**291**:H2166-72. doi:10.1152/ajpheart.00061.2006
30 Huang M, Li X, Meng Y, *et al.* Upregulation of angiotensin-converting enzyme (ACE) 2 in hepatic fibrosis by ACE inhibitors. *Clinical and Experimental Pharmacology and Physiology* 2010;**37**:e1–6. doi:http://dx.doi.org/10.1111/j.1440-1681.2009.05302.x
31 Wang G, Zhang Q, Yuan W, *et al.* Enalapril protects against myocardial ischemia/reperfusion injury in a swine model of cardiac arrest and resuscitation. *International journal of molecular medicine* 2016;**38**:1463–73. doi:10.3892/ijmm.2016.2737
32 Wang Y, Li C, Ouyang Y, *et al.* Cardioprotective Effects of Qishenyiqi Mediated by Angiotensin II Type 1 Receptor Blockade and Enhancing Angiotensin-Converting Enzyme 2. *Evidence-based Complementary and Alternative Medicine* 2012;**2012**:978127. doi:http://dx.doi.org/10.1155/2012/978127
33 Burchill L, Velkoska E, Dean RG, *et al.* Acute kidney injury in the rat causes cardiac remodelling and increases angiotensin-converting enzyme 2 expression. *Experimental*

For peer review only

physiology 2008;**93**:622–30. doi:10.1113/expphysiol.2007.040386
34 Zhang Y, Wang B, Wu J, *et al.* Role of ACE inhibitor and AT1 blockade on cardiac ACE and ACE2 expression in mice with unilateral ureteral ligation. *International Journal of Cardiology* 2011;**152**:S97. doi:10.1016/j.ijcard.2011.08.788
35 Zhang Y, Li B, Ma L, *et al.* GW25-e0791 Role of AT1 blockade on cardiac ACE2 and mas expression in hypertensive rats. *Journal of the American College of Cardiology* 2014;**64**:C179. doi:10.1016/j.jacc.2014.06.825
36 Bernardi S, Toffoli B, Zennaro C, *et al.* Aldosterone effects on glomerular structure and function. *Journal of the renin-angiotensin-aldosterone system : JRAAS* 2015;**16**:730–8. doi:10.1177/1470320315595568
37 Dong D, Fan T-T, Ji Y-S, *et al.* Spironolactone alleviates diabetic nephropathy through promoting autophagy in podocytes. *International urology and nephrology* 2019;**51**:755–64. doi:http://dx.doi.org/10.1007/s11255-019-02074-9
38 Kong E liang, Zhang J min, An N, *et al.* Spironolactone rescues renal dysfunction in obstructive jaundice rats by upregulating ACE2 expression. *Journal of Cell Communication and Signaling* 2019;**13**:17–26. doi:10.1007/s12079-018-0466-2
39 Hiroi N, Yoshihara A SM et al. Effects of olmesartan to the ACE2/Ang-(1-7)/Mas receptor endocrine society axis on essential hypertension patients. *Endocrine Reviews* 2014;**35**.http://press.endocrine.org/doi/abs/10.1210/endo-meetings.2014.CE.8.MON-0868%0Ahttp://ovidsp.ovid.com/ovidweb.cgi?T=JS&PAGE=reference&D=emed15&NEWS=N&AN=72338547
40 Li YQ, Wu JJ, Gao DF, *et al.* The impact of telmisartan on angiotensin converting enzyme 2 mRNA expression in monocyte-derived macrophages of diabetic hypertensive patients. *Zhonghua nei ke za zhi [Chinese journal of internal medicine]* 2013;**52**:26-29.
41 Wang X, Ye Y, Gong H, *et al.* The effects of different angiotensin II type 1 receptor blockers on the regulation of the ACE-AngII-AT1 and ACE2-Ang(1-7)-Mas axes in pressure overload-induced cardiac remodeling in male mice. *Journal of molecular and cellular cardiology* 2016;**97**:180–90. doi:10.1016/j.yjmcc.2016.05.012
42 Soler MJ, Ye M, Wysocki J, *et al.* Localization of ACE2 in the renal vasculature: Amplification by angiotensin II type 1 receptor blockade using telmisartan. *American journal of physiology Renal physiology* 2009;**296**:F398–405. doi:10.1152/ajprenal.90488.2008
43 Senador D, Key M, Brosnihan KB, *et al.* Cardiovascular interactions between losartan and fructose in mice. *Journal of cardiovascular pharmacology and therapeutics* 2010;**15**:68–77. doi:http://dx.doi.org/10.1177/1074248409351409
44 Abdelkader NF, Abd El-Latif AM, Khattab MM. Telmisartan/17beta-estradiol mitigated cognitive deficit in an ovariectomized rat model of Alzheimer's disease: Modulation of ACE1/ACE2 and AT1/AT2 ratio. *Life sciences* 2020;**245**:117388. doi:10.1016/j.lfs.2020.117388
45 Li Y, Cai S, Wang Q, *et al.* Valsartan attenuates intimal hyperplasia in balloon-injured rat aortic arteries through modulating the angiotensin-converting enzyme 2-angiotensin-(1-7)-Mas receptor axis. *Archives of biochemistry and biophysics* 2016;**598**:11–7. doi:http://dx.doi.org/10.1016/j.abb.2016.03.028
46 Zhang Y, Li B, Wang B, *et al.* Alteration of cardiac ACE2/Mas expression and cardiac remodelling in rats with aortic constriction. *The Chinese journal of physiology* 2014;**57**:335–42. doi:10.4077/CJP.2014.BAD268
47 Ji K, Minakawa M, Fukui K, *et al.* Olmesartan improves left ventricular function in pressure-overload hypertrophied rat heart by blocking angiotensin II receptor with synergic effects of upregulation of angiotensin converting enzyme 2. *Therapeutic advances in cardiovascular disease* 2009;**3**:103–11. doi:10.1177/1753944708098691
48 Sabry MM, Mahmoud MM, Shoukry HS, *et al.* Interactive effects of apelin, renin-angiotensin system and nitric oxide in treatment of obesity-induced type 2 diabetes mellitus in male albino rats. *Archives of physiology and biochemistry* 2019;**125**:244–54. doi:10.1080/13813455.2018.1453521
49 Li C-M, Zhang X-H, Zhang Y-Y, *et al.* The effect of Irbesartan on myocardial interstitial fibrosis in diabetic rats. *AFRICAN JOURNAL OF PHARMACY AND PHARMACOLOGY* 2011;**5**:1360–4. doi:10.5897/AJPP11.177
50 Abdel-Fattah MM, Messiha BAS, Mansour AM. Modulation of brain ACE and ACE2 may be a promising protective strategy against cerebral ischemia/reperfusion injury: an experimental trial in rats. *Naunyn-Schmiedeberg's Archives of Pharmacology* 2018;**391**:1003–20. doi:10.1007/s00210-018-1523-3
51 Arumugam S, Thandavarayan RA, Palaniyandi SS, *et al.* Candesartan cilexetil protects from

For peer review only

cardiac myosin induced cardiotoxicity via reduction of endoplasmic reticulum stress and apoptosis in rats: involvement of ACE2-Ang (1-7)-mas axis. *Toxicology* 2012;**291**:139–45. doi:10.1016/j.tox.2011.11.008

52 Kidoguchi S, Sugano N, Takane K, *et al.* Azilsartan causes natriuresis due to its sympatholytic action in kidney disease. *Hypertension research : official journal of the Japanese Society of Hypertension* 2019;**42**:1507–17. doi:10.1038/s41440-019-0271-1

53 Liang B, Li Y, Han Z, *et al.* ACE2-Ang (1-7) axis is induced in pressure overloaded rat model. *International journal of clinical and experimental pathology* 2015;**8**:1443–50.

54 Awwad ZM, El-Ganainy SO, ElMallah AI, *et al.* Telmisartan and captopril ameliorate pregabalin-induced heart failure in rats. *Toxicology* 2019;**428**:152310. doi:10.1016/j.tox.2019.152310

55 Sukumaran V, Veeraveedu PT, Gurusamy N, *et al.* Telmisartan acts through the modulation of ACE-2/ANG 1-7/mas receptor in rats with dilated cardiomyopathy induced by experimental autoimmune myocarditis. *Life sciences* 2012;**90**:289–300. doi:10.1016/j.lfs.2011.11.018

56 Yang D, Lin Z, Ni Y, *et al.* A1059 Telmisartan upregulates ACE2-Ang-(1–7)-Mas axis and affects retinal vessel endothelial cell apoptosis. *Journal of Hypertension* 2018;**36**:e11. doi:10.1097/01.hjh.0000548029.92325.e5

57 Takai S, Jin D, Aritomi S, *et al.* Powerful vascular protection by combining cilnidipine with valsartan in stroke-prone, spontaneously hypertensive rats. *Hypertension research : official journal of the Japanese Society of Hypertension* 2013;**36**:342–8. doi:10.1038/hr.2012.187

58 Zhong J-C, Ye J-Y, Jin H-Y, *et al.* Telmisartan attenuates aortic hypertrophy in hypertensive rats by the modulation of ACE2 and profilin-1 expression. *Regulatory peptides* 2011;**166**:90–7. doi:10.1016/j.regpep.2010.09.005

59 Zhao Y, Ma R, Yu X, *et al.* AHU377+Valsartan (LCZ696) Modulates Renin-Angiotensin System (RAS) in the Cardiac of Female Spontaneously Hypertensive Rats Compared With Valsartan. *Journal of cardiovascular pharmacology and therapeutics* 2019;**24**:450–9. doi:10.1177/1074248419838503

60 Agata J, Ura N, Yoshida H, *et al.* Olmesartan is an angiotensin II receptor blocker with an inhibitory effect on angiotensin-converting enzyme. *Hypertension research : official journal of the Japanese Society of Hypertension* 2006;**29**:865–74. doi:10.1291/hypres.29.865

61 Igase M, Kohara K, Nagai T, *et al.* Increased expression of angiotensin converting enzyme 2 in conjunction with reduction of neointima by angiotensin II type 1 receptor blockade. *Hypertension research : official journal of the Japanese Society of Hypertension* 2008;**31**:553–9. doi:10.1291/hypres.31.553

62 Igase M, Strawn WB, Gallagher PE, *et al.* Angiotensin II AT1 receptors regulate ACE2 and angiotensin-(1-7) expression in the aorta of spontaneously hypertensive rats. *American journal of physiology Heart and circulatory physiology* 2005;**289**:H1013-9. doi:10.1152/ajpheart.00068.2005

63 Wosten-van Asperen RM, Lutter R, Specht PA, *et al.* Acute respiratory distress syndrome leads to reduced ratio of ACE/ACE2 activities and is prevented by angiotensin-(1-7) or an angiotensin II receptor antagonist. *The Journal of pathology* 2011;**225**:618–27. doi:10.1002/path.2987

64 Ohshima K, Mogi M, Nakaoka H, *et al.* Possible role of angiotensin-converting enzyme 2 and activation of angiotensin II type 2 receptor by angiotensin-(1-7) in improvement of vascular remodeling by angiotensin II type 1 receptor blockade. *Hypertension (Dallas, Tex : 1979)* 2014;**63**:e53-9. doi:10.1161/HYPERTENSIONAHA.113.02426

65 Gallagher PE, Ferrario CM, Tallant EA. Regulation of ACE2 in cardiac myocytes and fibroblasts. *American journal of physiology Heart and circulatory physiology* 2008;**295**:H2373-9. doi:10.1152/ajpheart.00426.2008

66 Feng P, Wu Z, Liu H, *et al.* Electroacupuncture Improved Chronic Cerebral Hypoperfusion-Induced Anxiety-Like Behavior and Memory Impairments in Spontaneously Hypertensive Rats by Downregulating the ACE/Ang II/AT1R Axis and Upregulating the ACE2/Ang-(1-7)/MasR Axis. *Neural plasticity* 2020;**2020**:9076042. doi:10.1155/2020/9076042

67 Song R, Preston G, Yosypiv I V. Ontogeny of angiotensin-converting enzyme 2. *Pediatric research* 2012;**71**:13–9. doi:10.1038/pr.2011.7

68 Lezama-Martinez D, Flores-Monroy J, Fonseca-Coronado S, *et al.* Combined antihypertensive therapies that increase expression of cardioprotective biomarkers associated with the renin-angiotensin and kallikrein-kinin system. *Journal of Cardiovascular Pharmacology* 2018;**72**:291–5. doi:10.1097/FJC.0000000000000629

69 Abe M, Oikawa O, Okada K, *et al.* Urinary angiotensin-converting enzyme 2 increases in

For peer review only

diabetic nephropathy by angiotensin II type 1 receptor blocker olmesartan. *Journal of the renin-angiotensin-aldosterone system : JRAAS* 2015;**16**:159–64. doi:http://dx.doi.org/10.1177/1470320314551443

70 K, Shimada KK, Kinouchi T, Yagi K TY, Satomi J KJ. International Stroke Conference and Nursing Symposium Poster Presentations. *Stroke* 2011;**42**:e149. doi:10.1161/str.0b013e3182074d9b

71 Ishiyama Y, Gallagher PE, Averill DB, *et al.* Upregulation of angiotensin-converting enzyme 2 after myocardial infarction by blockade of angiotensin II receptors. *Hypertension (Dallas, Tex : 1979)* 2004;**43**:970–6. doi:10.1161/01.HYP.0000124667.34652.1a

72 Guo J, Lu W, Wang X, *et al.* Protective effects of telmisartan in a rat model of pulmonary arterial hypertension. *Experimental and Clinical Cardiology* 2014;**20**:2429–37.

73 Yisireyili M, Uchida Y, Yamamoto K, *et al.* Angiotensin receptor blocker irbesartan reduces stress-induced intestinal inflammation via AT1a signaling and ACE2-dependent mechanism in mice. *Brain, behavior, and immunity* 2018;**69**:167–79. doi:10.1016/j.bbi.2017.11.010

74 Suh SH, Choi HS, Kim CS, *et al.* Olmesartan Attenuates Kidney Fibrosis in a Murine Model of Alport Syndrome by Suppressing Tubular Expression of TGFbeta. *International journal of molecular sciences* 2019;**20**. doi:10.3390/ijms20153843

75 Iwanami j, Mogi M TK et al. Role of angiotensin converting enzyme 2/angiotensin (1-7)/Mas axis in the hypotensive effect of azilsartan. *Hypertension* 2013;**62**.

76 G.-H. Y. The strategy of rennin-angiotensin system blockers reduce the indoxyl sulfate-mediated renal damage on the duration of acute kidney injury. *Nephrology* 2014;**19**:161–2. doi:http://dx.doi.org/10.1111/nep.12237

77 Ichikawa H, Narita I, Narita M, *et al.* Blood pressure-independent effect of olmesartan on albuminuria in mice overexpressing renin: Its beneficial role in the ACE2/ang (1-7)/mas axis and NADPH oxidase expression. *International Heart Journal* 2018;**59**:1445–53. doi:10.1536/ihj.17-582

78 Nakaoka H, Mogi M, Kan-no H, *et al.* Effect of Olmesartan on Glucose Metabolism Involving Angiotensin Converting Enzyme 2. *Immunology, Endocrine & Metabolic Agents in Medicinal Chemistry* 2017;**17**:105–14. doi:10.2174/1871522217666171019150511

79 Varagic J, Ahmad S, Voncannon JL, *et al.* Predominance of AT1 blockade over mas-mediated angiotensin-(1-7) mechanisms in the regulation of blood pressure and renin-angiotensin system in mRen2.Lewis rats. *American Journal of Hypertension* 2013;**26**:583–90. doi:10.1093/ajh/hps090

80 de Araujo AA, Araujo L de S, Carvalho CA, *et al.* Protective effect of angiotensin II receptor blocker against oxidative stress and inflammation in an oral mucositis experimental model. *JOURNAL OF ORAL PATHOLOGY & MEDICINE* 2018;**47**:972–84. doi:10.1111/jop.12775

81 Zhang YH, Hao QQ, Wang XY, *et al.* ACE2 activity was increased in atherosclerotic plaque by losartan: Possible relation to anti-atherosclerosis. *Journal of the renin-angiotensin-aldosterone system : JRAAS* 2015;**16**:292–300. doi:10.1177/1470320314542829

82 Wu J, Chen T, Zhang Y. GW26-e0735 Cardiac ace2/mas expression and cardiac remodeling in hypertensive rats. *Journal of the American College of Cardiology* 2015;**66**:C76. doi:10.1016/j.jacc.2015.06.304

83 Chen L, Yu M ZC et al., M. Alterations of urinary angiotensin converting enzyme and ACE2 before and after perindopril medication in patients with diabetic kidney disease. *Diabetes/Metabolism Research and Reviews* 2015;**31**:51–83. doi:10.1002/dmrr.2728

84 Graus-Nunes F, Santos F de O, Marinho T de S, *et al.* Beneficial effects of losartan or telmisartan on the local hepatic renin-angiotensin system to counter obesity in an experimental model. *World journal of hepatology* 2019;**11**:359–69. doi:10.4254/wjh.v11.i4.359

85 Tanno T, Tomita H, Narita I, *et al.* Olmesartan Inhibits Cardiac Hypertrophy in Mice Overexpressing Renin Independently of Blood Pressure: Its Beneficial Effects on ACE2/Ang(1-7)/Mas Axis and NADPH Oxidase Expression. *Journal of cardiovascular pharmacology* 2016;**67**:503–9. doi:10.1097/FJC.0000000000000374

86 Ishibashi Y, Matsui T, Yamagishi S, *et al.* Olmesartan blocks advanced glycation end products-induced vcam-1 gene expression in mesangial cells by restoring Angiotensin-converting enzyme 2 level. *Hormone and Metabolic Research* 2014;**46**:379–83. doi:10.1055/s-0033-1361114

87 Wang W, Song A, Zeng Y, *et al.* Telmisartan protects chronic intermittent hypoxic mice via modulating cardiac renin-angiotensin system activity. *BMC Cardiovascular Disorders* 2018;**18**. doi:10.1186/s12872-018-0875-4

88 Malek V, Sharma N, Sankrityayan H, *et al.* Concurrent neprilysin inhibition and renin-

For peer review only - http://bmjopen.bmj.com/site/about/guidelines.xhtml

angiotensin system modulations prevented diabetic nephropathy. *Life Sciences* 2019;**221**:159–67. doi:10.1016/j.lfs.2019.02.027

89 Varagic J, Ahmad S, Voncannon JL, *et al.* Nebivolol reduces cardiac angiotensin II, associated oxidative stress and fibrosis but not arterial pressure in salt-loaded spontaneously hypertensive rats. *Journal of hypertension* 2012;**30**:1766–74. doi:10.1097/HJH.0b013e328356766f

90 Iizuka K, Kusunoki A, Machida T, *et al.* Angiotensin II reduces membranous angiotensin-converting enzyme 2 in pressurized human aortic endothelial cells. *Journal of the renin-angiotensin-aldosterone system : JRAAS* 2009;**10**:210–5. doi:10.1177/1470320309343710

91 Onat E. Effects of rosuvastatin and amlodipine on reninangiotensin system of kidney in NOS inhibition and salt diet induced hypertension. *Journal of Cellular Neuroscience and Oxidative Stress* 2018;**10**:693–4.http://dergipark.gov.tr/download/article-file/477202%0Ahttp://ovidsp.ovid.com/ovidweb.cgi?T=JS&PAGE=reference&D=emed19&NEWS=N&AN=625308440

92 Zhang L-H, Pang X-F, Bai F, *et al.* Preservation of Glucagon-Like Peptide-1 Level Attenuates Angiotensin II-Induced Tissue Fibrosis by Altering AT1/AT 2 Receptor Expression and Angiotensin-Converting Enzyme 2 Activity in Rat Heart. *Cardiovascular drugs and therapy* 2015;**29**:243–55. doi:10.1007/s10557-015-6592-7

93 Awwad ZM, El-Ganainy SO, ElMallah AI, *et al.* Assessment of Pregabalin-Induced Cardiotoxicity in Rats: Mechanistic Role of Angiotensin 1-7. *Cardiovascular toxicology* Published Online First: November 2019. doi:10.1007/s12012-019-09553-6

94 Romani-Perez M, Outeirino-Iglesias V, Moya CM, *et al.* Activation of the GLP-1 Receptor by Liraglutide Increases ACE2 Expression, Reversing Right Ventricle Hypertrophy, and Improving the Production of SP-A and SP-B in the Lungs of Type 1 Diabetes Rats. *Endocrinology* 2015;**156**:3559–69. doi:10.1210/en.2014-1685

95 Riera M, Marquez E, Clotet S, *et al.* Effect of insulin on ACE2 activity and kidney function in the non-obese diabetic mouse. *PloS one* 2014;**9**:e84683. doi:10.1371/journal.pone.0084683

96 Marquez E, Riera M, Pascual J, *et al.* Albumin inhibits the insulin-mediated ACE2 increase in cultured podocytes. *American journal of physiology Renal physiology* 2014;**306**:F1327-34. doi:10.1152/ajprenal.00594.2013

97 Shin YH, Min JJ, Lee J-H, *et al.* The effect of fluvastatin on cardiac fibrosis and angiotensin-converting enzyme-2 expression in glucose-controlled diabetic rat hearts. *Heart and vessels* 2017;**32**:618–27. doi:10.1007/s00380-016-0936-5

98 Salem ES, Chodavarapu H, Morris M, *et al.* Insulin normalizes angiotensin converting enzyme 2 (ACE2) and attenuates albuminuria in type 1 diabetic Akita mice. Hypertension. 2012;**60**.

99 Salem E SH. Acute and Chronic Complications. *Diabetes* 2013;**62**:A588–621. doi:10.2337/db13-2297-2440

100 Salem ESB, Grobe N, Elased KM. Insulin treatment attenuates renal ADAM17 and ACE2 shedding in diabetic Akita mice. *American journal of physiology Renal physiology* 2014;**306**:F629-39. doi:10.1152/ajprenal.00516.2013

101 Blanco-Gozalo V, Blazquez-Medela A, Garcia-Sanchez O, *et al.* Diabetes - experimental models. *Nephrology Dialysis Transplantation* 2013;**28**:i374–84. doi:10.1093/ndt/gft137

102 Min JJ, Shin B-S, Lee J-H, *et al.* Effects of pravastatin on type 1 diabetic rat heart with or without blood glycemic control. *Journal of Diabetes Research* 2018;**2018**:1067853. doi:http://dx.doi.org/10.1155/2018/1067853

103 Gupta RC, Want G RS et al. Long-term therapy with ivabradine increases ace-2 activity in left ventricular myocardium of dogs with chronic heart failure. *Circulation Research* 2012;**111**.http://ovidsp.ovid.com/ovidweb.cgi?T=JS&PAGE=reference&D=emed13&NEWS=N&AN=71053143

104 Weili Q, Cheng W, Fan Z, *et al.* GW25-e4430 Ibuprofen Attenuates Cardiac Fibrosis via Restoring the Imbalance of ACE and ACE2 in Diabetic Rat. *Journal of the American College of Cardiology* 2014;**64**:C62. doi:10.1016/j.jacc.2014.06.297

105 Qiao W, Wang C, Chen B, *et al.* Ibuprofen attenuates cardiac fibrosis in streptozotocin-induced diabetic rats. *Cardiology* 2015;**131**:97–106. doi:10.1159/000375362

106 Bukowska A, Spiller L, Wolke C, *et al.* Protective regulation of the ACE2/ACE gene expression by estrogen in human atrial tissue from elderly men. *Experimental Biology and Medicine* 2017;**242**:1412–23. doi:10.1177/1535370217718808

107 Hermenegildo C, Mompeon A, Perez-Cremades D, *et al.* [OP.6D.01] ESTRADIOL INCREASES ENDOTHELIAL ANGIOTENSIN-(1-7) PRODUCTION THROUGH ESTROGEN RECEPTOR ALPHA. *Journal of Hypertension* 2016;**34**:e75.

For peer review only

doi:10.1097/01.hjh.0000491530.65088.bb
108 Mompeon A, Lazaro-Franco M, Bueno-Beti C, *et al.* Estradiol, acting through ERalpha, induces endothelial non-classic renin-angiotensin system increasing angiotensin 1-7 production. *Molecular and Cellular Endocrinology* 2016;**422**:1–8. doi:http://dx.doi.org/10.1016/j.mce.2015.11.004
109 Shenoy V, Grobe JL, Qi Y, *et al.* 17beta-Estradiol modulates local cardiac renin-angiotensin system to prevent cardiac remodeling in the DOCA-salt model of hypertension in rats. *Peptides* 2009;**30**:2309–15. doi:10.1016/j.peptides.2009.09.005
110 Wang H, Zhao Z JJ et al. Estrogen replacement downregulates cardiac angiotensin ii in an ace-independent manner in oophorectomized mRen2.Lewis rats. *Circulation Research* 2013;**113**.http://ovidsp.ovid.com/ovidweb.cgi?T=JS&PAGE=reference&D=emed14&NEWS=N&AN=71323938
111 Wang G, Zhang Q, Yuan W, *et al.* Sildenafil protects against myocardial ischemia-reperfusion injury following cardiac arrest in a porcine model: Possible role of the renin-angiotensin system. *International Journal of Molecular Sciences* 2015;**16**:27015–31. doi:http://dx.doi.org/10.3390/ijms161126010
112 Thanekar U, Gill RK, Dhakal S, *et al.* Abstract P2044: Renoprotective Effects of Canagliflozin in Db/db Diabetic Mice. *Hypertension* 2019;**74**. doi:10.1161/hyp.74.suppl_1.p2044
113 Li Y-H, Wang Q-X, Zhou J-W, *et al.* Effects of rosuvastatin on expression of angiotensin-converting enzyme 2 after vascular balloon injury in rats. *Journal of geriatric cardiology : JGC* 2013;**10**:151–8. doi:10.3969/j.issn.1671-5411.2013.02.009
114 A. G, M. S, R. O, *et al.* Influence of atorvastatin on angiotensin I metabolism in rat vascular smooth muscle cells. *Pharmacological Reports* 2013;**65**:47.
115 Aguilar C, Ventura F, Rodríguez-Delfín L. Atorvastatin induced increase in homologous angiotensin I converting enzyme (ACE2) mRNA Is associated to decreased fibrosis and decreased left ventricular hypertrophy in a rat model of diabetic cardiomyopathy. *Revista Peruana de Medicina Experimental y Salud Publica* 2011;**28**:264–72. doi:10.1590/S1726-46342011000200013
116 Jessup JA, Brosnihan KB, Gallagher PE, *et al.* Differential effect of low dose thiazides on the Renin Angiotensin system in genetically hypertensive and normotensive rats. *Journal of the American Society of Hypertension : JASH* 2008;**2**:106–15. doi:10.1016/j.jash.2007.10.005
117 Sanchez-Aguilar M, Ibarra-Lara L, Del Valle-Mondragon L, *et al.* Rosiglitazone, a Ligand to PPAR gamma, Improves Blood Pressure and Vascular Function through Renin-Angiotensin System Regulation. *PPAR Research* 2019;**2019**:1371758. doi:http://dx.doi.org/10.1155/2019/1371758
118 Scroggin M PK. The PPAR-{gamma} agonist Rosiglitazone increases Angiotensin-Converting Enzyme 2 (ACE2) promoter activity in neurons. *FASEB Journal* 2012;**26**.http://www.fasebj.org/cgi/content/meeting_abstract/26/1_MeetingAbstracts/875.13?sid=9bbf00be-78fb-4079-afb1-341158d7edc8%0Ahttp://ovidsp.ovid.com/ovidweb.cgi?T=JS&PAGE=reference&D=emed13&NEWS=N&AN=70857313
119 Zhang W, Li C, Liu B, *et al.* Pioglitazone upregulates hepatic angiotensin converting enzyme 2 expression in rats with steatohepatitis. *Annals of hepatology* 2013;**12**:892–900.
120 Aguilar MS, Aguilar-Navarro A, Ibarra-Lara L, *et al.* PPAR Ganna stimulation by rosiglitazone decreases blood pressure and renal apoptosis in a rat hypertension model secondary to aortic coartaction. *Journal of Hypertension* 2018;**36**:e43. doi:10.1097/01.hjh.0000539075.61733.23
121 Chodavarapu H, Grobe N, Somineni HK, *et al.* Rosiglitazone Treatment of Type 2 Diabetic db/db Mice Attenuates Urinary Albumin and Angiotensin Converting Enzyme 2 Excretion. *PLoS ONE* 2013;**8**:e62833. doi:10.1371/journal.pone.0062833
122 Ali RM, Al-Shorbagy MY, Helmy MW, *et al.* Role of Wnt4/beta-catenin, Ang II/TGFbeta, ACE2, NF-kappaB, and IL-18 in attenuating renal ischemia/reperfusion-induced injury in rats treated with Vit D and pioglitazone. *European Journal of Pharmacology* 2018;**831**:68–76. doi:http://dx.doi.org/10.1016/j.ejphar.2018.04.032
123 Andersen LB, Przybyl L, Haase N, *et al.* Vitamin D depletion aggravates hypertension and target-organ damage. *Journal of the American Heart Association* 2015;**4**:e001417. doi:http://dx.doi.org/10.1161/JAHA.114.001417
124 da Silva Machado C, Alexandre FH C de ML et al. Vitamin D3 regulates genes involved with the renin-angiotensin-aldosterone system in hypertensive and normotensive rats. *Toxicology Letters* 2014;**229**:S231. doi:10.1016/j.toxlet.2014.06.773
125 Lin M, Gao P, Zhao T, *et al.* Calcitriol regulates angiotensin-converting enzyme and

For peer review only

angiotensin converting-enzyme 2 in diabetic kidney disease. *Molecular biology reports* 2016;**43**:397–406. doi:10.1007/s11033-016-3971-5

126 Xu J, Yang J, Chen J, *et al.* Vitamin D alleviates lipopolysaccharideinduced acute lung injury via regulation of the reninangiotensin system. *Molecular medicine reports* 2017;**16**:7432–8. doi:10.3892/mmr.2017.7546

127 Speth R, Carrera E J-BM et al. Concentration-dependent effects of zinc on angiotensin-converting enzyme-2 activity. *FASEB Journal* 2014;**28**.

Figure 1: PRISMA flow chart explaining the study inclusion process

[insert figure 1 here]

For peer review only

Table 1: Characteristics and key findings of included studies
*Studies reporting on multiple sites or in multiple models have been listed separately and appear more than once in the table

**Drug class**	**Sample size, median (range)**	**Exposure to treatment, mean (SD)**	**Effect on ACE2 expression, levels or activity (number of studies) ***	**Model tested (number of studies)***	**Site of ACE2 receptor measurement (number of studies)***	**Condition of subjects (number of studies)***
ACE inhibitors [26–35]	32 (7 to 375)	4 weeks (3)	Increase (n= 17)<br>Decrease (n= 1)<br>No effect (n= 4)	Rats, in vivo (n= 16)<br>Humans, in vivo (n= 2)<br>Other (n =4)	Cardiac (n= 14)<br>Hepatic (n= 2)<br>Renal (n= 2)<br>Not stated (n= 2)<br>Other (n= 3)	Heart disease/heart failure (n= 7)<br>Hypertension (n= 3)<br>Diabetes (n= 3)<br>Healthy (n=2)<br>Other (n= 1)<br>Not stated (n= 6)
Aldosterone antagonists [36–38]	63 (28 to 75)	4 weeks (4)	Increase (n= 3)<br>Decrease (n= 0)<br>No effect (n= 0)	Rats, in vivo (n = 3)	Renal (n= 3)	Diabetes (n= 1)<br>Renal disease (n= 1)<br>Hepatic dysfunction (n= 1)
ARBs [27,29,34,35,39–88]	36 (6 to 180)	6 weeks (6)	Increase (n= 43)<br>Decrease (n= 7)<br>No effect (n= 3)<br>Unclear/mixed findings (n =2)	Rats, in vivo (n= 34)<br>Mice, in vivo (n = 11)<br>Humans - in vivo (n= 4)<br>Other (n= 6)	Cardiac (n= 27)<br>Hepatic (n= 2)<br>Renal (n= 12)<br>Lung (n = 2)<br>Not stated (n= 1)<br>Other (n= 7)	Hypertension (n= 13)<br>Heart disease/heart failure (n= 10)<br>Diabetes (n= 5)<br>Healthy (n=6)<br>Not stated (n= 13)<br>Other (n= 5)

For peer review only

Beta Blockers [62,89]	52 (44 to 62)	4 weeks (2)	Increase (n= 0)<br>Decrease (n= 0)<br>No effect (n= 2)	Rats, in vivo (n = 1)<br>Rats, in vitro (n = 1)	Cardiac (n= 2)	Hypertension (n= 2)
Calcium channel blockers [50,90,91]	117 (N/A)	3 weeks (1)	Increase (n= 2)<br>Decrease (n= 1)<br>No effect (n= 0)	Rats, in vivo (n = 2)<br>Human, in vitro (n = 1	Hepatic (n= 1)<br>Cardiac (n= 1)<br>Cerebral (n =1)	Healthy (n= 2)<br>Hypertension (n= 1)
Centrally-acting vasodilators [85]	6 (N/A)	8 weeks (N/A)	Increase (n= 0)<br>Decrease (n= 0)<br>No effect (n= 1)	Mice, in vitro (n= 1)	Cardiac (n= 1)	Other (n= 1)
DPP4 inhibitor [92]	24 (N/A)	4 weeks (N/A)	Increase (n= 1)<br>Decrease (n= 0)<br>No effect (n= 0)	Rats, in vivo (n = 1)	Cardiac (n= 1)	Healthy (n=1)

For peer review only

GABA analogues [93]	8 (N/A)	3 weeks (N/A)	Increase (n= 0)<br>Decrease (n= 1)<br>No effect (n= 0)	Rats, in vivo (n= 1)	Cerebral (n= 1)	Heathy (n= 1)
GLP-1 agonists [92,94]	38 (24 to 54)	3 weeks (1)	Increase (n= 2)<br>Decrease (n= 0)<br>No effect (n= 0)	Rats, in vivo (n = 2)	Cardiac (n= 1)<br>Lung (n= 1)	Diabetic (n=1)<br>Healthy (n=1)
Insulin [95–102]	57 (8 to 84)	6 weeks (6)	Increase (n= 6)<br>Decrease (n= 1)<br>No effect (n= 1)	Mice, in vivo (n = 4)<br>Mice, in vitro (n = 1)<br>Rats, in vivo (n = 2)<br>Rats, in vitro (n = 1)	Renal (n= 5)<br>Cardiac (n= 2)	Diabetes (n= 7)<br>Healthy (n= 1)
Ivabridine [103]	24 (N/A)	12 weeks (N/A)	Increase (n= 1)<br>Decrease (n= 0)<br>No effect (n= 0)	Canine, in vivo (n= 1)	Cardiac (n= 1)	Heart failure (n= 1)

For peer review only

NSAIDs [104,105]	18 (N/A)	8 weeks (0)	Increase (n= 2)<br>Decrease (n= 0)<br>No effect (n= 0)	Rats, in vivo (n = 2)	Cardiac (n= 2)	Diabetic (n=2)
Oestrogens [44,106–110]	27 (17 to 75)	3 weeks (3)	Increase (n= 3)<br>Decrease (n= 1)<br>No effect (n= 1)	Human, in vitro (n = 2)<br>Human, in vivo (n = 1)<br>Rats, in vivo (n = 2)<br>Mice, in vivo (n = 1)	Cardiac (n= 2)<br>Ovarian (n= 1)<br>Cerebral (n= 1)<br>Not stated (n= 2)	Heart disease (n= 1)<br>Hypertension (n= 1)<br>Hypertension + ovariectomy (n= 1)<br>Healthy (n= 1)<br>Not stated (n= 1)<br>Alzheimers (n=1)
PDE-5 inhibitors [111]	32	30 minutes (N/A)	Increase (n= 0)<br>Decrease (n= 0)<br>No effect (n= 1)	Rats, in vitro (n = 1)	Cardiac (n= 1)	Healthy (n=1)
SGLT2 inhibitors [112]	Not stated	15 weeks (N/A)	Increase (n= 0)<br>Decrease (n= 0)<br>No effect (n= 1)	Mice, in vitro (n= 1)	Renal (n= 1)	Diabetic (n= 1)

For peer review only

Statins [91,102,113–115]	62 (36 to 87)	5 weeks (3)	Increase (n= 3)<br>Decrease (n= 1)<br>No effect (n= 1)	Rats, in vivo (n = 4)<br>Rats, in vitro (n = 1)	Cardiac (n= 4)<br>Renal (n= 1)	Diabetes (n= 2)<br>Hypertension (n= 1)<br>Unclear (n= 1)<br>Unclear (n= 1)
Thiazide and thiazide-like diuretics [116]	48 (N/A)	1 week (N/A)	Increase (n= 1)<br>Decrease (n= 1)<br>No effect (n= 0)	Rats, in vivo (n = 1)	Cardiac (n= 1)	Hypertension (n=1)<br>Healthy (n=1)
Thiazolidinedione [104,117–122]	21 (8 to 60)	6 weeks (8)	Increase (n= 5)<br>Decrease (n= 1)<br>No effect (n= 1)	Rats, in vivo (n = 4)<br>Rats, in vitro (n = 1)<br>Human, in vitro (n = 1)<br>Mice, in vivo (n = 1)	Renal (n= 3)<br>Cardiac (n= 2)<br>Hepatic (n= 1)<br>Cerebral (n =1)	Hypertension (n= 1)<br>Heart disease/heart failure (n= 1)<br>Diabetes (n= 1)<br>Healthy (n=1)<br>Renal disease (n= 1)<br>Not stated (n= 2)
Vitamin D [122–124]	47 (33 to 60)	6 weeks (6)	Increase (n= 2)<br>Decrease (n= 0)<br>No effect (n= 1)	Rats, in vivo (n= 3)	Cardiac (n= 1)<br>Renal (n= 1)<br>Not stated (n= 0)	Hypertension (n= 1)<br>Renal disease (n= 1)<br>Not stated (n= 1)
Vitamin D analogues [125,126]	28 (25 to 30)	8 weeks (11)	Increase (n= 1)<br>Decrease (n= 0)<br>No effect (n= 1)	Rats, in vivo (n= 1)<br>Rats, in vitro (n= 1)	Renal (n= 1)<br>Lung (n = 1)	Diabetes (n= 5)<br>Lung injury (n=1)
Zinc [127]	Not stated	Not stated	Increase (n= 0)<br>Decrease (n= 1)<br>No effect (n= 0)	Rats, in vitro (n= 1)	Renal (n= 1)<br>Lung (n= 1)	Not stated (n= 1)

For peer review only

Table 2: Summary of studies characteristics with human models

Drug Class	Sample size, median (range)	Exposure to treatment, mean (SD)	Effect on ACE2 expression, levels or activity (number of studies)	Model tested	Site of ACE2 receptors	Condition of subject
Angiotensin-Receptor Blockers	46.5 (8 to 80)	15 weeks (6)	Increase (n= 2)<br>Decrease (n= 1)<br>No effect (n= 1)	In vivo (n= 3) In vitro (n= 1)	Urinary (n= 1)<br>Serum (n= 1)<br>Renal (n= 1)<br>Not stated (n= 1)	Diabetes (n= 1)<br>Hypertension (n= 1)<br>Hypertension + Diabetes (n= 1)<br>Diabetic + Chronic Kidney Disease (n= 1)
Angiotensin Converting Enzyme-Inhibitors	228 (80 to 375)	12 weeks (N/A)	Increase (n= 1)<br>Decrease (n= 1)	In vitro (n= 2)	Renal (n= 1)<br>Unclear (n= 1)	Diabetic + Chronic Kidney Disease (n= 1)<br>Unclear (n= 1)
Calcium Channel Blockers	N/A	Unclear	Increase ACE2 in the membrane surface (decreased in the cytosol) (n= 1)	In vitro (n= 1)	Cardiac	Healthy cells
Oestrogen	36 (N/A)	1 day (N/A)	Increase (n= 2)<br>No effect (n= 1)	In vitro (n= 3)	Cardiac (n= 1)<br>Umbilical (n= 1)<br>Not stated (n= 1)	Heart problems (n= 1)<br>Healthy (n= 1)<br>Not stated (n= 1)
Thiazolidinedione	Not stated	1 day	Increase (n= 1)	In vitro (n= 1)	Cerebral (n= 1)	Not stated

For peer review only

Figure 1: PRISMA flow chart explaining the study inclusion process

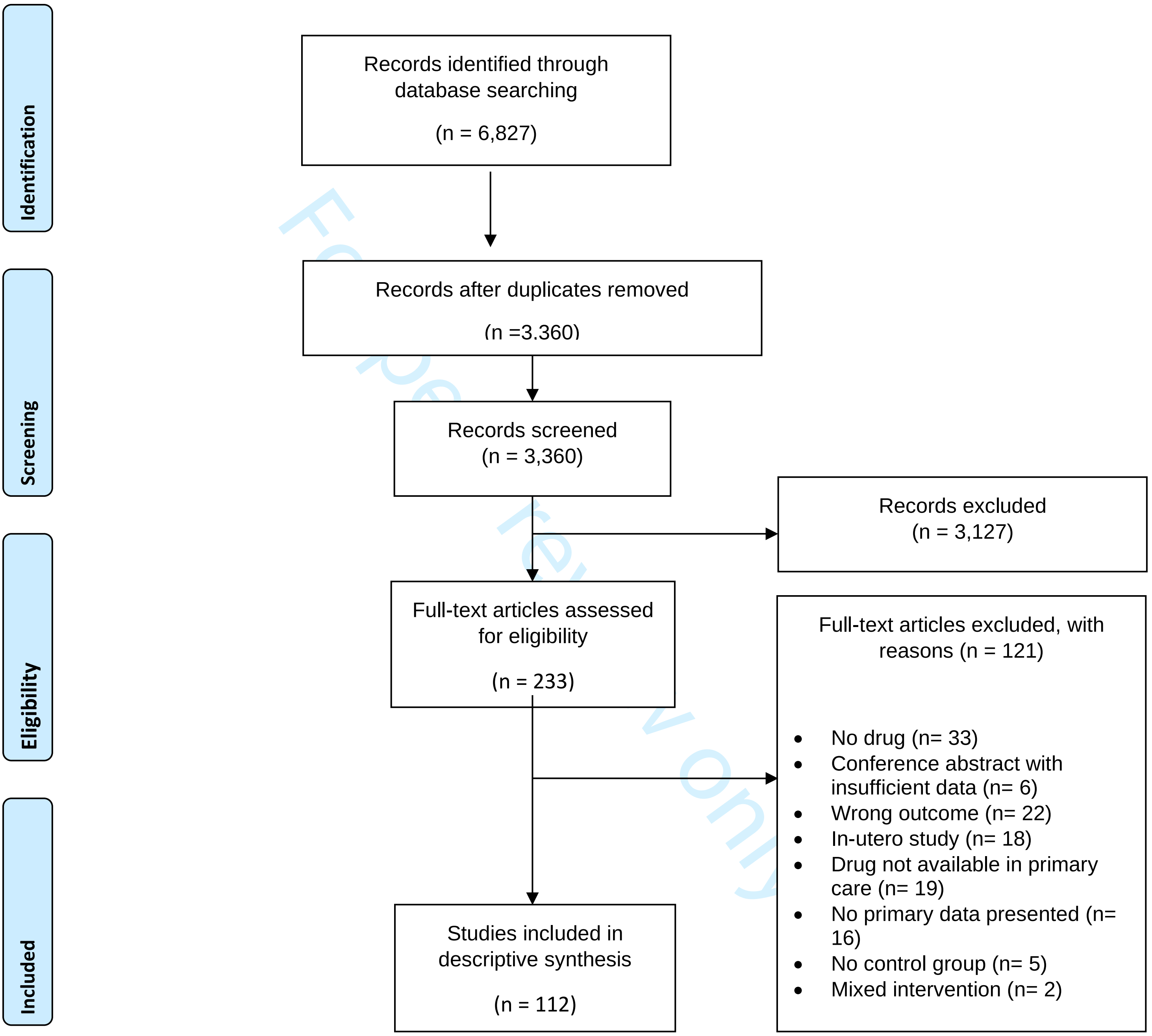

Supplementary 1: Summary of search strategy

Terms	Database	Number of articles
(ace2[Title/Abstract]) OR ace 2[Title/Abstract] Filters: English	Medline (PubMed)	1980
(ace2 or ace 2).ab. and English.lg.	Embase	2880
ace2 in Title Abstract Keyword OR ace-2 in Title Abstract Keyword - (Word variations have been searched)	Cochrane	35 trials and 1 review
(TS=(ace2 OR ace-2)) AND LANGUAGE: (English) AND DOCUMENT TYPES: (Article)	Web of science	1,931

For peer review only

Supplementary Material 2: Table of studies at baseline

**Study**	**Country**	**Exposure drug(s)**	**Duration of exposure**	**Subject / model tested**	**Site of ACE2 receptors measured**	**Sample size**	**Condition of subject / model**
Abdel-Fattah 2018	Egypt	Telmisartan or Nimodipine	15 days	Rat	Cerebral	117	Healthy
Abdelkader 2020	Egypt	Telmisartan and 17B-estradiol	6 weeks	Rat	Cerebral	75	Alzheimer's mouse model
Abe 2015	Japan	Olmesartan	24 weeks	Human	Renal	31	Diabetic
Abuohashish 2017	Egypt	Captopril	6 weeks	Rat	Bone	Not stated	Oestrogen deficient
Agata 2006	Japan	Olmesartan	4 weeks	Rat	Cardiac	Not stated	Hypertension
Aguilar 2011	Spain	atorvastatin	8 weeks	Rat	Cardiac	Not stated	Diabetic
Ali 2018	Netherlands	Pioglitazone or Vitamin D	14 days	Rat	Renal	Not stated	Renal Disease
Andersen, 2015	Denmark	Vitamin d	3 weeks	Rat	Not stated	33	Not stated
Araujo 2018	Brazil	Olmesartan	10 days	Hamster	Oral	10	Not stated
Arumugan 2010	Japan	Candesartan	4 weeks	Rat	Cardiac	Not stated	Healthy
Awwad 2019	Egypt	Pregabalin	Not stated	Rat	Cerebral	Not stated	Healthy
Awwad 2019	Egypt	Telmisartan or Captopril	21 days	Rat	Cardiac	8	Heart failure
Azis 2019	Malaysia	Captopril/ Losartan	4 weeks	Rat	Endothelial cells	7	Hypertension
Badae 2019	Egypt	Enalapril	4 weeks	Rat	Cardiac	Not stated	Heart disease
Bernardi 2015	Not stated	Eplerenone	4 weeks	Rat	kidney	75	Renal disease
Bukowska 2017	Germany	Oestrogen	24 hours	Human	Cardiac	36	Heart disease
Burchill 2008	Australia	Ramipril	10 days	Rat	Cardiac	38	Nephrectomy
Chen 2015	China	Perindopril or Losartan	12 weeks	Human	Kidney	110	Diabetic + chronic kidney disease

For peer review only

Chodavarapu 2013	USA	Rosiglitazone	8 weeks	Mouse	Not stated	8	Diabetic
Dong 2019	China	Spironolactone	8 weeks	Rat	kidney	85	Diabetic
Fagyas 2011	Hungary	Enalapril, Perindopril or Ramipril	Not stated	Human	unclear	375	Not stated
Feng 2020	China	Candesartan & Perindopril	4 weeks	Rat	Cerebral	Not stated	Not stated
Flores Monroy, 2016,	USA	Captopril	48 hours	Rat	heart	Not stated	Heart disease
Fuchs 2018	USA	Nicotine	3 weeks	Rat	Cerebral	110	Not stated
Gallagher 2008	USA	Losartan	12 hours	Rat	Cardiac	Not stated	Not stated
Gebska 2013	Poland	Atorvastatin	24 hours	Rat	Cardiac	Not stated	Healthy
Graus-Nunes 2019	Brazil	Losartan or Telmisartan	10 weeks	Mouse	Hepatic	20	Not stated
Guo, 2016	China	Telmisartan	3 weeks	Rat	Pulmonary	Not stated	Not stated
Gupta 2012	USA	Ivabradine	3 months	Dog	Cardiac	24	Heart failure model
Hao 2013,	China	Imidapril	30 days	Chicken	Cardiac	24	Not stated
Hermenegildo 2015	Spain	Estradiol	1 day	Human	Not stated	Not stated	Not stated
Hermenegildo 2018	Argentina	Estradiol	24 hours	Human	Not stated	Not stated	Healthy
Hiroi 2014	Japan	Olmesartan	3 months	Human	Serum	8	HTN
Ibarra-Lara 2016	Mexico	Captopril	21 days	Rat	Cardiac	Not stated	heart disease
Ichikawa 2018	Japan	Olmesartan	2 months	Mouse	Renal	Not stated	Not stated
Igase 2005	USA	Olmesartan, Atenolol, or Hydralazine	14 days	Rat	Cardiac	60	Hypertensive
Igase 2008	Japan	Olmesartan	14 days	Rat	Cardiac	10	Hypertensive
Iizuka 2009	Japan	Nifedipine	Not stated	Human	Cardiac	Not stated	Healthy cells
Ishibashi 2014	Japan	Olmesartan	Not stated	Human	Renal	Not stated	Not stated

For peer review only

Ishiyama 2004	Not known	Losartan Olmesartan	28 days	Rat	Cardiac	62	Post myocardial infarction
Iwanami 2013	Japan	Azilsartan or Olmesartan	4 weeks	Mouse	Cardiac and renal	10	Healthy
Jeong 2018	South Korea	Insulin or pravastatin	8 weeks	Rat	Cardiac	87	Type 1 diabetic
Jessup 2006	USA	Lisinopril or Losartan	12 days	Rat	Cardiac and renal	27	Hypertensive
Jessup 2008	USA	Hydrochlorothiazide	7 days	Rat	Cardiac	48	Hypertensive
Kaiqiang 2009	Japan	Olmesartan	16 weeks	Rat	Cardiac	16	Cardiac hypertrophy
Kidoguchi 2019	Japan	Azilsartan	9 weeks	Rat	Renal	51	Healthy
Kong 2019	China	Spironolactone	Not stated	Rat	Renal	Not stated	Liver Disease
Li 2011	China	Irbesartan	12 weeks	Rat	Cardiac	39	Diabetic
Li 2013	China	Rosuvastatin	29 days	Rat	Cardiac	36	Unclear
Li 2016	China	Valsartan	28 days	Rat	Cardiac	36	Aortic injury
Liang 2015	USA	Telmisartan	35 days	Rat	Cardiac	90	Heart failure
Lijuan 2013	China	Olmesartan	3 days	Mouse	Hepatic	42	Not stated
Lin 2016	China	Calcitriol	Not stated	Rat	Renal	25	Diabetic
Ma 2018	China	Irbesartan, benazepril	12 weeks	Human	Vaginal	36	Not stated
Machado 2014	Brazil	Vitamin d	12 weeks	Rat	Cardiac	Not stated	Hypertension
Malek 2019	India	Telmisartan	4 weeks	Rat	Renal	56	Diabetes
Mao-liang Huang 2009	China	perindopril	4 weeks	Rat	Hepatic	30	Healthy
Marquez 2013	Spain	Insulin	14 days	Rat	Renal	Not stated	Diabetic
Marquez 2014	Spain	Insulin	2 days	Mouse	Renal	Not stated	Healthy
Ocaranza 2006	Chile	Enalapril	8 weeks	Rat	Cardiac	83	Not stated
Ohshima 2014	Japan	Azilsartan	2 weeks	Rat	Cardiac	8	Not stated
Onat 2018	Turkey	Amlodipine or Rosuvastatin	4 weeks	Rat	Renal	Not stated	Hypertension

For peer review only

Qiao 2015	China	Ibuprofen	8 weeks	Rat	Cardiac	18	Diabetes
Riera 2014	Spain	Insulin	40 days	Mouse	Serum, urine and renal	30	Diabetes
Romani-Perez 2015	Spain	Liraglutide	14 days	Rat	Pulmonary	52	Diabetes
Sabry 2018	Egypt	Losartan	6 weeks	Rat	Adipose tissue	48	Diabetes
Salem 2012	USA	Insulin	24 hours	Mouse	Renal	Not stated	Type 1 diabetes
Salem 2013	USA	Insulin	30 days	Mouse	Renal	8	Type 1 diabetes
Salem 2014	USA	Insulin	20 weeks	Mouse	Renal	Not stated	Type 1 diabetes
Sanchez Aguilar 2018	Mexica	Rosiglitazone	7 days	Rat	renal	Not stated	Hypertension
Sanchez-Aguilar 2019	Mexico	Rosiglitazone	7 days	Rat	Cardiac	12	Aortic coarctation model
Scroggin 2012	USA	Rosiglitazone	1 day	Human	Cerebral	Not stated	Not stated
Senador 2010	USA	Losartan	8 weeks	Mouse	Renal	12	Not stated
Shenoy 2009	USA	17b-estradiol (E2)	6 weeks	Rat	Cardiac	18	Bilaterally ovariectomized & hypertensive
Shimada 2011	Japan	Olmesartan	2 weeks	Rat	Cerebral	Not stated	Oestrogen-deficient
Shin 2017	China	insulin	8 weeks	Rat	Cardiac	84	Diabetes
Soler 2009	USA	Telmisartan	2 weeks	Mouse	Renal	11	Not stated
Song 2012	USA	Candesartan	19 days	Rat	Renal	Not stated	Not stated
Speth 2014	USA	Zinc	Real time	Rat	Renal and Pulmonary	Not stated	Not stated
Suh 2019	South Korea	Olmesartan	4 weeks	Mouse	Renal	16	Alport mouse model
Sukumaran 2012	Japan	Telmisartan	4 weeks	Rat	Cardiac	18	Induced cardiomyopathy
Takai 2013	Japan	Valsartan; valsartan & amlodipine	2 weeks	Rat	Cardiac	24	Hypertension

For peer review only

Tanno 2016	Japan	Olmesartan & hydralazine	2 months	Mouse	Cardiac	6	Over-expressing renin
Thanekar 2019	USA	Canagliflozin	15 weeks	Mouse	Urine	Not stated	Diabetes
Varagic 2012	USA	Nebivolol	Not stated	Rat	Cardiac	44	Hypertension
Varagic 2012b	USA	Olmesartan	6 weeks	Rat	Renal	28	Hypertension
Wang 2012	China	Fosinopril	28 days	Rat	Cardiac	32	Heart Disease
Wang 2013	China	Oestrogen	4 weeks	Mouse	Ovarian	17	Hypertension
Wang 2015	China	Sildenafil	30 minutes	Rat	Cardiac	32	Healthy
Wang 2016	China	Enalapril	30 minutes	Rat	Cardiac	32	Healthy
Wang 2016	China	Olmesartan, Candesartan, Telmisartan, Losartan, Valsartan or Irbesartan	Not stated	Mouse	Cardiac	54	Healthy
Wang 2018	China	Telmisartan	12 weeks	Mouse	Cardiac	32	Healthy
Weili 2014	China	Ibuprofen, pioglitazone	8 weeks	Rat	Cardiac	Not stated	Not stated
Wosten-van-Asperen 2011	USA	Losartan	4 hours	Rat	Pulmonary	48	Acute respiratory distress syndrome
Wu 2015	China	Losartan or Enalapril	Not stated	Rat	Cardiac	Not stated	Aortic coarctation model
Xiao 2016	China	Captopril	Not stated	Pig	Not known	Not stated	Not stated
Xu 2017	China	Calcitriol	Not stated	Rat	Pulmonary	30	Lung injury
Yang 2013	China	Enalapril	4 weeks	Rat	Cardiac	25	Hypertension
Yang 2018	China	Telmisartan	24 weeks	Rat		33	Hypertension
Yisireyili 2018	Japan	Irbesartan	2 weeks	Mouse	Intestinal	48	Stress-induced mice
Young 2014		Losartan	72 hours	Mouse	Renal	Not stated	Kidney disease
Zhang 2011	China	Enalapril or Losartan	2 weeks	Mouse	Cardiac	40	Hydronephrotic
Zhang 2013	China	Pioglitazone	24 weeks	Rat	Serum and Hepatic	30	Healthy

For peer review only

Zhang 2014	China	Enalapril or Losartan	4 weeks	Rat	Cardiac	40	Aortic ligation
Zhang 2015	China	Losartan	12 weeks	Rabbit	Cardiac	27	Atherosclerosis
Zhao 2015	China	Linagliptin or liraglutide	28 days	Rat	Cardiac	24	Healthy
Zhao 2019	China	Valsartan	12 weeks	Rat	Cardiac	40	Hypertension
Zhong 2010	China	Telmisartan	10 weeks	Rat	Cardiac	Not stated	Hypertension
Zhong 2011	China	Telmisartan	10 weeks	Rat	Cardiac	40	Hypertension
Zhonghua 2013	China	Telmisartan	4-12 weeks	Human	Not stated	62	Hypertension + Diabetes

For peer review only

Supplementary Material 3: Risk of bias assessment

Study	Was the allocation sequence adequately generated and applied?	Were the groups similar at baseline or were they adjusted for confounders in the analysis?	Was the allocation adequately concealed?	Were the animals randomly housed during the experiment?	Were the caregivers and/or investigators blinded from knowledge which intervention each animal received during the experiment?	Were animals selected at random for outcome assessment?	Was the outcome assessor blinded?	Were incomplete outcome data adequately addressed?	Are reports of the study free of selective outcome reporting?	Was the study apparently free of other problems that could result in high risk of bias?
Abdel-Fattah 2018										
Abdelkader 2020										
Abe 2015										
Abuohashish 2017										
Agata 2006										
Anderson, 2015										
Araujo 2018										
Arumigan 2010										
Awwad 2019										
Awwad 2019										
Badae 2019										
Bernadi 2015										
Bukowska 2017										
Burchill 2008										
Chen 2015										
Chodavarapu 2013										
Dong 2019										
Fangyas 2011										
Feng 2020										
Fuchs 2018										
Furuhashi 2014										
Furuhashi 2015										
Gallagher 2008										
Gebska 2013										
Graus-Nunes 2019										
Guo 2016										
Gupta 2012										

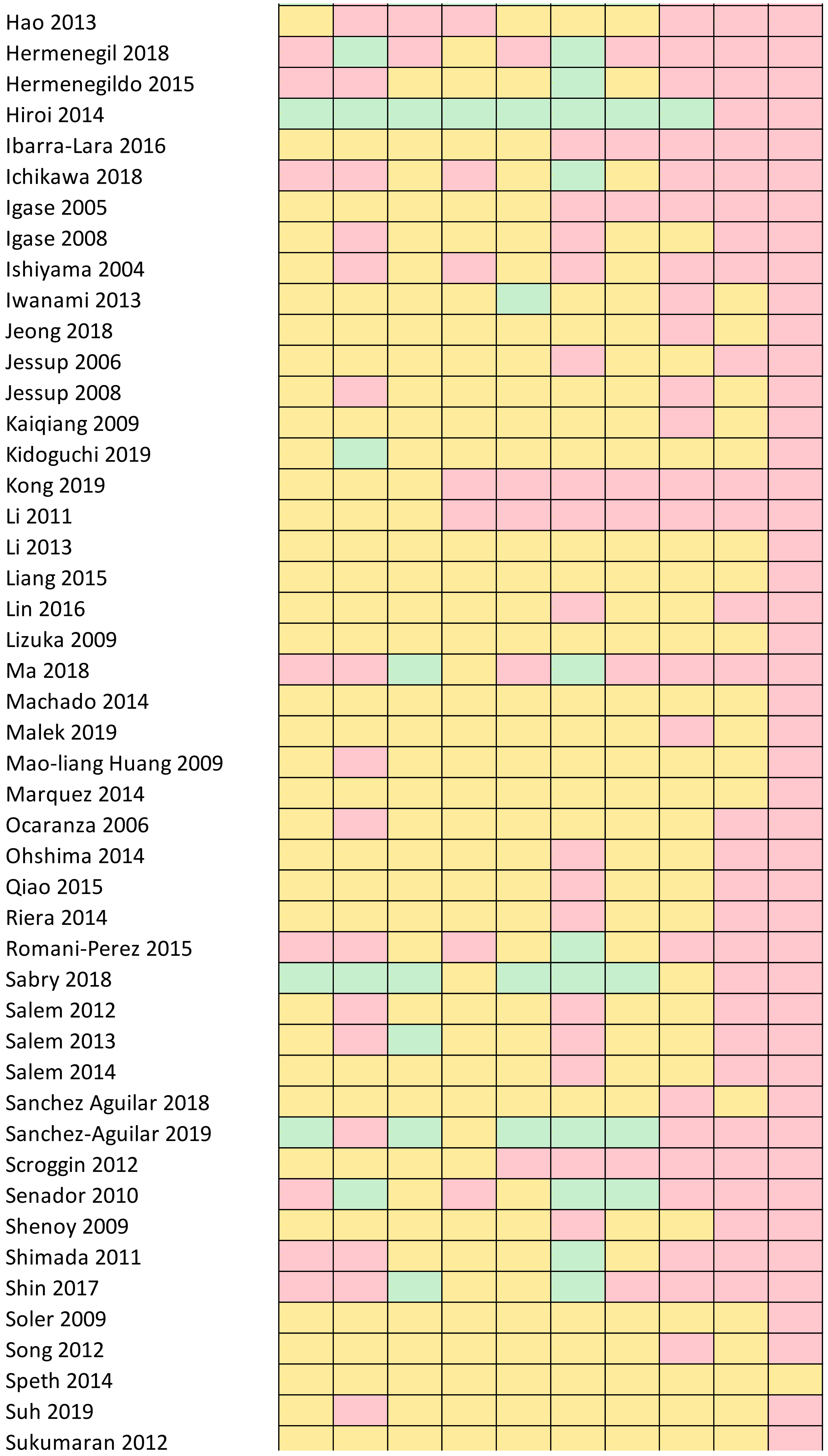
Hao 2013
Hermenegil 2018
Hermenegildo 2015
Hiroi 2014
Ibarra-Lara 2016
Ichikawa 2018
Igase 2005
Igase 2008
Ishiyama 2004
Iwanami 2013
Jeong 2018
Jessup 2006
Jessup 2008
Kaiqiang 2009
Kidoguchi 2019
Kong 2019
Li 2011
Li 2013
Liang 2015
Lin 2016
Lizuka 2009
Ma 2018
Machado 2014
Malek 2019
Mao-liang Huang 2009
Marquez 2014
Ocaranza 2006
Ohshima 2014
Qiao 2015
Riera 2014
Romani-Perez 2015
Sabry 2018
Salem 2012
Salem 2013
Salem 2014
Sanchez Aguilar 2018
Sanchez-Aguilar 2019
Scroggin 2012
Senador 2010
Shenoy 2009
Shimada 2011
Shin 2017
Soler 2009
Song 2012
Speth 2014
Suh 2019
Sukumaran 2012

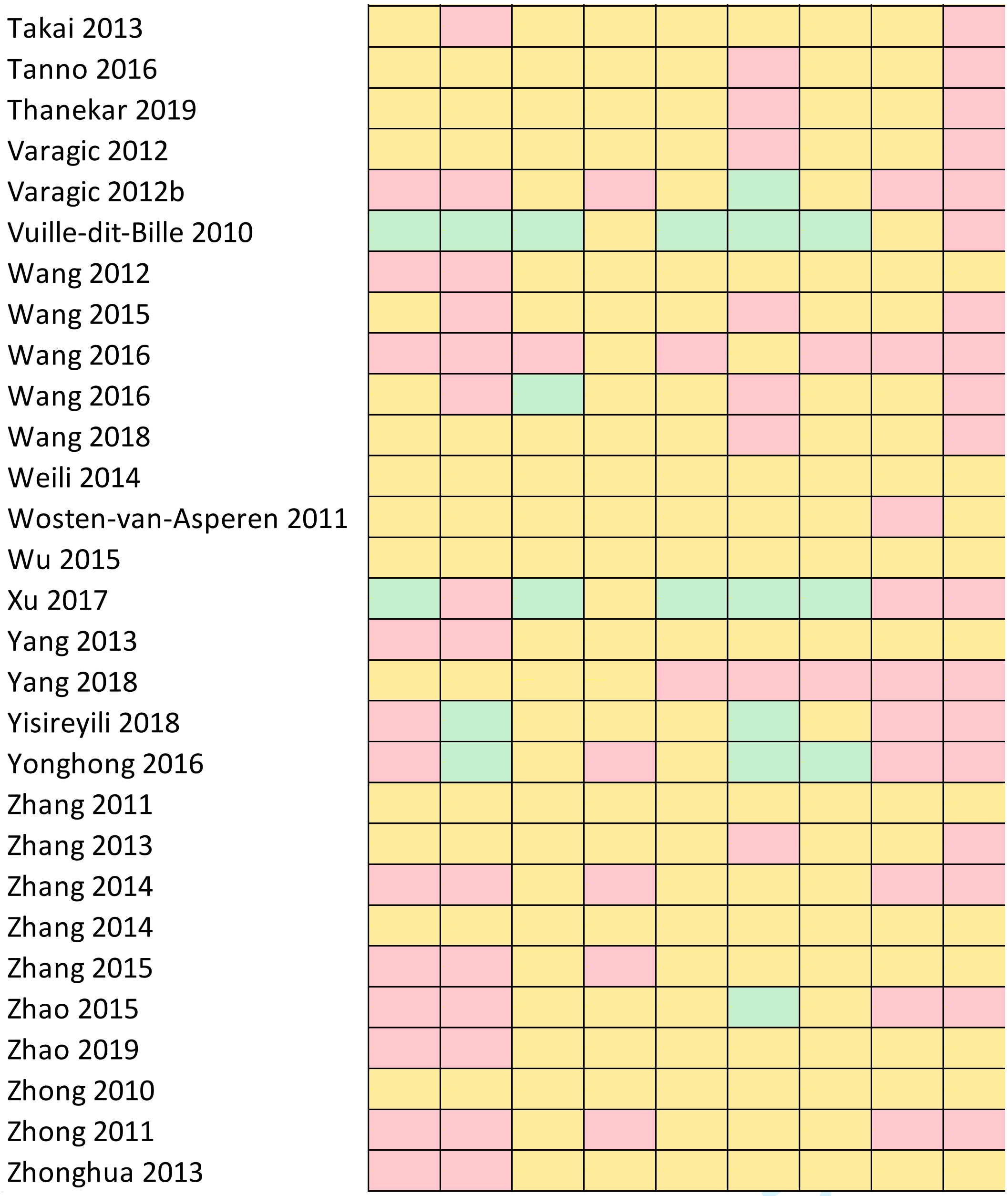
Takai 2013
Tanno 2016
Thanekar 2019
Varagic 2012
Varagic 2012b
Vuille-dit-Bille 2010
Wang 2012
Wang 2015
Wang 2016
Wang 2016
Wang 2018
Weili 2014
Wosten-van-Asperen 2011
Wu 2015
Xu 2017
Yang 2013
Yang 2018
Yisireyili 2018
Yonghong 2016
Zhang 2011
Zhang 2013
Zhang 2014
Zhang 2014
Zhang 2015
Zhao 2015
Zhao 2019
Zhong 2010
Zhong 2011
Zhonghua 2013

PRISMA 2009 Checklist

Section/topic	#	Checklist item	Reported on page #
**TITLE**			
Title	1	Identify the report as a systematic review, meta-analysis, or both.	1
**ABSTRACT**			
Structured summary	2	Provide a structured summary including, as applicable: background; objectives; data sources; study eligibility criteria, participants, and interventions; study appraisal and synthesis methods; results; limitations; conclusions and implications of key findings; systematic review registration number.	2
**INTRODUCTION**			
Rationale	3	Describe the rationale for the review in the context of what is already known.	4
Objectives	4	Provide an explicit statement of questions being addressed with reference to participants, interventions, comparisons, outcomes, and study design (PICOS).	4
**METHODS**			
Protocol and registration	5	Indicate if a review protocol exists, if and where it can be accessed (e.g., Web address), and, if available, provide registration information including registration number.	5
Eligibility criteria	6	Specify study characteristics (e.g., PICOS, length of follow-up) and report characteristics (e.g., years considered, language, publication status) used as criteria for eligibility, giving rationale.	5
Information sources	7	Describe all information sources (e.g., databases with dates of coverage, contact with study authors to identify additional studies) in the search and date last searched.	5
Search	8	Present full electronic search strategy for at least one database, including any limits used, such that it could be repeated.	5 + table 1
Study selection	9	State the process for selecting studies (i.e., screening, eligibility, included in systematic review, and, if applicable, included in the meta-analysis).	5
Data collection process	10	Describe method of data extraction from reports (e.g., piloted forms, independently, in duplicate) and any processes for obtaining and confirming data from investigators.	5
Data items	11	List and define all variables for which data were sought (e.g., PICOS, funding sources) and any assumptions and simplifications made.	5
Risk of bias in individual studies	12	Describe methods used for assessing risk of bias of individual studies (including specification of whether this was done at the study or outcome level), and how this information is to be used in any data synthesis.	5
Summary measures	13	State the principal summary measures (e.g., risk ratio, difference in means).	5
Synthesis of results	14	Describe the methods of handling data and combining results of studies, if done, including measures of consistency (e.g., I^2) for each meta-analysis.	5

For peer review only - http://bmjopen.bmj.com/site/about/guidelines.xhtml

PRISMA 2009 Checklist

Section/topic	#	Checklist item	Reported on page #
Risk of bias across studies	15	Specify any assessment of risk of bias that may affect the cumulative evidence (e.g., publication bias, selective reporting within studies).	5
Additional analyses	16	Describe methods of additional analyses (e.g., sensitivity or subgroup analyses, meta-regression), if done, indicating which were pre-specified.	none
**RESULTS**			
Study selection	17	Give numbers of studies screened, assessed for eligibility, and included in the review, with reasons for exclusions at each stage, ideally with a flow diagram.	6
Study characteristics	18	For each study, present characteristics for which data were extracted (e.g., study size, PICOS, follow-up period) and provide the citations.	6 +table 2
Risk of bias within studies	19	Present data on risk of bias of each study and, if available, any outcome level assessment (see item 12).	Table 4
Results of individual studies	20	For all outcomes considered (benefits or harms), present, for each study: (a) simple summary data for each intervention group (b) effect estimates and confidence intervals, ideally with a forest plot.	Table 2 and 3 page 6
Synthesis of results	21	Present results of each meta-analysis done, including confidence intervals and measures of consistency.	No meta-analysis
Risk of bias across studies	22	Present results of any assessment of risk of bias across studies (see Item 15).	Table4
Additional analysis	23	Give results of additional analyses, if done (e.g., sensitivity or subgroup analyses, meta-regression [see Item 16]).	None
**DISCUSSION**			
Summary of evidence	24	Summarize the main findings including the strength of evidence for each main outcome; consider their relevance to key groups (e.g., healthcare providers, users, and policy makers).	7
Limitations	25	Discuss limitations at study and outcome level (e.g., risk of bias), and at review-level (e.g., incomplete retrieval of identified research, reporting bias).	8
Conclusions	26	Provide a general interpretation of the results in the context of other evidence, and implications for future research.	8
**FUNDING**			
Funding	27	Describe sources of funding for the systematic review and other support (e.g., supply of data); role of funders for the systematic review.	9

From: Moher D, Liberati A, Tetzlaff J, Altman DG, The PRISMA Group (2009). Preferred Reporting Items for Systematic Reviews and Meta-Analyses: The PRISMA Statement. PLoS Med 6(7): e1000097. doi:10.1371/journal.pmed1000097

For peer review only - http://bmjopen.bmj.com/site/about/guidelines.xhtml

PRISMA 2009 Checklist

For more information, visit: www.prisma-statement.org.

Page 2 of 2

For peer review only
